# Explicit characterization of human population connectivity reveals long run persistence of interregional dengue shocks

**DOI:** 10.1098/rsif.2020.0340

**Published:** 2020-07-22

**Authors:** Lim Jue Tao, Borame Sue Lee Dickens, Mao Yinan, Chae Woon Kwak, Ng Lee Ching, Alex R. Cook

**Affiliations:** 1 Saw Swee Hock School of Public Health, National University of Singapore and National University Health System, Singapore, Singapore; 2 Environmental Health Institute, National Environmental Agency, Singapore, Singapore

**Keywords:** dengue, penalized estimation, spatio-temporal statistics, multivariate time series

## Abstract

Dengue is hyper-endemic in Singapore and Malaysia, and daily movement rates between the two countries are consistently high, allowing inference on the role of local transmission and imported dengue cases. This paper describes a custom built sparse space–time autoregressive (SSTAR) model to infer and forecast contemporaneous and future dengue transmission patterns in Singapore and 16 administrative regions within Malaysia, taking into account connectivity and geographical adjacency between regions as well as climatic factors. A modification to forecast impulse responses is developed for the case of the SSTAR and is used to simulate changes in dengue transmission in neighbouring regions following a disturbance. The results indicate that there are long-term responses of the neighbouring regions to shocks in a region. By computation of variable inclusion probabilities, we found that each region’s own past counts were important to describe contemporaneous case counts. In 15 out of 16 regions, other regions case counts were important to describe contemporaneous case counts even after controlling for past local dengue transmissions and exogenous factors. Leave-one-region-out analysis using SSTAR showed that dengue transmission counts could be reconstructed for 13 of 16 regions' counts using external dengue transmissions compared to a climate only approach. Lastly, one to four week ahead forecasts from the SSTAR were more accurate than baseline univariate autoregressions.

## 1. Introduction

Malaysia and Singapore are populated by around 38 million people. In both countries, dengue is classified as hyper-endemic due to all four dengue serotypes being in active circulation, putting most individuals at risk of dengue infection, and creating considerable health and economic burdens [1,2]. Widespread urbanization, favourable climatic conditions and increased human mobility across highly connected cities such as Singapore and Kuala Lumpur are ideal for dengue transmission [3,4], as reflected in the long-term non-zero weekly counts in Singapore and all administrative regions in Malaysia [5]. The use of the only licensed vaccine, Dengvaxia (CYD-TDV), is problematic given low seroprevalence rates especially among younger age groups due to past history of successful vector control [6,7]. As a result, vector control remains the primary public health intervention, targeting the two dominant dengue mosquito vectors, *Aedes aegypti* and *Ae. albopictus*, which are endemic to both countries [5,8]. Vector control means include community outreach, breeding site reduction and, more recently, *Wolbachia*, for epidemic prevention and control [5,9]. The timing and scale of these interventions are critical for effective epidemic mitigation and warning systems and require an understanding of the spatial patterns of dengue transmission and the risk of spillover of epidemics from one region to another.

Southeast Asia has increasing levels of connectivity and human movement which has contributed to elevated mortality and high economic losses from past pandemics such as H1N1 [10]. Substantial literature exists on using mathematical models to describe spatio-temporal patterns of disease behaviour such as hand-foot-and-mouth disease and Ebola through animal and human host movements respectively [11,12]. Statistical approaches include space–time Bayesian hierarchical modelling, cluster and network analysis between geographical regions [13–15]. However, these often have a substantial computational burden or suffer from dimensionality issues due to the temporal persistence of disease transmissions and large number of regions, which result in a large number of parameters to describe the spatial, temporal and spatio-temporal patterns.

Space–time recursive autoregressions (STARs) are used for geographical, financial and economic modelling where multiple time series have to be estimated simultaneously [16] while taking into account spatio-temporal dependencies. This approach overcomes the constraint of having a substantial number of spatial parameters by subsuming them under a weight matrix describing geographical distance and/or connectivity across regions. While vector autoregression is a model structure which can capture interdependencies among multiple evolving variables—such as disease case counts across regions—using its own lagged values and that of the other model variables [17], the STAR is a special case with additional spatial terms accounting for these same endogenous variable interactions between regions. Even though between-region dependencies are subsumed within the weight matrix, a large number of parameters can still be individually estimated for an appropriate specification in disease dynamics from complex interdependencies between climate, dengue transmission counts and inter-regional transmissions over long time lags. In this paper, we propose regularization as a potential solution as penalties are imposed on a STAR specification to induce sparsity, thereby creating what we denote a sparse space–time recursive autoregression (SSTAR).

Dengue case warning systems and predictions have been carried out multiple times for both Singapore [18] and Malaysia [19]. Yet, there has been limited exploration on the effects of importation between the Malaysian regions and Singapore through human movement. The two countries lie on the Malay peninsula and part of Borneo, with Malaysia having 13 administrative regions on the former and three on the latter. Singapore is highly connected to Malaysia through two crossings linking Pulau Ujong to Johor, with a daily movement of 300 000 individuals, comprising approximately 5% of Singapore’s total population. Additionally, the flight route between Kuala Lumpur and Singapore is the busiest in the world, with 30 537 flights made annually [20,21]. Once an infectious person enters a region in Malaysia or Singapore, there is potential for local transmission to occur with subsequent infections and a potential outbreak due to the presence of the vector and suitable vector breeding conditions [22]. With such multipath complexities, dengue transmission within Malaysia and between Malaysia and Singapore should be considered contemporaneously to better understand the observed dengue transmission counts in each region.

This paper, therefore, explores the use of STAR and SSTAR to investigate the inter-regional dynamic signature of dengue between Malaysia and Singapore. We aim to estimate the level of dengue transmissions in each region attributable to other regions, while controlling for local dengue dynamics, and create a regional forecasting model for estimating changes in dengue transmission. To this end, we first examine the descriptive ability of inter-regional counts on each region using Granger causality test (GCT) and likelihood ratios. Next, using the STAR and SSTAR models, we ascertain how attributable previous dengue transmissions within or outside of a region are on local dengue transmission using forecast error impulse response analysis. Models are then assessed according to their ability to recreate each region’s dengue transmission count density using external dengue counts and locally observed climatic factors in a quasi-imputation approach. We forecast one to four weeks ahead with the mean absolute error (MAE) criterion used to compare the predictive ability of STAR versus SSTAR for each region. Lastly, variable importance and statistical inference on variables which describe dengue transmission for each region are conducted using a block-bootstrap procedure.

## 2. Methods

### 2.1. Dengue incidence data

**Figure 1.**
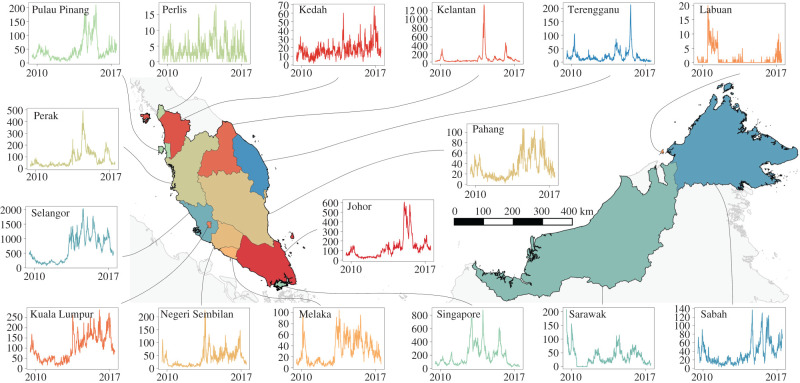
Weekly dengue transmission counts in all administrative regions of Malaysia and Singapore from 2010 to 2017.

Dengue incidence data in Singapore were collected by the Ministry of Health, with mandatory notification of virologically confirmed or laboratory-confirmed cases. Data were publicly available from the Infectious Disease Bulletin, published weekly by the Ministry of Health, Singapore. Dengue incidence data in Malaysia by administrative region were collected by the Ministry of Health, Malaysia, and publicly available on [23] (figure 1). In both Singapore and Malaysia, case counts are reported through passive surveillance from healthcare institutions from each region. To account for the difference in population sizes among different regions and further allow for interpretation of the regression coefficients, we normalized dengue case counts to a scale of 0 to 1 for the analysis by subtracting each region’s observed case counts by its minimum value and dividing it by the range of values each region observes. First differencing of case counts was then conducted to ensure stationarity in data. Data were available for 15 regions in Malaysia and Singapore from 2010 to 2017. No ethical approval was required for this study.

### 2.2. Climate data

Climate data were obtained from ERA5, published by the European Centre for Medium-Range Weather Forecasts. ERA5 provided hourly estimates across a 30 km grid [24], which we aggregated over a weekly timescale and spatially averaged for Singapore and regions of Malaysia. Mean, minimum and maximum air and dewpoint temperature at 2 m as well as weekly total rainfall are collected. Saturation vapour pressure and actual vapour pressure are also collected using Teten’s formula, whence relative and absolute humidity were derived using standard formula [25]. These are biologically relevant variables for dengue transmission dynamics as they impose thermal forcing and stress on vector population growth and influence breeding site availability.

### 2.3. Land connectivity data

Land connectivity data were obtained by querying the Google Maps API for distances between namesake cities in the administrative regions in Malaysia and Singapore, which provides the total time and distance travelled between regions. Land connectivity data is used to explicitly model spatio-temporal dynamics of dengue in Malaysia and Singapore as described below.

### 2.4. Space–time recursive autoregression

We built reduced form STAR models [16] (2.1) with scaled differenced dengue incidence of Singapore and all administrative regions of Malaysia as the dependent variable *y*_*t*,*k*_, where *t* denotes time, *k* denotes the region. The explanatory variables used are the *P*_1_th order lagged scaled differenced dengue incidence within region *k*, denoted as $y_{t-i_{1},k}$, *i*_1_ = 1, …, *P*_1_, the *P*_2_th order lagged scaled differenced dengue incidence in regions outside *k*, denoted as $y_{t-i_{2},-k}$, *i*_2_ = 1, …, *P*_2_, and exogenous climatic variables with $P{\mathrm{th}}_{3}$ order time lag, denoted as $X_{t-i_{3},u}$, *i*_3_ = 1, …, *P*_3_, where *u* is a subscript denoting the different types of climatic variables considered, such as absolute humidity or precipitation. In addition, the STAR requires a weighted sum of all other regions parameterized both by dengue counts for other regions through a connectivity matrix *W*, which will be described in §2.3. Correspondingly, $\theta \in \{\phi_{i_{1},k},\gamma_{i_{2},k},c_{i_{3},u,k}\}$ are the autoregressive, spatial autoregressive and climatic coefficients to be estimated for a maximum of *P*_1_, *P*_2_, *P*_3_ time lags respectively. Finally, *ε*_*t*_ is a noise term which affects all equations of the STAR.

The STAR is fit sequentially using equation-by-equation ordinary least squares or feasible generalized least squares (FGLS). FGLS is conducted by re-weighting estimands by the estimated residual variance to account for possible serial correlation in each region [17]. Variables include one to 10 week lag terms for past dengue transmissions and past climatic factors as independent variables. The number of lags was chosen to represent the maximum life cycle of the *Ae. aegypti* vector in addition to the incubation and infectious period of the dengue virus [5,7] and further addition of lag terms would possibly lead to spurious auto-correlation in statistical inference [17]. The full dimensions of the specification across equations are described in electronic supplementary material, S1. The number of lags are selected for each equation using an AIC selection procedure, to balance model fit and parsimony [26].

2.1 $$\begin{array}{r} y_{t,k}=\sum_{i_{1}=1}^{P_{1}}\phi_{i_{1},k}y_{t-i_{1},k}+\sum_{i_{2}=1}^{P_{2}}\gamma_{i_{2},k}Wy_{t-i_{2},-k}+\sum_{u=1}^{H}\sum_{i_{3}=1}^{P_{3}}c_{i_{3},u,k}X_{t-i_{3},u}+\epsilon_{t}. \end{array}$$

### 2.5. Penalized estimation of STAR

While STARs are able to estimate the effects of dengue transmission counts across different regions, having an additional set of dependent variables or time series drastically increases the number of parameters that need to be estimated. For example, having an additional lag term within our STAR would increase the number of coefficients to estimate by the number of time series, while having an additional time series added to our model would increase the number of coefficients to estimate by the number of coefficients within the observation equation. Within (2.2), we would have 16 regions and 10 maximum lags, estimating a plausible number of 320 coefficients. In this case, equation by equation ordinary least squares estimation of inter-regional dengue transmission would lead to unimportant but non-zero coefficients. This may overestimate the true inter-regional effect of dengue transmission and poorly forecast dengue transmission out of sample [27].

We thus conduct regularized estimation of our STAR specification, leading to SSTAR, which is a solution that provides a jointly parsimonious and interpretable model of inter-regional dengue transmissions. Inducing sparsity will allow for automatic variable selection among the many covariates with a large number of lags, and can still provide valid inferences on dengue transmission dynamics. Furthermore, it can allow better out-of-sample predictive performance [27]. This estimation extends the equation-by-equation OLS minimization problem to include some penalty term for our coefficients, with a tuning parameter *λ* being estimated by a cross-validation scheme and the overall objective function minimized (2.2) using cyclical coordinate descent [28]. For our model problem, we used fivefold cross validation due to its ability to balance test error estimation bias and variance [27]. Fivefold cross validation was conducted by first partitioning our data to five samples of equal length, with a single subsample retained as the test set. The other subsamples are used to train the SSTAR model. This is repeated five times to produce cross-validation error over various *λ* values. We lastly refitted the SSTAR using the optimal regularization parameter *λ*.

2.2 $$\begin{array}{c} \underset{\phi ,\gamma ,c\lambda }{\arg\min}[\text{∥}y_{t,k}-\sum_{i_{i}=1}^{P_{1}}\phi_{i_{1},k}y_{t-i_{1},k}-\sum_{i_{2}=1}^{P_{2}}\gamma_{i_{2},k}Wy_{t-i_{2},-k}.\;\\ \;-\sum_{u=1}^{H}\sum_{i_{3}=1}^{P_{3}}c_{i_{3},u,k}X_{t-i_{3},u}{∥}^{2}+\lambda ∥\phi ,\gamma ,c∥].\; \end{array}$$

### 2.6. Choice of weight matrices

We consider two choices of weight matrices *W* with diagonal entries being 0 for our model problem. First, we consider a connectivity matrix with $w_{r_{1},r_{2}}$ entry being the scaled inverse distance between the regions *r*_1_ and *r*_2_. Second, we consider an adjacency matrix with $w_{r_{1},r_{2}}=1/card\left(\mathrm{adjacent}\;\mathrm{regions}\right)$ so long as the *r*_1_th region is one of the three closest neighbours to the *r*_2_th region, based on the average number of regions which share common borders (figure 1).

2.3 $$W=\left[\begin{array}{cccc} 0 & w_{1,2} & \ldots & w_{1,16}\\ w_{2,1} & 0 & \ldots & w_{2,16}\\ \vdots & \vdots & \ddots & \vdots \\ w_{16,1} & w_{16,2} & \ldots & 0 \end{array}\right]$$

### 2.7. Model inference

Coefficients in the STAR and SSTAR are difficult to interpret, unlike those in the canonical univariate autoregressive models. Lagged relationships across regions and time mean that standard interpretations for the canonical univariate time series do not apply. We use the forecast error impulse responses (FEIR) as a tool to interpret the models [29]. The FEIR is manifested through the impact of a shock on the noise term *ε*_*t*,*j*_ (on **Y**_*t*+*h*_ for a region *j*) on the expected value of case counts for each other region at a future time horizon *t* + *h*. A unit shock *δ*_*j*_ on some region and the subsequent FEIR value on other regions can be thought of as the effect of how an increase in the shocked regions counts on other regions case counts over time. As these procedures are normally reserved for time-dependent only vector autoregressions, we modify these procedures for the STAR and SSTAR. The full computational details to derive the FEIR to incorporate the spatio-temporal dependencies in STAR and SSTAR are described in electronic supplementary material, S2 and S3.

### 2.8. Model assessment

We first conduct GCTs [30] to ascertain whether the connectivity/adjacency matrix would have any effect on dengue transmission counts within each region, independent of climatic variables and past dengue counts within the same region. Next, we conduct a quasi-imputation experiment by leaving one region out in our analysis to ascertain the importance of connecting regions on the region of interest. To do so, we build SSTAR models on a training set comprising the first third of the observed dataset *T*_*j*,train_ = *T*/3 which considers present and past climate only (2.4), connectivity/adjacency matrix only (2.5) or climate and the connectivity/adjacency matrix (2.6) as the explanatory variable to dengue transmission counts for each region. We then use the estimated coefficients to impute dengue counts for the remaining dataset *T*_*j*,test_ = 2*T*/3 as though the region’s dengue counts were not known for the test set. The imputed densities using (2.4)–(2.6) are then compared to the observed density for the test set to assess concordance between the four densities.

2.4 $$y_{t,k}=\sum_{u=1}^{H}\sum_{i_{3}=1}^{P_{3}}c_{i_{2},u,k}X_{t-i_{3},u}+\epsilon_{t},$$

2.5 $$y_{t,k}=\sum_{i_{3}=1}^{P_{2}}\gamma_{i_{2},k}Wy_{t-i_{2},-k}+\epsilon_{t}$$

2.6 $$\text{and}\;y_{t,k}=\sum_{i_{3}=1}^{P_{2}}\gamma_{i_{2},k}Wy_{t-i_{2},-k}+\sum_{u=1}^{H}\sum_{i_{3}=1}^{P_{3}}c_{i_{2},u,k}X_{t-i_{3},u}+\epsilon_{t}.$$

Third, we assess parameter stability and variable importance by subjecting our SSTAR estimation strategy to bootstrapping. This step provides bootstrap confidence intervals for parameters of interest and the degree of penalization each parameter receives [27]. Bootstrapping is conducted over 1000 iterations by resampling our data randomly for each region with replacement and then reestimating our SSTAR model at each step. The coefficients re-estimated by the SSTAR at each bootstrap iteration are used to construct bootstrap confidence intervals for our explanatory variables, as well as posterior inclusion probabilities which provides the number of times SSTAR penalizes our explanatory variables to zero.

Lastly, an assessment of one to four week ahead forecasting ability is conducted for each region in question for both the canonical STAR as well as our proposed SSTAR procedure. This was conducted to assess the out of sample performance of the models. We first take a training set comprising the first third of observed dengue transmissions *T*_*j*,train_ = *T*/3 from 2010 to 2013 and re-estimate our model in a rolling manner over every time point in the test set from 2014 to 2017 for the STAR using ordinary and feasible generalized least squares (FGLS), and for SSTAR using the rolling cross-validation scheme detailed in §2.4. Additionally, we compared the models described above against the canonical autoregressive model, estimated using ordinary and feasible generalized least squares procedures with number of lag terms selected using the Akaike information criterion [26], which balances model fit and parsimony.

We compute the STAR against the baseline SSTAR model over one to four weeks ahead in relative mean square forecast error (rMAE). To compute the rMAE, we follow [31] by first generating one to four week ahead forecasts for the competing models by aggregating every two to four week variable observation. A one step ahead forecast for the weekly and two to four weeks aggregated data then allows us to get one to four week ahead forecasts for each of the models. All exogenous climate variables are included within the forecasting framework. If *y*_*t*+*l*,*j*_ denotes the actual number of dengue cases *l* weeks after time *t* where the prediction was made for the *j* region and ${\hat{y}}_{t+l,j,M}$ the number of cases predicted by the model *M*, the MAE is given by

2.7 $${\text{MAE}}_{l,j,M}=\sum_{t\in V}∥y_{t+l,j}-{\hat{y}}_{t+l,j,M}∥,$$

where *V* denotes the test set of observations from 2014 to 2017. The rMAE was then computed by taking the ratio of MAEs between two competing models *M*_1_ and *M*_2_ (2.8). An rMAE higher than 1 would signify that *M*_1_ has a higher forecasting error compared to *M*_2_ in the test set of observations, whereas an rMAE lower than 1 would signify that *M*_1_ has a lower forecasting error compared to *M*_2_ in the test set of observations.

2.8 $${\text{rMAE}}_{l,j,M_{1},M_{2}}=\frac{{\text{MAE}}_{l,j,M_{1}}}{{\text{MAE}}_{l,j,M_{2}}}.$$

## 3. Results

### 3.1. Weight matrix generation

**Figure 2.**
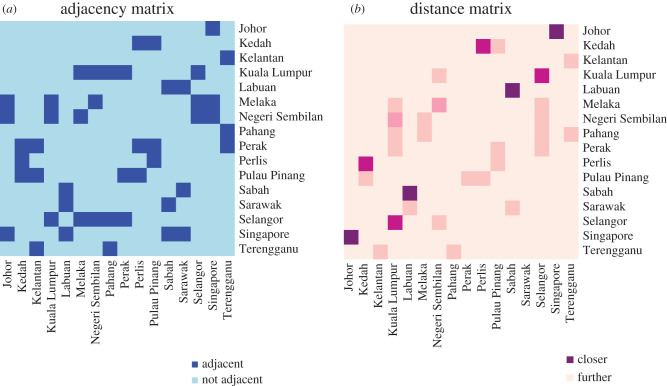
Illustration of two proposed connectivity matrices from left to right: (*a*) adjacency matrix using the maximal number of adjacent localities; (*b*) connectivity matrix using scaled inverse distance.

By using land connectivity data obtained from the Google Maps API, we computed the adjacency/connectivity matrices based on (2.3), setting the number of closest regions to each respective region **card**(adjacent regions) = 3, based on the average number of adjacent counties which share common borders in figure 1. Whereas the connectivity matrix indicated that at most four regions are near to a single region. The connectivity matrix also indicates that the regions with the closest namesake cities to one another are Singapore and Johor, Labuan and Sabah as well as Kuala Lumpur and Selangor (figure 2).

### 3.2. Model adequacy

GCTs indicated that other regions counts are significantly Granger caused to dengue transmission counts in eight out of 16 regions: Johor, Kedah, Negeri Sembilan, Pahang, Perak, Sarawak, Singapore and Terengganu through the connectivity matrix parameterization (table 1). In five out of 16 regions, the GCT was also significant on the adjacency matrix parameterization, namely: Kedah, Pulau Pinang, Sabah, Sarawak and Singapore. In six regions, namely: Kelantan, Kuala Lumpur, Labuan, Melaka, Perlis and Selangor, none of the GCT for each matrix were significant, which indicates that for a majority of regions, dengue case counts from other regions may be Granger causing locally. A likelihood ratio test comparing a linear model with only past dengue counts and climatic variables versus the STAR also showed that the observed distribution of dengue transmissions to be better explained with the STAR in four out of 16 regions: Johor, Pahang, Selangor and Singapore. (table 1)

**Table 1.** Univariate Tests.^*a*^

	GCT (connectivity matrix)	GCT (adjacency matrix)	LRT
Johor	0.00*	0.39	0.01*
Kedah	0.00*	0.04*	0.72
Kelantan	0.85	0.32	0.12
Kuala Lumpur	0.69	0.13	0.11
Labuan	0.10	0.19	0.09
Melaka	0.42	0.47	0.25
Negeri Sembilan	0.05*	0.91	0.18
Pahang	0.05*	0.84	0.00*
Perak	0.02*	0.96	0.09
Perlis	0.19	0.38	0.25
Pulau Pinang	0.77	0.00*	0.58
Sabah	0.23	0.00*	0.98
Sarawak	0.02*	0.05*	0.57
Selangor	0.07	0.14	0.00*
Singapore	0.00*	0.04*	0.02*
Terengganu	0.00*	0.83	0.28

^a^Granger causality test (GCT) was conducted at the 95% level on the connectivity/adjacency matrix and local dengue transmission counts, likelihood ratio test (LRT) was conducted by comparing the linear model using local dengue transmission counts and climate against the STAR.

**Figure 3.**
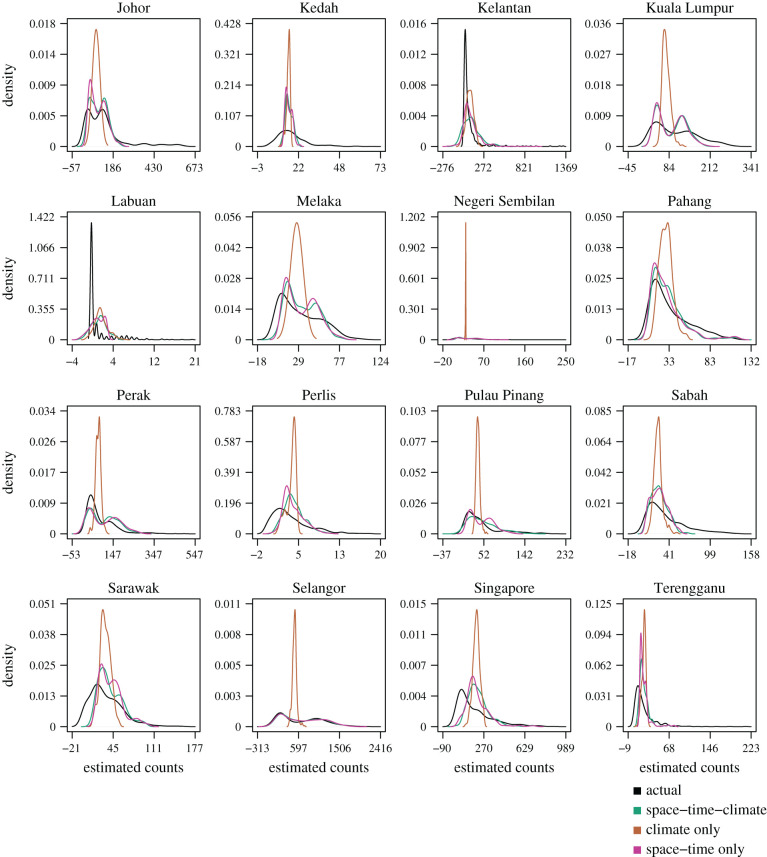
Imputation densities. Lines represent imputed densities from the SSTAR models with climate, climate only SSTAR models and SSTAR models without climate against the empirical density.

The quasi-imputation experiment provides a measure of how well connectivity and counts observed in other regions may describe counts within a region even in the absence of observing their dengue case counts for a given period of time. We inspected imputed densities across three SSTAR specification with only connectivity, climate of the region of interest and the combination of both against actual observed counts. The experiment showed that SSTAR imputed densities were better described using the connectivity matrix with climate compared to either connectivity or climate variable only models for 13 out of 16 regions: Johor, Kuala Lumpur, Melaka, Negeri Sembilan, Pahang, Perak, Perlis, Pulau Pinang, Sabah, Sarawak, Selangor, Singapore and Terengganu (figure 3).

**Figure 4.**
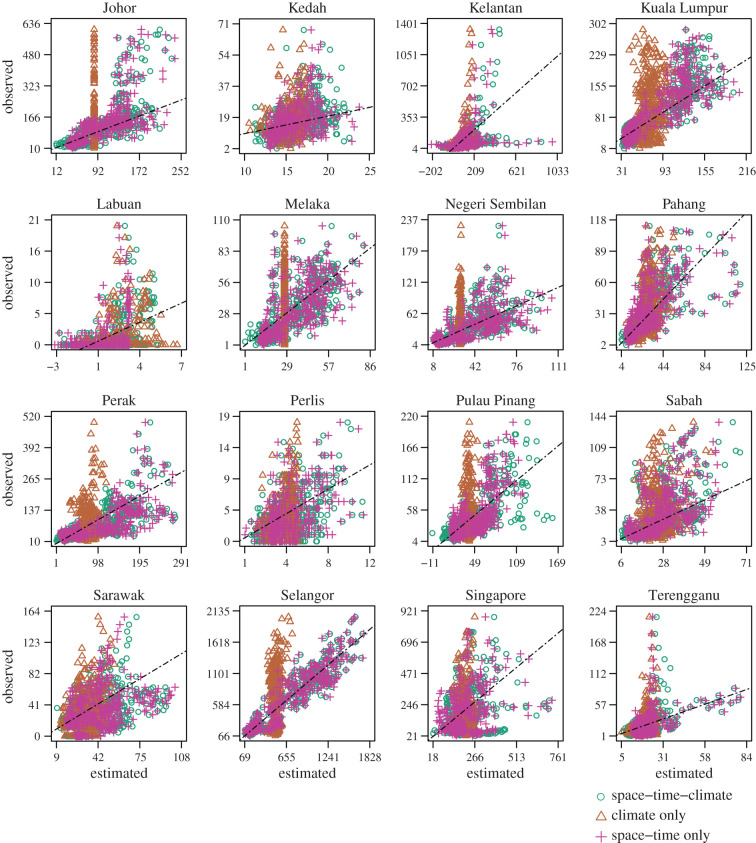
Observed versus predicted counts. Points represent imputed dengue case counts from the SSTAR models with climate, climate only SSTAR models and SSTAR models without climate against the observed.

Highly sharp and irregular imputed densities of the climate only SSTAR model across every region showed that only including climate would fail to characterize spread of the actual count density, while in general the addition of other regions' counts help to push down the imputed density to emulate the observed count density for the region of interest better (figure 3). Plotting the observed counts against imputed counts for all three models across regions also indicate that imputed points lie closer to the line of equality for low observed values across all model. However, as observed values of dengue case counts increase, the climate only model deviates further away from the line of equality while the models including other regions counts and/or climate adhere to that line but tend to underestimate the true observed count values (figure 4).

### 3.3. Forecast performance

SSTAR was able to beat 3 other comparison models, namely the STAR, feasible generalized least squares estimated STAR-2, feasible generalized least squares estimated autoregressive (FGLS-AR) as well as the canonical autoregressive model (AR) on all regions for one to four week ahead forecast as indicated by a relative MAE which is smaller than 1. In general, improvements in forecasting error increase as the forecast horizon increases for the proposed SSTAR model, with up to 18% improvement in forecast performance at one week ahead against the STAR and FGLS-AR models across all administrative regions. Two to four week ahead improvements in forecast performance similarly increased with the most improvements made against the baseline STAR movement at 34%, 47% and 56% respectively. In terms of absolute MAE, the SSTAR has an average error of around 0.52 to 43.29 for one week ahead forecasts for the regions of Labuan and Selangor respectively. The largest errors in terms of absolute MAE are for three week ahead forecasts for Selangor at 72.03 (electronic supplementary material, S4–5) In comparison, the MAE for the baseline AR model is at around 0.69–49.19 for one week ahead forecasts for the regions of Labuan and Selangor, respectively. The largest errors in terms of absolute MAE are for three week ahead forecasts for Selangor at 93.55 (electronic supplementary material, S4–5).

### 3.4. Covariate importance

**Figure 5.**
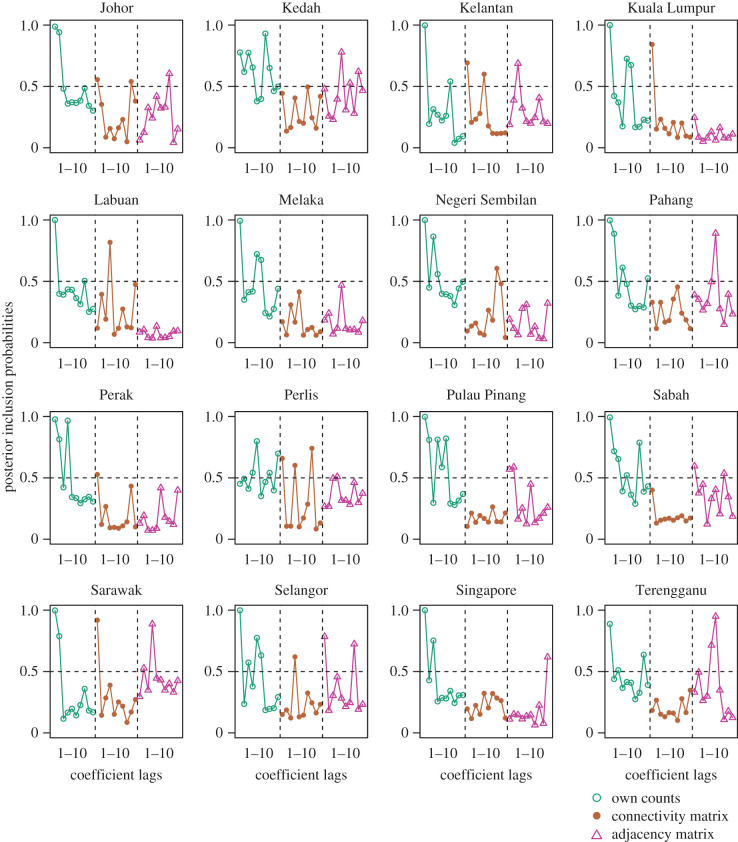
Posterior inclusion of autoregressive terms. Points represent posterior inclusion probabilities for 1 to 10 lagged values of own dengue transmission counts, connectivity and adjacency weighted counts for the full SSTAR model with climate across each region.

Bootstrapping SSTAR over 1000 iterations by rerunning the SSTAR over subsamples provided a measure of variable significance. Ninety-five per cent bootstrap intervals of connectivity/adjacency matrix for SSTAR showed that there is at least one lag term whose interval did not cross zero for all regions (electronic supplementary material, S5). Likewise, the bootstrap inclusion probabilities which provide a measure of variable importance indicated that the regions with connectivity matrix lags above the 0.5 probability cut-off on at least one occasion are Johor, Kedah, Kelantan, Kuala Lumpur, Labuan, Negeri Sembilan, Pahang, Perlis, Pulau Pinang, Sabah, Sawarak, Selangor, Singapore and Terengganu. Regions with no connectivity matrix lags below the 0.5 cut-off are Melaka and Perlis (figure 5). In general, the adjacency matrix was included more often in bootstrap samples compared to the connectivity matrix in 11 of 16 regions: Johor, Kedah, Melaka, Pahang, Perlis, Pulau Pinang, Sabah, Sarawak, Selangor, Singapore and Terranganu. Inclusion of each region's own counts were also consistently above non-zero for at least one lag overall.

For climate variables, at least one lag of absolute and relative humidity have 95% bootstrap intervals which do not overlap with 0 for all regions, with the direction of their coefficients being mixed. In Kedah, absolute humidity had negative effects on incidence at lag 6 and 8, but relative humidity had positive effects at lag 5. On the contrary, in Melaka, absolute humidity had a positive effect at lags 7 and 8 and relative humidity had a positive effect at lag 4. In 15 of 16 regions, at least one lag of temperature have 95% bootstrap intervals which do not overlap with 0; only Negeri Sembilan had 95% bootstrap intervals which overlap with 0 for all temperature lags. In seven regions, all lags of precipitation have 95% bootstrap intervals which overlap with 0. These include Kedah, Kelantan, Kuala Lumpur, Melaka, Negeri Sembilan, Singapore and Terengganu (electronic supplementary material, S5).

### 3.5. Shock persistence

**Figure 6.**
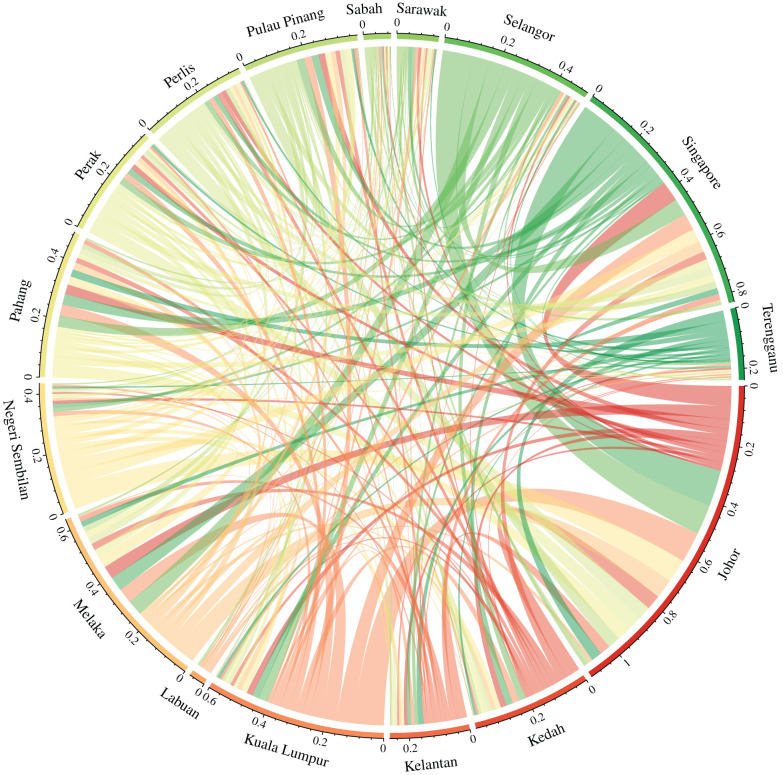
Forecast error cumulative response functions across regions: lines corresponding to sector colours represent the effect of a 1 s.d. shock on the sector's dengue transmission counts on other regions. Width of lines represent total 50 week ahead cumulative forecast error impulse response from shocked region to another region.

Lastly, FEIR analyses revealed long run shock persistence of dengue transmission across most regions when a particular region is subject to a 1 s.d. shock (electronic supplementary material, S1). However, for regions where connectivity is not indicatively important on the assessment measures above (such as Labuan), shocking Labuan would lead to an almost instantaneous reversion to zero for all other regions, which indicates that a large increase or decrease in that region’s counts would not affect the other regions considered. In terms of largest cumulative effects across 50 weeks after a 1 s.d. shock of some region, FEIR analyses indicated that Johor, Pahang, Melaka and Pulau Pinang provide the biggest long run effects to the entire region, whereas Singapore, Johor, Melaka and Perlis received the most effects from shocks when other regions are perturbed. In terms of overall effect from receiving as well as transmitting shocks to other regions, Singapore, Johor, Melaka and Kuala Lumpur ranked the highest in terms of effects. (figure 6)

## 4. Discussion

The incidence of dengue, and thus the global burden of this particular infectious disease, has grown dramatically around the world in recent decades. For Singapore and Malaysia, a sharp increase in incidence has been observed in 2019 and 2020. Both countries are at increased risk of infectious disease spread due to their high population density, especially in Singapore, and high connectivity to other population centres in Asia and beyond, and thus would greatly benefit from the ability to conduct inference and dengue surveillance using readily available data. This can inform national public health officials to assist in controlling and combating the disease and its adverse effects once an outbreak is observed in a nearby region. The methods described in this paper provide characterization of inter-regional disease transmissions, which allows for the mentioned inference and forecasting of dengue cases transmitted across regions.

Modelling inter-regional dengue transmission dynamics of dengue is important for understanding the transmission of cases across highly connected countries and disease surveillance purposes. However, little work has been conducted on modelling higher frequency weekly data due to data unavailability and the large number of parameters required to estimate these models. While [32] has estimated multi-annual synchronous relationships between dengue in Southeast Asian nations, these low-frequency dynamic signatures are used more for long-term policy planning rather than real-time surveillance. The SSTAR methodology is able to explicitly account for *a priori* information on spatial connectivity to estimate relationships between regions in the Malay peninsula and northern Borneo on a weekly basis and account for changes in dengue dynamics across the region (figure 1). While spatio-temporal effects of other regions’ climate may be experienced locally, it can only affect dengue case count for each region through dengue case counts from neighbouring regions from the connectivity matrix parameterization. Climatic edge effects from surrounding regions may also influence vector breeding potential and thus case counts [33], and is likely to generate negligible noise on spatio-temporal inference due to the large spatial scale of the data under consideration. SSTAR is also able to disseminate and regularize medium-term dengue transmission signatures for out-of-sample forecasting (figures 3 and 4) and lastly, conduct inference on long-term external attribution of dengue cases between regions with cumulative FEIRs (figure 6). This allows for several public health applications. First, by inferring the spread of dengue across counties using SSTAR, officials may be provided with signals of a possible increase in local case counts from an outbreak in another a region. Second, by demonstrating SSTAR’s forecasting ability over one to four week ahead forecast assessment, the likely size of case counts in the near term can be estimated. This allows for vector control and other interventions to be ramped up to reduce the transmission potential.

Results from univariate testing indicated that a region’s dengue transmission counts may affect those of its neighbours. In especially connected areas such as Johor and Singapore with a daily movement of 300 000 individuals overland, the connectivity matrices were important as indicated by the GCT. The quasi-imputation study showed that even if we do not consider a region’s dengue transmission counts over long periods of time, substantial inference on the density of its dengue transmission counts may be achieved using the SSTAR approach compared to looking at climatic measurements alone. Notably, this exercise showed that the SSTAR approach with the connectivity matrix but with and without climate have similar imputation densities. As the connectivity matrix subsumes both the influence of dengue case counts from other regions and thus climatic forcing of other regions through external dengue case counts, it is likely that some of the results for imputation are driven by synchrony in climate as well as dengue case counts. Ascertaining the contributing magnitude of each factor, however, requires further data on human mobility. Bootstrapping the SSTAR also showed variable importance and significance of both a region’s past counts as well as other regions’ past counts to explain contemporaneous dengue transmission, with high levels of local dengue transmissions attributed to external dengue transmissions (figure 5 and 6). Highly connected areas such as Johor, Singapore and Kuala Lumpur showed elevated cumulative FEIR (table 2). Our results point towards long-run persistence of dengue transmission behaviour, with a one-time 1 s.d. shock in a highly connected region leading to long-run perturbations in the region even after 50 weeks (electronic supplementary material, S1). Lastly, by inducing a parsimonious structure on all 16 administrative regions’ dengue transmission counts while taking into account regional connectivity, we created a specification which surpasses the baseline STAR across regions in forecasting performance, although in absolute terms, the error rate is still noticeable relative to the weekly incidence in each region.

**Table 2.** Cumulative 50-week impulse responses.

FEIR receive		FEIR trasmit	
Singapore	0.36	Johor	0.38
Johor	0.23	Melaka	0.19
Melaka	0.19	Pulau Pinang	0.19
Kuala Lumpur	0.16	Kuala Lumpur	0.17
Perlis	0.16	Pahang	0.17
Kelantan	0.13	Singapore	0.17
Pahang	0.12	Negeri Sembilan	0.16
Perak	0.11	Selangor	0.15
Terengganu	0.11	Kedah	0.13
Negeri Sembilan	0.1	Perak	0.12
Selangor	0.1	Sarawak	0.08
Kedah	0.1	Perlis	0.07
Pulau Pinang	0.07	Kelantan	0.05
Labuan	0.06	Terengganu	0.04
Sabah	0.06	Sabah	0.02
Sarawak	0.02	Labuan	0.01

The STAR model as described has been applied to problems such as inferring and accounting for spatio-temporal variations in phenomena such as ageing, employment [34] as well as property prices [35]. It can also be applied to problems in infectious disease across regions as evidenced from this paper and inference could be conducted for recorded influenza or measles case counts across regions. Penalized estimation as proposed in SSTAR can also be used for valid inference in the same specification but take into account a much larger number of covariates and perform better out of sample, where the traditional least-squares approach fails [27].

There are, however, several limitations of the approach outlined above. STAR and SSTAR demands model parsimony as each additional region or lag leads to an increase in the number of parameters to be estimated, and estimation breaks down as soon as this exceeds the sample size. Regularized estimation of parameters also means that complete statistical inference with proper confidence intervals cannot be recovered in SSTAR, even though our approach to bootstrap approximation has valid asymptotic properties. Other spatio-temporal specific penalties could be more appropriate for the model problem such as fused lasso, but these pose even larger difficulties for statistical inference and may not be useful for forecasting. More comprehensive inference on inter-regional dengue dynamics is not possible using SSTAR due to the well-known issue of identification for the spatio-temporal class of vector-autoregressions [36]. Dengue serotypic history across regions which may inform the relative rate of infectivity between regions also cannot be account for within the model due to a lack of publicly available data of serotype composition across time. Serotype data across regions and time may be placed into separate SSTAR equations for each region, which may allow valuable inference on how genetic interactions may lead to the rise and fall of dengue cases on a larger scale. The proposed method also assumes the availability of data, which makes it more suitable for endemic infectious disease. For novel emerging diseases in the absence of data, the SSTAR cannot be calibrated well and mechanistic epidemic models taking into account spatial heterogeneity would be more appropriate [37]. Lastly, while all sub-national regions should be ideally accounted for in this study, dengue transmission data at a weekly frequency is not publicly available for many countries and future work can incorporate spatial restrictions to global–local time series models by taking into account multiple weight matrices integrating land and air transport volumes, population size and dengue incidence [38] for a more accurate characterization of dengue transmission counts worldwide.

## 5. Conclusion

To the best of the authors’ knowledge, this is the first application of STAR specifications for conducting inference on inter-regional dengue transmission dynamics on a weekly basis. We develop a sparse estimation strategy (SSTAR) to delineate interregional dengue transmission counts by explicitly accounting for geographical patterns in connectivity and adjacency. We also showed that the SSTAR is not an artefact of *a priori* knowledge on connectivity, through assessing model predictive densities even when own region counts are assumed missing. Through forecast impulse responses, we found that substantial local dengue transmissions are externally attributable and highly connected regions transmit long-run persistent shocks to other regions. Finally, the proposed penalized estimation methodology is shown to dominate inter-regional forecasting of dengue transmission counts up to four weeks ahead.

## Supplementary Material

Technical Appendix 1

## Supplementary Material

Technical Appendix 2

## Supplementary Material

Study Data

## Data accessibility

The data used to support the findings of this study are uploaded as electronic supplementary material accompanying the study.
