# Supplementary material for: Explicit characterization of human population connectivity reveals long run persistence of interregional dengue shocks
Source: J R Soc Interface. 2020 Jul 22;17(168):20200340. doi: 10.1098/rsif.2020.0340 (PMC7423435; doi:10.1098/rsif.2020.0340)
Supplement: Technical Appendix 1 [file rsif20200340supp1.pdf]

Technical Appendix 1

Contents

1 Model Specification (Sparse Space-Time Autoregressive) 1

2 SSTAR Forecast Error Impulse Response Function 2

3 SSTAR Generalised Impulse Response Function 3

4 Region Summary Statistics 4

5 Forecast Mean Absolute Error Across Regions 4

5.1 1 Week Ahead rMAE 4
5.2 2 Week Ahead rMAE 5
5.3 3 Week Ahead rMAE 5
5.4 4 Week Ahead rMAE 6
5.5 1 Week Ahead MAE 6
5.6 2 Week Ahead MAE 7
5.7 3 Week Ahead MAE 7
5.8 4 Week Ahead MAE 8

6 SSTAR Bootstrap Intervals 8

6.1 Johor 8
6.2 Kedah 10
6.3 Kelantan 12
6.4 Kuala Lumpur 14
6.5 Labuan 16
6.6 Melaka 18
6.7 Pahang 22
6.8 Perak 24
6.9 Perlis 26
6.10 Pulau Pinang 28
6.11 Sabah 30
6.12 Sarawak 32
6.13 Selangor 34
6.14 Singapore 36
6.15 Terengganu 38

7 Climate Sensitivity Analysis 39

1 Model Specification (Sparse Space-Time Autoregressive)

We consider the following Sparse Space-Time Autoregressive (SSTAR) model for R regions with the following specification (1), with the stack matrix formulation is defined here in order to derive the SSTAR specific forecast error impulse response function in the following section.

Our SSTAR consists of matrices $\mathbf{Y}_{t-i_1,(R\times 1)}$, $\mathbf{A}_{i_1,(R\times R)}$, $\mathbf{B}_{i_2,(R\times R)}$, $\mathbf{W}_{i_2,(R\times R)}$. In addition, we consider a diagonal time autoregressive matrix $\mathbf{diag}(\mathbf{A}_{i_1}) = \phi_{i_1,1:\mathrm{R}}$ with $\phi_{i_1,1:\mathrm{R}}$ entry denoting the i_1^{th} autoregressive coefficient for regions 1 to R, a diagonal time-space autoregressive matrix $\mathbf{diag}(\mathbf{B}_{i_2}) = \gamma_{i_2,1:\mathrm{R}}$ with $\gamma_{i_2,1:\mathrm{R}}$ entry denoting the i_2^{th} space-time recursive coefficient for regions 1 to R and a weight matrix with diagonal entries made to be zero $\mathbf{diag}(\mathbf{W}) = W$ the distance/adjacency matrix between regions as described in the main text. We may also consider H exogenous variables $\mathbf{C}_{i_3,u,(R\times R)}$, with

$\mathbf{diag}(\mathbf{C}_{i_3,u}) = c_{i_3,u,1:\mathrm{R}}$ denoting the i_3^{th} lag of variable u associated to Y_t. P_1, P_2, P_3 are the number of lag terms associated to autoregressive, space-time recursive and exogenous coefficients respectively. Finally ϵ_t denotes the noise vector.

$$\mathbf{Y}_t = \sum_{i_1=1}^{P_1} \mathbf{A}_{i_1}\mathbf{Y}_{t-i_1} + \sum_{i_2=1}^{P_2} \mathbf{B}_{i_2}\mathbf{W}\mathbf{Y}_{t-i_2} + \sum_{u=1}^{H}\sum_{i_3=1}^{P_3} \mathbf{C}_{i_3,u}\mathbf{X}_{u,t-i_3} + \epsilon_t \tag{1}$$

It is easy to see that SSTAR is of seemingly-unrelated regression form by considering the model specification for the kth row of Y_t for the kth region (2) , with estimation proceeding by considering the following equation by equation penalized minimization problem iteratively for all regions (3).

$$y_{t,k} = \sum_{i_1=1}^{P_1} \phi_{i_1,k} y_{t-i_1,k} + \sum_{i_2=1}^{P_2} \gamma_{i_2,k} W y_{t-i_2,-k} + \sum_{u=1}^{H}\sum_{i_3=1}^{P_3} c_{i_3,u,k} X_{u,t-i_3} + e_t \tag{2}$$

$$\underset{\phi,\gamma,c,\lambda}{\operatorname{argmin}} \left[\|y_{t,k} - \sum_{i_1=1}^{P_1} \phi_{i_1,k} y_{t-i_1,k} - \sum_{i_2=1}^{P_2} \gamma_{i_2,k} W y_{t-i_2,-k} - \sum_{u=1}^{H}\sum_{i_3=1}^{P_3} c_{i_3,u,k} X_{u,t-i_3}\|^2 - \lambda\|\phi,\gamma,c\| \right] \tag{3}$$

2 SSTAR Forecast Error Impulse Response Function

The companion form of SSTAR was utilized to derive the forecast error impulse response function (FEIR) (4). Define $\mathbf{Y}_T$ to be a Px1 vector where entries $\{\mathbf{Y}_t \dots \mathbf{Y}_{t_p-1}\}$ are NxR observations of N time-points from R regions. $\mathbf{A}_{PRxPR}$ an autoregressive matrix as defined in (6), $\mathbf{B}_{PRxPR}$ a space-time autoregressive matrix as defined in (7) and $\mathbf{W}_{PRxPR}$ a space-time diagonal weight matrix (8) with $\mathbf{diag}(\mathbf{W}) = W$, where W_{RxR} the time-invariant weight matrices defining distance between regions as described in section 1. We deprecate exogenous variables from FEIR computations as they may be treated as a time varying intercept term.

$$\mathbf{Y}_T = \mathbf{A}\mathbf{Y}_{T-1} + \mathbf{B}\mathbf{W}\mathbf{Y}_{T-1} + \varepsilon_t \tag{4}$$

$$\mathbf{Y}_T = \begin{bmatrix} \mathbf{Y}_t & \mathbf{Y}_{t-1} & \dots & \mathbf{Y}_{t-p_1-1} \end{bmatrix}' \tag{5}$$

$$\mathbf{A} = \begin{bmatrix} \mathbf{A_1} & \mathbf{A_2} & \dots & \dots & \mathbf{A}_{p_1} \\ I_n & 0 & \dots & \dots & 0 \\ 0 & I_n & \dots & \dots & \vdots \\ \vdots & & \ddots & \dots & \vdots \\ 0 & \dots & \dots & I_n & 0 \end{bmatrix} \tag{6}$$

$$\mathbf{B} = \begin{bmatrix} \mathbf{B_1} & \mathbf{B_2} & \dots & \dots & \mathbf{B}_{p_2} \\ I_n & 0 & \dots & \dots & 0 \\ 0 & I_n & \dots & \dots & \vdots \\ \vdots & & \ddots & \dots & \vdots \\ 0 & \dots & \dots & I_n & 0 \end{bmatrix} \tag{7}$$

$$\mathbf{W} = \begin{bmatrix} W & 0 & \dots & 0 \\ 0 & W & \dots & \vdots \\ \vdots & \vdots & \ddots & 0 \\ 0 & 0 & \dots & W \end{bmatrix} \tag{8}$$

By defining the FEIR for some j^{th} region shock as the effect of $\varepsilon_{t,j}$ on $\mathbf{Y}_{T+h}$ [2], we yield the FEIR for SSTAR on the hth horizon by recursion as in (9). $\mathbf{e}_j$ denotes a selection vector with zeros everywhere except $\mathbf{e}_{jj} = 1$.

$$\begin{aligned}
\frac{\partial \mathbf{Y}_{T+h}}{\partial \epsilon_{t,j}} &= \frac{\partial}{\partial \epsilon_{t,j}} \mathbf{A}\mathbf{Y}_{T+h-1} + \mathbf{B}\mathbf{W}\mathbf{Y}_{T+h-1} + \epsilon_{t+h-1} \\
&= \frac{\partial}{\partial \epsilon_{t,j}} (\mathbf{A} + \mathbf{B}\mathbf{W})\mathbf{Y}_{t+h-1} + \epsilon_{t+h-1} \\
&\dots \\
&\dots \\
&= \frac{\partial}{\partial \epsilon_{t,j}} (\mathbf{A} + \mathbf{B}\mathbf{W})^h \mathbf{Y}_T + \sum_{i=1}^{h} (\mathbf{A} + \mathbf{B}\mathbf{W})^i \epsilon_i \\
&= \sum_{i=1}^{h} (\mathbf{A} + \mathbf{B}\mathbf{W})^i \epsilon_i
\end{aligned} \tag{9}$$

As we assume no contemporaneous relationships for the estimated model, we can simply identify α level confidence intervals for the IRF by using the bootstrap interval estimates for (4). The procedure proceeds as follows:

1. Retrieve point estimates for $\hat{\mathbf{A}}, \hat{\mathbf{B}}$ by (3)
2. Obtain bootstrap confidence intervals by block bootstraping N samples and re-estimating $\hat{\mathbf{A}}_{\text{boot}}, \hat{\mathbf{B}}_{\text{boot}}$ at each iteration
3. Use samples obtained from Step 2 to construct the sampling distributions $\hat{\mathbf{P}}_{\text{A}}, \hat{\mathbf{P}}_{\text{B}}$
4. Retrieve the FEIR intervals by plugging in α and 1- α sampled values, $\hat{\mathbf{A}}_{\alpha}, \hat{\mathbf{B}}_{\alpha}$,$\hat{\mathbf{A}}_{1-\alpha}, \hat{\mathbf{B}}_{1-\alpha}$ into (9).

3 SSTAR Generalised Impulse Response Function

We modified Pesaran and Shin's (1998) [3] procedure to the SSTAR to obtain the generalised impulse response functions (GIRF). The GIRF is defined as the difference of conditional expectation of the forward forecast $\mathbf{y}_{t+j}$ given an one time shock δ_i occurs in series j given the information set Ω_{t-1}.

$$GI(j, \delta, \Omega_{t-1}) = E[\mathbf{y}_{t+j} | \epsilon_{it} = \delta_i, \Omega_{t-1}] - E[\mathbf{y}_{t+j} | \Omega_{t-1}] \tag{10}$$

The SSTAR (4) has the VMA(∞) form required for computation of GIRF after deriving the companion SSTAR form for the specification. $\mathbf{G_i X_i}$ denotes the companion form matrix of exogenous variables irrelevant to GIRF calculations.

$$\mathbf{Y}_t = \sum_{i=0}^{\infty} (\mathbf{A} + \mathbf{B}\mathbf{W})^i \epsilon_{t-i} + \sum_{i=0}^{\infty} (\mathbf{G_i X_i})^i \tag{11}$$

We detail the full computational procedure to compute GIRF for SSTAR below:

1. Obtain the error variance-covariance matrix of under L_1 penalty by adapting Fan, Guo, and Hao (2012)'s estimator [1] to the vector autoregressive case with $\hat{s}_{L,\lambda}$ number of nonzero coefficients under penalized estimation :

   $$\hat{\Sigma}_{L,\lambda} = \frac{1}{n - \hat{s}_{L,\lambda}} \sum_{t=1}^{T} \| \mathbf{Y_t} - \hat{\mathbf{A}}\mathbf{Y}_{t-1} - \hat{\mathbf{B}}\mathbf{W}\mathbf{Y}_{t-1} - \hat{\mathbf{C}}\mathbf{X}_{t-1} \|_2 \tag{12}$$

   This provides us with the approximation $\hat{\epsilon} \sim N(0, \hat{\Sigma}_{L,\lambda})$

2. Pick a one standard deviation shock for the jth region $\epsilon_{it} = \delta_i = \sigma_{jj}$
3. Modify Pesaran and Shin's (1998) expression to yield the scaled GIRF for the SSTAR, where $\mathbf{\Lambda}_{PRxPR}$ is a selection square matrix $diag(\Lambda) = 1$ for the first 1 to J diagonals and 0 everywhere else.

   $$GI(j, \delta, \Omega_{t-1}) = \sigma_{jj}^{-0.5} (\hat{\mathbf{A}} + \hat{\mathbf{B}}\mathbf{W})^j \hat{\mathbf{\Sigma}} \mathbf{e}_j \mathbf{\Lambda}_{L,\lambda} \tag{13}$$

4 Region Summary Statistics

Region	Mean	Standard Deviation
Johor	127.55	116.54
Kedah	17.69	9.37
Kelantan	79.27	153.21
Kuala Lumpur	103.59	65.25
Labuan	1.35	2.97
Melaka	30.82	22.94
Negeri Sembilan	38.73	32.37
Pahang	33.27	23.33
Perak	81.95	72.85
Perlis	3.98	3.30
Pulau Pinang	46.75	39.08
Sabah	36.04	26.67
Sarawak	36.13	30.45
Selangor	651.73	466.23
Singapore	185.76	157.96
Terengganu	21.42	23.92

Mean and standard deviation of each region's dengue case counts from 2000-2017

5 Forecast Mean Absolute Error Across Regions

5.1 1 Week Ahead rMAE

Region	$\frac{\text{MAE}_{\text{SSTAR}}}{\text{MAE}_{\text{AR}}}$	$\frac{\text{MAE}_{\text{SSTAR}}}{\text{MAE}_{\text{FGLS-AR}}}$	$\frac{\text{MAE}_{\text{SSTAR}}}{\text{MAE}_{\text{STAR}}}$	$\frac{\text{MAE}_{\text{SSTAR}}}{\text{MAE}_{\text{STAR-2}}}$
Johor	0.95	0.94	0.92	0.94
Kedah	0.93	0.94	0.89	0.89
Kelantan	0.66	0.58	0.55	0.59
Kuala Lumpur	0.95	0.92	0.91	0.91
Labuan	0.75	0.71	0.70	0.73
Melaka	0.93	0.86	0.82	0.86
Negeri Sembilan	0.90	0.84	0.80	0.84
Pahang	0.94	0.91	0.88	0.90
Perak	0.81	0.79	0.77	0.81
Perlis	0.94	0.93	0.91	0.92
Pulau Pinang	0.92	0.91	0.89	0.90
Sabah	0.97	0.95	0.94	0.95
Sarawak	0.97	0.90	0.82	0.89
Selangor	0.88	0.83	0.81	0.85
Singapore	0.85	0.79	0.75	0.80
Terengganu	0.89	0.84	0.82	0.87
Mean	0.89	0.85	0.82	0.85

SSTAR was estimated using penalized least squares and 5-fold cross validation with both connectivity and weight matrices within the specification, FGLS-AR was estimated using feasible generalized least squares with variance weighted by own region counts, STAR and STAR-2 were estimated using least squares with connectivity and weight matrices of other regions added into the specification

5.2 2 Week Ahead rMAE

Region	$\frac{\text{MAE}_{\text{SSTAR}}}{\text{MAE}_{\text{AR}}}$	$\frac{\text{MAE}_{\text{SSTAR}}}{\text{MAE}_{\text{FGLS-AR}}}$	$\frac{\text{MAE}_{\text{SSTAR}}}{\text{MAE}_{\text{STAR}}}$	$\frac{\text{MAE}_{\text{SSTAR}}}{\text{MAE}_{\text{STAR-2}}}$
Johor	0.90	0.87	0.87	0.88
Kedah	0.82	0.79	0.77	0.72
Kelantan	0.36	0.33	0.30	0.30
Kuala Lumpur	1.00	0.93	0.88	0.96
Labuan	0.50	0.39	0.38	0.42
Melaka	0.85	0.70	0.63	0.69
Negeri Sembilan	0.73	0.69	0.57	0.66
Pahang	0.80	0.78	0.72	0.73
Perak	0.80	0.64	0.63	0.70
Perlis	0.94	0.84	0.78	0.79
Pulau Pinang	0.90	0.81	0.72	0.81
Sabah	0.90	0.90	0.85	0.87
Sarawak	0.69	0.64	0.53	0.57
Selangor	0.75	0.72	0.67	0.72
Singapore	0.76	0.60	0.55	0.64
Terengganu	0.80	0.71	0.67	0.77
Mean	0.78	0.71	0.66	0.70

SSTAR was estimated using penalized least squares and 5-fold cross validation with both connectivity and weight matrices within the specification, FGLS-AR was estimated using feasible generalized least squares with variance weighted by own region counts, STAR and STAR-2 were estimated using least squares with connectivity and weight matrices of other regions added into the specification

5.3 3 Week Ahead rMAE

Region	$\frac{\text{MAE}_{\text{SSTAR}}}{\text{MAE}_{\text{AR}}}$	$\frac{\text{MAE}_{\text{SSTAR}}}{\text{MAE}_{\text{FGLS-AR}}}$	$\frac{\text{MAE}_{\text{SSTAR}}}{\text{MAE}_{\text{STAR}}}$	$\frac{\text{MAE}_{\text{SSTAR}}}{\text{MAE}_{\text{STAR-2}}}$
Johor	0.79	0.72	0.64	0.73
Kedah	0.95	0.79	0.59	0.69
Kelantan	0.30	0.25	0.25	0.27
Kuala Lumpur	0.76	0.74	0.63	0.70
Labuan	0.33	0.27	0.22	0.26
Melaka	0.71	0.62	0.53	0.63
Negeri Sembilan	0.71	0.70	0.56	0.69
Pahang	0.79	0.67	0.65	0.81
Perak	0.61	0.52	0.67	0.75
Perlis	0.77	0.57	0.53	0.76
Pulau Pinang	0.71	0.62	0.53	0.68
Sabah	0.83	0.84	0.81	0.80
Sarawak	0.70	0.60	0.50	0.58
Selangor	0.77	0.63	0.52	0.65
Singapore	0.53	0.38	0.31	0.41
Terengganu	0.72	0.57	0.54	0.66
Mean	0.69	0.59	0.53	0.63

SSTAR was estimated using penalized least squares and 5-fold cross validation with both connectivity and weight matrices within the specification, FGLS-AR was estimated using feasible generalized least squares with variance weighted by own region counts, STAR and STAR-2 were estimated using least squares with connectivity and weight matrices of other regions added into the specification

5.4 4 Week Ahead rMAE

Region	$\frac{MAE_{SSTAR}}{MAE_{AR}}$	$\frac{MAE_{SSTAR}}{MAE_{FGLS\text{-}AR}}$	$\frac{MAE_{SSTAR}}{MAE_{STAR}}$	$\frac{MAE_{SSTAR}}{MAE_{STAR\text{-}2}}$
Johor	0.77	0.66	-	0.66
Kedah	0.54	0.38	-	0.34
Kelantan	0.22	0.19	-	0.19
Kuala Lumpur	0.66	0.54	-	0.57
Labuan	0.19	0.14	-	0.12
Melaka	0.64	0.51	-	0.51
Negeri Sembilan	0.38	0.32	-	0.26
Pahang	0.64	0.56	-	0.48
Perak	0.58	0.52	-	0.51
Perlis	0.75	0.58	-	0.42
Pulau Pinang	0.66	0.50	-	0.68
Sabah	0.60	0.59	-	0.48
Sarawak	0.59	0.47	-	0.34
Selangor	0.53	0.46	-	0.49
Singapore	0.42	0.26	-	0.35
Terengganu	0.42	0.33	-	0.37
Mean	0.54	0.44	-	0.42

SSTAR was estimated using penalized least squares and 5-fold cross validation with both connectivity and weight matrices within the specification, FGLS-AR was estimated using feasible generalized least squares with variance weighted by own region counts, STAR and STAR-2 were estimated using least squares with connectivity and weight matrices of other regions added into the specification

5.5 1 Week Ahead MAE

Region	MAE_{AR}	$MAE_{FGLS\text{-}AR}$	MAE_{SSTAR}	MAE_{STAR}	$MAE_{STAR\text{-}2}$
Johor	15.81	15.95	14.96	16.25	15.98
Kedah	3.89	3.84	3.63	4.06	4.07
Kelantan	13.65	15.62	9.00	16.32	15.30
Kuala Lumpur	13.50	13.95	12.81	14.03	14.08
Labuan	0.70	0.74	0.52	0.75	0.72
Melaka	5.62	6.12	5.24	6.38	6.09
Negeri Sembilan	9.73	10.35	8.71	10.82	10.34
Pahang	6.49	6.67	6.08	6.90	6.77
Perak	10.63	10.89	8.60	11.11	10.64
Perlis	1.48	1.51	1.40	1.53	1.52
Pulau Pinang	7.48	7.60	6.89	7.72	7.68
Sabah	6.04	6.13	5.85	6.24	6.14
Sarawak	3.71	3.99	3.61	4.38	4.07
Selangor	49.09	51.98	43.29	53.32	50.75
Singapore	14.04	15.05	11.96	15.92	14.96
Terengganu	3.81	4.01	3.38	4.13	3.90

SSTAR was estimated using penalized least squares and 5-fold cross validation with both connectivity and weight matrices within the specification, FGLS-AR was estimated using feasible generalized least squares with variance weighted by own region counts, STAR and STAR-2 were estimated using least squares with connectivity and weight matrices of other regions added into the specification

5.6 2 Week Ahead MAE

Region	MAE_{AR}	$MAE_{FGLS-AR}$	MAE_{SSTAR}	MAE_{STAR}	MAE_{STAR-2}
Johor	25.81	26.86	23.34	26.98	26.62
Kedah	5.52	5.70	4.52	5.85	6.27
Kelantan	35.58	38.48	12.63	41.84	41.82
Kuala Lumpur	15.55	16.80	15.55	17.77	16.25
Labuan	0.65	0.83	0.33	0.86	0.77
Melaka	6.60	8.05	5.61	8.95	8.18
Negeri Sembilan	10.05	10.70	7.35	12.83	11.15
Pahang	8.63	8.92	6.93	9.62	9.44
Perak	12.14	15.15	9.68	15.30	13.85
Perlis	1.70	1.91	1.60	2.04	2.02
Pulau Pinang	8.47	9.35	7.61	10.63	9.34
Sabah	7.51	7.46	6.73	7.94	7.74
Sarawak	5.71	6.19	3.95	7.48	6.92
Selangor	76.71	79.86	57.82	86.93	80.26
Singapore	23.08	29.46	17.64	31.93	27.44
Terengganu	5.86	6.59	4.70	6.98	6.11

SSTAR was estimated using penalized least squares and 5-fold cross validation with both connectivity and weight matrices within the specification, FGLS-AR was estimated using feasible generalized least squares with variance weighted by own region counts, STAR and STAR-2 were estimated using least squares with connectivity and weight matrices of other regions added into the specification

5.7 3 Week Ahead MAE

Region	MAE_{AR}	$MAE_{FGLS-AR}$	MAE_{SSTAR}	MAE_{STAR}	MAE_{STAR-2}
Johor	31.56	34.25	24.81	38.71	34.14
Kedah	4.42	5.26	4.18	7.10	6.09
Kelantan	51.23	63.18	15.58	63.01	58.64
Kuala Lumpur	26.08	27.06	19.95	31.50	28.57
Labuan	1.22	1.52	0.41	1.85	1.56
Melaka	9.44	10.75	6.71	12.67	10.68
Negeri Sembilan	13.09	13.15	9.25	16.44	13.41
Pahang	9.57	11.24	7.52	11.55	9.26
Perak	20.83	24.38	12.62	18.79	16.81
Perlis	1.75	2.33	1.34	2.51	1.76
Pulau Pinang	13.55	15.50	9.57	18.03	14.17
Sabah	10.04	9.90	8.34	10.29	10.41
Sarawak	7.54	8.72	5.27	10.64	9.09
Selangor	94.13	114.27	72.03	139.60	110.99
Singapore	33.92	46.78	17.99	57.16	44.38
Terengganu	7.97	10.05	5.77	10.65	8.77

SSTAR was estimated using penalized least squares and 5-fold cross validation with both connectivity and weight matrices within the specification, FGLS-AR was estimated using feasible generalized least squares with variance weighted by own region counts, STAR and STAR-2 were estimated using least squares with connectivity and weight matrices of other regions added into the specification

5.8 4 Week Ahead MAE

Region	MAE_{AR}	$MAE_{FGLS\text{-}AR}$	MAE_{SSTAR}	MAE_{STAR}	$MAE_{STAR\text{-}2}$
Johor	39.77	46.79	30.65	-	46.66
Kedah	7.88	11.14	4.26	-	12.41
Kelantan	95.48	109.24	20.90	-	110.20
Kuala Lumpur	30.20	36.38	19.83	-	34.74
Labuan	1.72	2.36	0.32	-	2.71
Melaka	11.93	15.02	7.65	-	14.90
Negeri Sembilan	24.45	29.34	9.27	-	35.82
Pahang	11.49	13.21	7.35	-	15.28
Perak	26.16	29.03	15.06	-	29.45
Perlis	2.28	2.94	1.70	-	4.02
Pulau Pinang	18.81	24.72	12.46	-	18.29
Sabah	16.36	16.47	9.76	-	20.25
Sarawak	8.62	10.77	5.10	-	15.11
Selangor	122.06	138.22	64.17	-	130.77
Singapore	70.77	115.04	30.07	-	85.91
Terengganu	18.63	24.11	7.85	-	21.41

SSTAR was estimated using penalized least squares and 5-fold cross validation with both connectivity and weight matrices within the specification, FGLS-AR was estimated using feasible generalized least squares with variance weighted by own region counts, STAR and STAR-2 were estimated using least squares with connectivity and weight matrices of other regions added into the specification

6 SSTAR Bootstrap Intervals

6.1 Johor

	2.5 Quantile	97.5 Quantile	Mean
Own Counts Lag 1	0.09	0.71	0.45
Own Counts Lag 2	0.00	0.53	0.24
Own Counts Lag 3	-0.09	0.30	0.05
Own Counts Lag 4	-0.08	0.22	0.02
Own Counts Lag 5	-0.18	0.21	0.01
Own Counts Lag 6	-0.05	0.24	0.03
Own Counts Lag 7	-0.20	0.22	0.01
Own Counts Lag 8	-0.03	0.32	0.05
Own Counts Lag 9	-0.17	0.18	0.00
Own Counts Lag 10	-0.19	0.11	-0.01
Absolute Humidity Lag 1	-0.06	29.66	2.72
Absolute Humidity Lag 2	-9.16	24.17	1.27
Absolute Humidity Lag 3	-42.70	26.73	-2.11
Absolute Humidity Lag 4	-10.06	54.63	7.00
Absolute Humidity Lag 5	0.00	60.38	10.02
Absolute Humidity Lag 6	-77.20	0.00	-16.52
Absolute Humidity Lag 7	-52.41	0.00	-5.44
Absolute Humidity Lag 8	-29.12	25.75	-0.75
Absolute Humidity Lag 9	0.00	50.75	7.39
Absolute Humidity Lag 10	-3.05	23.16	1.53
Relative Humidity Lag 1	0.00	84.14	21.31
Relative Humidity Lag 2	0.00	84.42	17.60
Relative Humidity Lag 3	-32.93	14.08	-2.04
Relative Humidity Lag 4	-28.98	8.94	-1.90
Relative Humidity Lag 5	-34.18	7.09	-2.49
Relative Humidity Lag 6	-14.10	5.97	-0.48
Relative Humidity Lag 7	-11.42	21.15	1.42
Relative Humidity Lag 8	-8.35	33.03	3.33
Relative Humidity Lag 9	-16.92	20.08	0.20
Relative Humidity Lag 10	0.00	37.65	5.87
Total Precipitation Lag 1	-151.25	0.00	-45.64

Total Precipitation Lag 2	-121.00	0.00	-24.38
Total Precipitation Lag 3	-80.96	16.48	-13.40
Total Precipitation Lag 4	-79.79	9.39	-16.76
Total Precipitation Lag 5	-17.93	108.12	14.60
Total Precipitation Lag 6	-71.65	23.27	-5.12
Total Precipitation Lag 7	-57.79	40.69	-2.25
Total Precipitation Lag 8	-47.03	33.79	-3.03
Total Precipitation Lag 9	-74.40	34.28	-10.89
Total Precipitation Lag 10	-76.96	5.20	-15.59
Temperature Lag 1	-39.55	0.00	-4.86
Temperature Lag 2	-53.40	0.00	-6.07
Temperature Lag 3	-20.59	7.98	-0.75
Temperature Lag 4	0.00	41.37	5.47
Temperature Lag 5	0.00	73.52	12.43
Temperature Lag 6	-53.87	0.00	-10.24
Temperature Lag 7	-44.58	0.00	-6.33
Temperature Lag 8	-31.82	2.46	-2.95
Temperature Lag 9	0.00	49.68	7.07
Temperature Lag 10	-34.31	9.37	-2.75
Connectivity Lag 1	0.00	0.31	0.07
Connectivity Lag 2	0.00	0.30	0.05
Connectivity Lag 3	-0.03	0.05	-0.00
Connectivity Lag 4	0.00	0.19	0.01
Connectivity Lag 5	0.00	0.10	0.00
Connectivity Lag 6	0.00	0.21	0.02
Connectivity Lag 7	0.00	0.43	0.05
Connectivity Lag 8	0.00	0.02	0.00
Connectivity Lag 9	0.00	0.43	0.09
Connectivity Lag 10	0.00	0.35	0.05
Adjacency Lag 1	-0.13	0.00	-0.01
Adjacency Lag 2	-0.34	0.00	-0.03
Adjacency Lag 3	-0.54	0.00	-0.07
Adjacency Lag 4	-0.59	0.00	-0.07
Adjacency Lag 5	-0.63	0.00	-0.10
Adjacency Lag 6	-0.44	0.00	-0.05
Adjacency Lag 7	-0.94	0.00	-0.12
Adjacency Lag 8	-0.98	0.00	-0.21
Adjacency Lag 9	0.00	0.00	0.00
Adjacency Lag 10	-0.32	0.00	-0.03

Table 1: SSTAR Bootstrap Values: Johor

6.2 Kedah

	2.5 Quantile	97.5 Quantile	Mean
Own Counts Lag 1	-0.03	0.31	0.09
Own Counts Lag 2	-0.31	0.00	-0.09
Own Counts Lag 3	0.00	0.42	0.11
Own Counts Lag 4	-0.06	0.30	0.06
Own Counts Lag 5	-0.13	0.19	0.01
Own Counts Lag 6	-0.30	0.08	-0.03
Own Counts Lag 7	0.00	0.49	0.21
Own Counts Lag 8	-0.02	0.34	0.08
Own Counts Lag 9	-0.28	0.11	-0.04
Own Counts Lag 10	-0.37	0.01	-0.06
Absolute Humidity Lag 1	-8.19	5.16	-0.08
Absolute Humidity Lag 2	-9.92	9.30	0.01
Absolute Humidity Lag 3	-1.63	15.56	1.71
Absolute Humidity Lag 4	-4.10	10.50	0.86
Absolute Humidity Lag 5	-0.01	16.02	1.34
Absolute Humidity Lag 6	-16.15	0.00	-1.53
Absolute Humidity Lag 7	-1.45	19.43	1.48
Absolute Humidity Lag 8	-19.38	0.00	-4.23
Absolute Humidity Lag 9	-3.59	10.32	0.57
Absolute Humidity Lag 10	-15.08	2.82	-1.82
Relative Humidity Lag 1	0.00	8.69	0.72
Relative Humidity Lag 2	-0.86	8.97	1.06
Relative Humidity Lag 3	-4.86	3.34	-0.12
Relative Humidity Lag 4	-8.94	4.35	-0.25
Relative Humidity Lag 5	0.00	12.40	1.98
Relative Humidity Lag 6	0.00	2.52	0.14
Relative Humidity Lag 7	0.00	7.10	0.51
Relative Humidity Lag 8	-8.73	0.00	-0.62
Relative Humidity Lag 9	-2.80	6.14	0.18
Relative Humidity Lag 10	-8.74	0.00	-0.96
Total Precipitation Lag 1	-12.93	0.66	-2.63
Total Precipitation Lag 2	-8.01	4.90	-0.40
Total Precipitation Lag 3	-6.22	4.44	-0.17
Total Precipitation Lag 4	-5.07	6.45	0.38
Total Precipitation Lag 5	-3.72	7.82	0.91
Total Precipitation Lag 6	-11.46	3.22	-1.12
Total Precipitation Lag 7	-8.01	4.23	-0.61
Total Precipitation Lag 8	-1.82	10.45	1.80
Total Precipitation Lag 9	-3.45	9.91	0.81
Total Precipitation Lag 10	-9.07	1.86	-1.26
Temperature Lag 1	-15.04	0.00	-1.77
Temperature Lag 2	-10.34	0.00	-1.11
Temperature Lag 3	-0.82	9.09	0.49
Temperature Lag 4	-5.36	8.50	0.21
Temperature Lag 5	-6.67	1.87	-0.40
Temperature Lag 6	-14.94	0.00	-2.32
Temperature Lag 7	-2.13	8.16	0.53
Temperature Lag 8	-4.99	0.00	-0.29
Temperature Lag 9	-2.92	9.69	0.69
Temperature Lag 10	-0.98	8.12	0.60
Connectivity Lag 1	0.00	0.35	0.06
Connectivity Lag 2	-0.49	0.00	-0.04
Connectivity Lag 3	-0.02	0.15	0.01
Connectivity Lag 4	0.00	0.26	0.04
Connectivity Lag 5	0.00	0.29	0.03
Connectivity Lag 6	0.00	0.14	0.01
Connectivity Lag 7	0.00	0.37	0.06
Connectivity Lag 8	0.00	0.16	0.02
Connectivity Lag 9	-0.41	0.00	-0.04
Connectivity Lag 10	0.00	0.37	0.05
Adjacency Lag 1	-0.43	0.02	-0.06
Adjacency Lag 2	-0.08	0.46	0.04

Adjacency Lag 3	-0.22	0.14	-0.01
Adjacency Lag 4	-0.43	0.08	-0.05
Adjacency Lag 5	-0.57	0.00	-0.17
Adjacency Lag 6	-0.26	0.30	-0.00
Adjacency Lag 7	-0.63	0.00	-0.10
Adjacency Lag 8	-0.19	0.35	0.01
Adjacency Lag 9	-0.41	0.09	-0.08
Adjacency Lag 10	-0.44	0.04	-0.06

Table 2: SSTAR Bootstrap Values: Kedah

6.3 Kelantan

	2.5 Quantile	97.5 Quantile	Mean
Own Counts Lag 1	0.38	1.25	0.83
Own Counts Lag 2	-0.15	0.24	0.01
Own Counts Lag 3	0.00	0.38	0.05
Own Counts Lag 4	-0.07	0.54	0.05
Own Counts Lag 5	-0.16	0.55	0.03
Own Counts Lag 6	-0.45	0.00	-0.04
Own Counts Lag 7	-0.51	0.00	-0.07
Own Counts Lag 8	0.00	0.00	0.00
Own Counts Lag 9	0.00	0.15	0.01
Own Counts Lag 10	-0.03	0.03	0.00
Absolute Humidity Lag 1	-17.95	4.63	-1.01
Absolute Humidity Lag 2	-49.28	0.00	-7.87
Absolute Humidity Lag 3	-35.89	3.32	-4.34
Absolute Humidity Lag 4	-40.09	0.00	-5.83
Absolute Humidity Lag 5	-20.96	14.57	-0.34
Absolute Humidity Lag 6	-21.65	8.54	-1.09
Absolute Humidity Lag 7	-3.89	17.93	0.86
Absolute Humidity Lag 8	0.00	41.65	5.79
Absolute Humidity Lag 9	-13.05	13.06	-0.11
Absolute Humidity Lag 10	0.00	42.65	8.33
Relative Humidity Lag 1	-46.71	0.00	-7.06
Relative Humidity Lag 2	-10.01	0.00	-0.30
Relative Humidity Lag 3	-2.51	5.87	0.19
Relative Humidity Lag 4	-23.93	0.00	-2.90
Relative Humidity Lag 5	-11.41	11.76	0.08
Relative Humidity Lag 6	-18.33	3.20	-0.93
Relative Humidity Lag 7	0.00	30.88	4.02
Relative Humidity Lag 8	0.00	31.81	3.16
Relative Humidity Lag 9	-27.36	5.98	-2.82
Relative Humidity Lag 10	-16.01	1.57	-1.12
Total Precipitation Lag 1	-21.95	27.78	-1.39
Total Precipitation Lag 2	-16.81	18.38	-0.27
Total Precipitation Lag 3	-24.04	11.22	-2.44
Total Precipitation Lag 4	-21.36	27.11	0.18
Total Precipitation Lag 5	-11.38	24.60	1.66
Total Precipitation Lag 6	-26.76	13.09	-2.63
Total Precipitation Lag 7	-15.73	36.71	3.93
Total Precipitation Lag 8	-40.43	19.20	-3.07
Total Precipitation Lag 9	-24.33	18.85	-0.33
Total Precipitation Lag 10	-35.39	19.21	-1.81
Temperature Lag 1	0.00	34.11	2.10
Temperature Lag 2	-24.91	0.00	-1.61
Temperature Lag 3	-23.10	0.00	-2.17
Temperature Lag 4	-3.36	7.86	0.11
Temperature Lag 5	-11.82	9.56	-0.13
Temperature Lag 6	-15.08	19.21	0.68
Temperature Lag 7	-38.09	0.00	-3.45
Temperature Lag 8	-3.85	9.16	0.26
Temperature Lag 9	-0.60	37.68	4.00
Temperature Lag 10	0.00	57.10	13.03
Connectivity Lag 1	0.00	0.81	0.21
Connectivity Lag 2	0.00	0.56	0.05
Connectivity Lag 3	-0.05	0.60	0.05
Connectivity Lag 4	-0.00	0.60	0.06
Connectivity Lag 5	0.00	0.70	0.16
Connectivity Lag 6	0.00	0.49	0.04
Connectivity Lag 7	-0.20	0.22	0.00
Connectivity Lag 8	-0.47	0.06	-0.02
Connectivity Lag 9	0.00	0.41	0.03
Connectivity Lag 10	-0.53	0.00	-0.04
Adjacency Lag 1	-0.18	2.14	0.18
Adjacency Lag 2	-1.96	0.00	-0.29

Adjacency Lag 3	-2.55	0.00	-0.53
Adjacency Lag 4	-1.34	0.95	-0.11
Adjacency Lag 5	-0.89	0.92	-0.03
Adjacency Lag 6	-0.66	0.55	-0.02
Adjacency Lag 7	-0.47	1.06	0.06
Adjacency Lag 8	-1.31	0.32	-0.14
Adjacency Lag 9	-0.23	0.87	0.05
Adjacency Lag 10	-0.28	0.58	0.02

Table 3: SSTAR Bootstrap Values: Kelantan

6.4 Kuala Lumpur

	2.5 Quantile	97.5 Quantile	Mean
Own Counts Lag 1	0.17	0.62	0.42
Own Counts Lag 2	-0.02	0.24	0.03
Own Counts Lag 3	0.00	0.18	0.03
Own Counts Lag 4	-0.27	0.00	-0.02
Own Counts Lag 5	0.00	0.30	0.08
Own Counts Lag 6	0.00	0.32	0.08
Own Counts Lag 7	-0.14	0.03	-0.01
Own Counts Lag 8	-0.13	0.03	-0.01
Own Counts Lag 9	-0.16	0.05	-0.01
Own Counts Lag 10	-0.12	0.01	-0.01
Absolute Humidity Lag 1	-0.81	15.37	0.84
Absolute Humidity Lag 2	-25.83	2.88	-2.55
Absolute Humidity Lag 3	-19.77	0.79	-1.54
Absolute Humidity Lag 4	-29.54	0.00	-3.46
Absolute Humidity Lag 5	0.00	56.30	15.45
Absolute Humidity Lag 6	-12.74	8.64	-0.10
Absolute Humidity Lag 7	-10.16	18.09	0.73
Absolute Humidity Lag 8	-10.02	12.31	0.18
Absolute Humidity Lag 9	-31.93	0.00	-4.40
Absolute Humidity Lag 10	-12.47	8.09	-0.64
Relative Humidity Lag 1	-32.10	0.00	-5.15
Relative Humidity Lag 2	-17.27	0.00	-1.44
Relative Humidity Lag 3	0.00	20.90	1.54
Relative Humidity Lag 4	-20.88	0.00	-2.46
Relative Humidity Lag 5	0.00	22.44	2.42
Relative Humidity Lag 6	-13.26	8.86	-0.37
Relative Humidity Lag 7	-3.26	17.41	1.48
Relative Humidity Lag 8	-21.49	2.19	-1.39
Relative Humidity Lag 9	-3.52	8.23	0.25
Relative Humidity Lag 10	-21.96	0.00	-3.23
Total Precipitation Lag 1	-18.53	7.81	-2.52
Total Precipitation Lag 2	-8.74	19.36	1.77
Total Precipitation Lag 3	-13.22	17.87	1.81
Total Precipitation Lag 4	-16.53	3.09	-2.04
Total Precipitation Lag 5	-11.38	11.13	0.29
Total Precipitation Lag 6	-2.78	12.29	1.31
Total Precipitation Lag 7	-3.42	20.45	4.48
Total Precipitation Lag 8	-13.07	11.03	-0.79
Total Precipitation Lag 9	-17.86	4.51	-2.75
Total Precipitation Lag 10	-14.76	2.34	-2.38
Temperature Lag 1	0.00	26.85	5.73
Temperature Lag 2	-12.12	8.16	-0.11
Temperature Lag 3	-32.37	0.00	-6.36
Temperature Lag 4	-9.83	8.91	-0.14
Temperature Lag 5	0.00	14.94	1.17
Temperature Lag 6	-12.28	13.90	0.35
Temperature Lag 7	-12.88	9.26	-0.57
Temperature Lag 8	-3.81	18.66	1.34
Temperature Lag 9	-31.66	0.00	-5.20
Temperature Lag 10	-7.12	13.40	0.54
Connectivity Lag 1	0.00	0.27	0.10
Connectivity Lag 2	0.00	0.10	0.01
Connectivity Lag 3	0.00	0.16	0.02
Connectivity Lag 4	0.00	0.11	0.01
Connectivity Lag 5	0.00	0.06	0.00
Connectivity Lag 6	0.00	0.11	0.01
Connectivity Lag 7	-0.10	0.00	-0.01
Connectivity Lag 8	0.00	0.09	0.01
Connectivity Lag 9	0.00	0.05	0.00
Connectivity Lag 10	-0.04	0.00	-0.00
Adjacency Lag 1	0.00	0.18	0.02
Adjacency Lag 2	-0.17	0.00	-0.01

Adjacency Lag 3	-0.01	0.00	-0.00
Adjacency Lag 4	-0.05	0.01	-0.00
Adjacency Lag 5	-0.01	0.06	0.00
Adjacency Lag 6	-0.07	0.00	-0.00
Adjacency Lag 7	-0.18	0.00	-0.02
Adjacency Lag 8	-0.02	0.03	0.00
Adjacency Lag 9	-0.03	0.00	-0.00
Adjacency Lag 10	-0.02	0.05	0.00

Table 4: SSTAR Bootstrap Values: Kuala Lumpur

6.5 Labuan

	2.5 Quantile	97.5 Quantile	Mean
Own Counts Lag 1	0.29	0.98	0.65
Own Counts Lag 2	-0.37	0.13	-0.04
Own Counts Lag 3	-0.10	0.26	0.03
Own Counts Lag 4	-0.08	0.25	0.03
Own Counts Lag 5	-0.25	0.18	0.01
Own Counts Lag 6	-0.27	0.18	-0.01
Own Counts Lag 7	-0.16	0.30	0.01
Own Counts Lag 8	-0.48	0.00	-0.09
Own Counts Lag 9	-0.06	0.23	0.02
Own Counts Lag 10	-0.11	0.12	0.00
Absolute Humidity Lag 1	-1.28	3.10	0.13
Absolute Humidity Lag 2	0.00	3.30	0.40
Absolute Humidity Lag 3	-7.62	0.00	-1.38
Absolute Humidity Lag 4	-4.86	0.76	-0.51
Absolute Humidity Lag 5	-4.42	0.00	-0.62
Absolute Humidity Lag 6	-1.54	3.87	0.31
Absolute Humidity Lag 7	-3.48	2.54	-0.06
Absolute Humidity Lag 8	-1.38	4.00	0.46
Absolute Humidity Lag 9	-7.67	0.00	-1.17
Absolute Humidity Lag 10	0.00	4.38	0.48
Relative Humidity Lag 1	-1.13	4.74	0.69
Relative Humidity Lag 2	0.00	6.28	1.13
Relative Humidity Lag 3	-1.38	0.75	-0.04
Relative Humidity Lag 4	-3.20	1.54	-0.20
Relative Humidity Lag 5	-7.09	0.00	-1.39
Relative Humidity Lag 6	-1.01	3.62	0.25
Relative Humidity Lag 7	-3.37	3.81	0.06
Relative Humidity Lag 8	-5.59	1.93	-0.46
Relative Humidity Lag 9	-3.66	0.12	-0.34
Relative Humidity Lag 10	-2.16	0.96	-0.08
Total Precipitation Lag 1	-6.29	1.72	-0.84
Total Precipitation Lag 2	-8.00	0.00	-1.66
Total Precipitation Lag 3	-12.84	0.00	-2.89
Total Precipitation Lag 4	-8.34	0.46	-1.38
Total Precipitation Lag 5	-3.10	4.16	0.32
Total Precipitation Lag 6	-6.96	0.00	-2.07
Total Precipitation Lag 7	0.00	6.34	1.12
Total Precipitation Lag 8	-3.61	4.41	0.18
Total Precipitation Lag 9	-5.55	1.36	-0.49
Total Precipitation Lag 10	-7.30	0.00	-1.63
Temperature Lag 1	-2.74	1.04	-0.17
Temperature Lag 2	-2.74	0.00	-0.13
Temperature Lag 3	-7.93	0.00	-0.99
Temperature Lag 4	-3.16	1.19	-0.16
Temperature Lag 5	0.00	3.97	0.23
Temperature Lag 6	-2.89	3.22	0.09
Temperature Lag 7	-2.69	1.79	-0.10
Temperature Lag 8	0.00	5.69	0.73
Temperature Lag 9	-3.03	0.30	-0.19
Temperature Lag 10	0.00	4.69	0.55
Connectivity Lag 1	-0.03	0.02	-0.00
Connectivity Lag 2	-0.11	0.00	-0.02
Connectivity Lag 3	-0.04	0.00	-0.00
Connectivity Lag 4	-0.14	0.00	-0.04
Connectivity Lag 5	0.00	0.05	0.00
Connectivity Lag 6	0.00	0.08	0.01
Connectivity Lag 7	-0.08	0.00	-0.01
Connectivity Lag 8	-0.04	0.00	-0.00
Connectivity Lag 9	-0.04	0.00	-0.00
Connectivity Lag 10	-0.19	0.00	-0.03
Adjacency Lag 1	-0.05	0.00	-0.00
Adjacency Lag 2	-0.06	0.00	-0.00

Adjacency Lag 3	0.00	0.02	0.00
Adjacency Lag 4	0.00	0.00	-0.00
Adjacency Lag 5	0.00	0.11	0.01
Adjacency Lag 6	0.00	0.00	0.00
Adjacency Lag 7	0.00	0.01	0.00
Adjacency Lag 8	0.00	0.01	0.00
Adjacency Lag 9	0.00	0.07	0.01
Adjacency Lag 10	-0.03	0.00	-0.00

Table 5: SSTAR Bootstrap Values: Labuan

6.6 Melaka

	2.5 Quantile	97.5 Quantile	Mean
Own Counts Lag 1	0.18	0.99	0.68
Own Counts Lag 2	-0.21	0.42	0.03
Own Counts Lag 3	-0.29	0.25	-0.02
Own Counts Lag 4	-0.43	0.01	-0.08
Own Counts Lag 5	0.00	0.46	0.13
Own Counts Lag 6	0.00	0.51	0.11
Own Counts Lag 7	-0.25	0.15	-0.01
Own Counts Lag 8	-0.11	0.14	0.00
Own Counts Lag 9	-0.18	0.05	-0.01
Own Counts Lag 10	-0.20	0.01	-0.03
Absolute Humidity Lag 1	-11.29	15.06	0.38
Absolute Humidity Lag 2	-17.13	5.12	-1.32
Absolute Humidity Lag 3	-24.23	5.06	-2.90
Absolute Humidity Lag 4	-15.13	5.71	-0.99
Absolute Humidity Lag 5	-22.53	0.00	-2.69
Absolute Humidity Lag 6	-22.91	0.00	-2.39
Absolute Humidity Lag 7	0.00	40.08	5.80
Absolute Humidity Lag 8	0.00	44.95	8.61
Absolute Humidity Lag 9	-8.27	15.14	0.50
Absolute Humidity Lag 10	-4.35	4.74	-0.34
Relative Humidity Lag 1	-8.21	15.55	0.39
Relative Humidity Lag 2	-18.42	2.34	-2.58
Relative Humidity Lag 3	-28.28	0.00	-5.44
Relative Humidity Lag 4	0.00	12.71	1.25
Relative Humidity Lag 5	-2.96	8.21	0.35
Relative Humidity Lag 6	-6.04	4.18	-0.16
Relative Humidity Lag 7	0.00	21.51	4.65
Relative Humidity Lag 8	-7.71	0.00	-0.47
Relative Humidity Lag 9	-9.29	5.13	-0.24
Relative Humidity Lag 10	-9.28	9.27	0.07
Total Precipitation Lag 1	-29.12	4.14	-4.65
Total Precipitation Lag 2	-16.25	13.45	-0.95
Total Precipitation Lag 3	-23.74	17.05	-0.94
Total Precipitation Lag 4	-39.37	1.51	-8.14
Total Precipitation Lag 5	-28.76	7.72	-5.95
Total Precipitation Lag 6	-21.01	10.49	-1.80
Total Precipitation Lag 7	-33.26	8.83	-5.69
Total Precipitation Lag 8	-34.75	22.64	-3.24
Total Precipitation Lag 9	-26.58	6.26	-4.74
Total Precipitation Lag 10	-33.25	0.00	-8.65
Temperature Lag 1	-5.04	3.04	-0.12
Temperature Lag 2	-4.45	3.62	-0.08
Temperature Lag 3	-0.14	7.37	0.43
Temperature Lag 4	-18.27	0.00	-2.83
Temperature Lag 5	-18.39	0.00	-2.69
Temperature Lag 6	-12.67	0.00	-1.37
Temperature Lag 7	-8.44	1.62	-0.67
Temperature Lag 8	0.00	29.58	9.55
Temperature Lag 9	-12.92	16.97	1.12
Temperature Lag 10	-10.17	9.49	-0.05
Connectivity Lag 1	-0.06	0.16	0.01
Connectivity Lag 2	0.00	0.03	0.00
Connectivity Lag 3	0.00	0.21	0.03
Connectivity Lag 4	0.00	0.13	0.01
Connectivity Lag 5	0.00	0.31	0.04
Connectivity Lag 6	0.00	0.09	0.01
Connectivity Lag 7	-0.00	0.08	0.00
Connectivity Lag 8	0.00	0.14	0.01
Connectivity Lag 9	0.00	0.07	0.00
Connectivity Lag 10	-0.13	0.00	-0.01
Adjacency Lag 1	-0.07	0.01	-0.00
Adjacency Lag 2	-0.14	0.00	-0.02

Adjacency Lag 3	-0.01	0.02	-0.00
Adjacency Lag 4	-0.00	0.05	0.00
Adjacency Lag 5	0.00	0.16	0.02
Adjacency Lag 6	-0.01	0.03	0.00
Adjacency Lag 7	-0.07	0.00	-0.00
Adjacency Lag 8	-0.04	0.00	-0.00
Adjacency Lag 9	-0.03	0.00	-0.00
Adjacency Lag 10	-0.05	0.02	-0.00

Table 6: SSTAR Bootstrap Values: Melaka

	2.5 Quantile	97.5 Quantile	Mean
Own Counts Lag 1	0.25	0.75	0.50
Own Counts Lag 2	-0.22	0.28	0.03
Own Counts Lag 3	0.00	0.45	0.15
Own Counts Lag 4	-0.06	0.29	0.05
Own Counts Lag 5	-0.05	0.23	0.03
Own Counts Lag 6	-0.26	0.06	-0.03
Own Counts Lag 7	-0.20	0.01	-0.03
Own Counts Lag 8	-0.18	0.03	-0.02
Own Counts Lag 9	0.00	0.22	0.04
Own Counts Lag 10	-0.07	0.13	0.02
Absolute Humidity Lag 1	0.00	28.09	9.37
Absolute Humidity Lag 2	-8.89	13.26	0.76
Absolute Humidity Lag 3	0.00	24.64	4.90
Absolute Humidity Lag 4	-15.87	1.90	-1.39
Absolute Humidity Lag 5	-5.45	12.01	0.62
Absolute Humidity Lag 6	-19.26	1.81	-2.18
Absolute Humidity Lag 7	0.00	22.78	2.64
Absolute Humidity Lag 8	-15.55	3.01	-1.26
Absolute Humidity Lag 9	-3.28	13.79	0.71
Absolute Humidity Lag 10	-19.77	0.00	-5.36
Relative Humidity Lag 1	0.00	8.47	0.51
Relative Humidity Lag 2	-2.80	14.20	1.17
Relative Humidity Lag 3	-0.87	11.76	0.80
Relative Humidity Lag 4	-3.59	11.95	0.77
Relative Humidity Lag 5	-11.70	2.94	-0.73
Relative Humidity Lag 6	-24.22	1.30	-3.43
Relative Humidity Lag 7	0.00	27.87	6.84
Relative Humidity Lag 8	-11.96	11.34	0.16
Relative Humidity Lag 9	-4.56	22.75	2.11
Relative Humidity Lag 10	-7.24	5.01	-0.16
Total Precipitation Lag 1	-13.25	6.49	-1.64
Total Precipitation Lag 2	-13.37	4.57	-1.53
Total Precipitation Lag 3	-8.18	13.39	1.42
Total Precipitation Lag 4	-13.56	6.37	-1.16
Total Precipitation Lag 5	-13.58	5.13	-2.13
Total Precipitation Lag 6	-5.38	13.27	1.34
Total Precipitation Lag 7	-7.72	16.92	2.62
Total Precipitation Lag 8	-5.29	18.86	3.92
Total Precipitation Lag 9	-17.63	2.82	-2.73
Total Precipitation Lag 10	0.00	26.84	8.70
Temperature Lag 1	0.00	16.62	3.50
Temperature Lag 2	-9.49	11.19	0.42
Temperature Lag 3	0.00	15.54	1.84
Temperature Lag 4	-12.36	2.30	-0.98
Temperature Lag 5	-1.56	18.35	1.79
Temperature Lag 6	-4.55	11.17	0.65
Temperature Lag 7	-16.17	2.00	-1.27
Temperature Lag 8	-13.16	8.99	-0.59
Temperature Lag 9	-11.67	10.92	-0.24
Temperature Lag 10	-17.60	0.00	-4.77
Connectivity Lag 1	-0.03	0.01	-0.00
Connectivity Lag 2	0.00	0.06	0.01
Connectivity Lag 3	0.00	0.08	0.01
Connectivity Lag 4	0.00	0.05	0.00
Connectivity Lag 5	-0.03	0.00	-0.00
Connectivity Lag 6	0.00	0.12	0.01
Connectivity Lag 7	0.00	0.08	0.01
Connectivity Lag 8	0.00	0.19	0.04
Connectivity Lag 9	0.00	0.25	0.04
Connectivity Lag 10	0.00	0.01	0.00
Adjacency Lag 1	-0.07	0.01	-0.00
Adjacency Lag 2	0.00	0.05	0.00
Adjacency Lag 3	-0.01	0.00	-0.00
Adjacency Lag 4	-0.06	0.00	-0.01

Adjacency Lag 5	-0.08	0.00	-0.01
Adjacency Lag 6	0.00	0.02	0.00
Adjacency Lag 7	-0.05	0.00	-0.00
Adjacency Lag 8	0.00	0.00	0.00
Adjacency Lag 9	0.00	0.00	-0.00
Adjacency Lag 10	-0.08	0.00	-0.01

Table 7: SSTAR Bootstrap Values: Negeri Sembilan

6.7 Pahang

	2.5 Quantile	97.5 Quantile	Mean
Own Counts Lag 1	0.12	0.57	0.36
Own Counts Lag 2	0.00	0.54	0.23
Own Counts Lag 3	-0.07	0.20	0.02
Own Counts Lag 4	0.00	0.29	0.07
Own Counts Lag 5	-0.31	0.00	-0.06
Own Counts Lag 6	-0.26	0.11	-0.01
Own Counts Lag 7	-0.06	0.18	0.01
Own Counts Lag 8	-0.06	0.17	0.01
Own Counts Lag 9	-0.15	0.15	0.00
Own Counts Lag 10	-0.27	0.00	-0.05
Absolute Humidity Lag 1	0.00	18.52	3.42
Absolute Humidity Lag 2	0.00	23.94	3.86
Absolute Humidity Lag 3	-23.27	0.42	-2.74
Absolute Humidity Lag 4	0.00	14.67	1.77
Absolute Humidity Lag 5	-15.43	4.54	-0.93
Absolute Humidity Lag 6	0.00	13.43	1.37
Absolute Humidity Lag 7	-13.03	3.55	-0.86
Absolute Humidity Lag 8	0.00	17.76	3.04
Absolute Humidity Lag 9	0.00	10.65	1.37
Absolute Humidity Lag 10	-7.45	1.85	-0.38
Relative Humidity Lag 1	-12.80	0.00	-1.14
Relative Humidity Lag 2	0.00	15.12	2.07
Relative Humidity Lag 3	-13.83	0.00	-1.80
Relative Humidity Lag 4	-10.17	4.05	-0.51
Relative Humidity Lag 5	-12.98	2.75	-1.18
Relative Humidity Lag 6	-10.30	0.00	-0.93
Relative Humidity Lag 7	-3.40	9.98	0.60
Relative Humidity Lag 8	0.00	22.42	7.28
Relative Humidity Lag 9	-1.99	12.24	1.18
Relative Humidity Lag 10	-6.14	8.20	0.24
Total Precipitation Lag 1	-25.40	0.03	-4.44
Total Precipitation Lag 2	0.00	28.41	7.53
Total Precipitation Lag 3	-13.88	12.29	-1.01
Total Precipitation Lag 4	0.00	25.11	4.39
Total Precipitation Lag 5	-17.40	4.99	-1.80
Total Precipitation Lag 6	-7.16	10.00	0.35
Total Precipitation Lag 7	-17.22	2.35	-2.00
Total Precipitation Lag 8	-2.67	15.51	1.21
Total Precipitation Lag 9	0.00	34.04	9.10
Total Precipitation Lag 10	-2.14	21.84	3.13
Temperature Lag 1	0.00	18.33	3.53
Temperature Lag 2	-0.44	1.66	0.08
Temperature Lag 3	-1.56	2.75	0.13
Temperature Lag 4	0.00	13.12	1.30
Temperature Lag 5	-3.73	8.22	0.41
Temperature Lag 6	0.00	23.07	5.14
Temperature Lag 7	-13.05	5.50	-0.41
Temperature Lag 8	-5.46	0.00	-0.27
Temperature Lag 9	0.00	9.61	0.67
Temperature Lag 10	-6.72	4.11	-0.23
Connectivity Lag 1	0.00	0.17	0.02
Connectivity Lag 2	-0.18	0.00	-0.01
Connectivity Lag 3	0.00	0.13	0.02
Connectivity Lag 4	0.00	0.10	0.01
Connectivity Lag 5	0.00	0.09	0.01
Connectivity Lag 6	0.00	0.13	0.02
Connectivity Lag 7	0.00	0.23	0.04
Connectivity Lag 8	0.00	0.11	0.01
Connectivity Lag 9	0.00	0.10	0.01
Connectivity Lag 10	-0.10	0.02	-0.00
Adjacency Lag 1	-0.08	0.44	0.05
Adjacency Lag 2	-0.57	0.00	-0.08

Adjacency Lag 3	-0.31	0.15	-0.02
Adjacency Lag 4	-0.57	0.04	-0.06
Adjacency Lag 5	0.00	0.92	0.15
Adjacency Lag 6	0.00	0.85	0.36
Adjacency Lag 7	-0.13	0.38	0.03
Adjacency Lag 8	-0.24	0.08	-0.01
Adjacency Lag 9	-0.10	0.38	0.05
Adjacency Lag 10	-0.05	0.26	0.02

Table 8: SSTAR Bootstrap Values: Pahang

6.8 Perak

	2.5 Quantile	97.5 Quantile	Mean
Own Counts Lag 1	0.00	0.57	0.28
Own Counts Lag 2	0.00	0.32	0.11
Own Counts Lag 3	-0.03	0.26	0.04
Own Counts Lag 4	0.00	0.51	0.26
Own Counts Lag 5	-0.16	0.17	0.00
Own Counts Lag 6	-0.15	0.19	0.01
Own Counts Lag 7	-0.02	0.21	0.02
Own Counts Lag 8	-0.09	0.20	0.02
Own Counts Lag 9	-0.03	0.29	0.04
Own Counts Lag 10	-0.11	0.19	0.01
Absolute Humidity Lag 1	-22.23	15.68	-0.51
Absolute Humidity Lag 2	-23.14	11.05	-0.96
Absolute Humidity Lag 3	-21.95	14.94	-1.06
Absolute Humidity Lag 4	-2.50	24.28	1.82
Absolute Humidity Lag 5	-23.45	1.54	-1.97
Absolute Humidity Lag 6	0.00	36.69	5.63
Absolute Humidity Lag 7	-18.85	12.21	-0.54
Absolute Humidity Lag 8	-11.13	6.61	-0.26
Absolute Humidity Lag 9	0.00	22.15	2.17
Absolute Humidity Lag 10	-30.57	0.01	-2.56
Relative Humidity Lag 1	-25.03	5.16	-2.03
Relative Humidity Lag 2	-26.53	0.01	-1.66
Relative Humidity Lag 3	-20.00	2.89	-1.56
Relative Humidity Lag 4	-9.14	15.27	0.29
Relative Humidity Lag 5	-44.89	0.00	-9.78
Relative Humidity Lag 6	-1.94	9.53	0.37
Relative Humidity Lag 7	-16.74	0.90	-1.45
Relative Humidity Lag 8	-0.01	20.56	2.29
Relative Humidity Lag 9	-32.39	0.00	-1.99
Relative Humidity Lag 10	-17.92	3.58	-0.91
Total Precipitation Lag 1	-21.97	3.06	-3.58
Total Precipitation Lag 2	0.00	38.59	9.40
Total Precipitation Lag 3	-15.07	7.79	-0.77
Total Precipitation Lag 4	-3.24	25.05	4.42
Total Precipitation Lag 5	-10.89	24.36	1.78
Total Precipitation Lag 6	-14.88	13.80	-0.31
Total Precipitation Lag 7	-7.65	21.21	1.66
Total Precipitation Lag 8	-14.01	14.55	0.75
Total Precipitation Lag 9	-0.66	30.48	5.32
Total Precipitation Lag 10	0.00	45.19	10.68
Temperature Lag 1	0.00	12.49	0.67
Temperature Lag 2	-11.91	11.72	0.03
Temperature Lag 3	-3.80	14.49	0.58
Temperature Lag 4	-2.86	9.59	0.37
Temperature Lag 5	0.00	17.09	1.81
Temperature Lag 6	0.00	14.36	0.96
Temperature Lag 7	0.00	16.36	1.48
Temperature Lag 8	-25.85	0.67	-2.34
Temperature Lag 9	0.00	36.31	4.22
Temperature Lag 10	-12.01	0.00	-0.88
Connectivity Lag 1	0.00	0.30	0.06
Connectivity Lag 2	-0.39	0.00	-0.03
Connectivity Lag 3	0.00	0.31	0.03
Connectivity Lag 4	-0.34	0.00	-0.02
Connectivity Lag 5	0.00	0.13	0.01
Connectivity Lag 6	-0.11	0.03	-0.00
Connectivity Lag 7	-0.11	0.07	-0.01
Connectivity Lag 8	0.00	0.18	0.02
Connectivity Lag 9	0.00	0.41	0.06
Connectivity Lag 10	0.00	0.13	0.01
Adjacency Lag 1	-0.13	0.04	-0.01
Adjacency Lag 2	-0.24	0.00	-0.02

Adjacency Lag 3	-0.04	0.01	-0.00
Adjacency Lag 4	0.00	0.14	0.01
Adjacency Lag 5	-0.08	0.00	-0.00
Adjacency Lag 6	-0.21	0.00	-0.03
Adjacency Lag 7	-0.12	0.00	-0.01
Adjacency Lag 8	-0.13	0.02	-0.01
Adjacency Lag 9	-0.07	0.00	-0.00
Adjacency Lag 10	-0.15	0.00	-0.02

Table 9: SSTAR Bootstrap Values: Perak

6.9 Perlis

	2.5 Quantile	97.5 Quantile	Mean
Own Counts Lag 1	-0.12	0.19	0.02
Own Counts Lag 2	-0.25	0.13	-0.03
Own Counts Lag 3	-0.23	0.10	-0.03
Own Counts Lag 4	-0.11	0.24	0.04
Own Counts Lag 5	0.00	0.40	0.13
Own Counts Lag 6	-0.11	0.26	0.02
Own Counts Lag 7	-0.26	0.08	-0.04
Own Counts Lag 8	-0.08	0.29	0.05
Own Counts Lag 9	-0.17	0.09	-0.02
Own Counts Lag 10	-0.27	0.04	-0.07
Absolute Humidity Lag 1	-1.83	2.10	0.02
Absolute Humidity Lag 2	-2.92	3.83	0.11
Absolute Humidity Lag 3	-1.84	1.38	0.01
Absolute Humidity Lag 4	-1.75	3.07	0.14
Absolute Humidity Lag 5	-1.42	5.06	0.45
Absolute Humidity Lag 6	-7.00	0.00	-0.67
Absolute Humidity Lag 7	-2.22	1.50	-0.00
Absolute Humidity Lag 8	0.00	6.32	1.04
Absolute Humidity Lag 9	0.00	3.67	0.56
Absolute Humidity Lag 10	0.00	4.70	0.72
Relative Humidity Lag 1	0.00	4.31	0.81
Relative Humidity Lag 2	-3.69	0.33	-0.23
Relative Humidity Lag 3	-2.96	0.00	-0.24
Relative Humidity Lag 4	-1.00	0.66	-0.01
Relative Humidity Lag 5	-0.65	0.85	0.01
Relative Humidity Lag 6	-3.39	0.00	-0.24
Relative Humidity Lag 7	0.00	2.44	0.19
Relative Humidity Lag 8	0.00	1.27	0.07
Relative Humidity Lag 9	-0.87	0.00	-0.05
Relative Humidity Lag 10	0.00	0.15	-0.00
Total Precipitation Lag 1	-2.48	1.76	-0.08
Total Precipitation Lag 2	-1.22	2.65	0.22
Total Precipitation Lag 3	-3.82	0.00	-0.86
Total Precipitation Lag 4	-2.49	1.29	-0.20
Total Precipitation Lag 5	-3.58	0.22	-0.61
Total Precipitation Lag 6	-2.97	2.27	-0.13
Total Precipitation Lag 7	-2.85	3.77	0.56
Total Precipitation Lag 8	-2.18	2.54	0.21
Total Precipitation Lag 9	0.00	5.35	1.26
Total Precipitation Lag 10	-0.21	4.48	1.06
Temperature Lag 1	-7.33	0.00	-1.72
Temperature Lag 2	-1.13	1.25	-0.01
Temperature Lag 3	0.00	2.85	0.22
Temperature Lag 4	-2.30	0.92	-0.08
Temperature Lag 5	-0.00	3.06	0.30
Temperature Lag 6	-0.47	2.26	0.13
Temperature Lag 7	-2.99	0.00	-0.31
Temperature Lag 8	0.00	4.30	0.49
Temperature Lag 9	0.00	5.10	0.87
Temperature Lag 10	0.00	5.67	0.72
Connectivity Lag 1	0.00	0.12	0.03
Connectivity Lag 2	-0.01	0.03	0.00
Connectivity Lag 3	-0.06	0.00	-0.00
Connectivity Lag 4	0.00	0.13	0.03
Connectivity Lag 5	-0.05	0.02	-0.00
Connectivity Lag 6	-0.13	0.00	-0.01
Connectivity Lag 7	0.00	0.14	0.02
Connectivity Lag 8	0.00	0.17	0.05
Connectivity Lag 9	0.00	0.03	0.00
Connectivity Lag 10	-0.06	0.00	-0.00
Adjacency Lag 1	-0.12	0.07	-0.00
Adjacency Lag 2	-0.06	0.08	0.00

Adjacency Lag 3	-0.18	0.00	-0.03
Adjacency Lag 4	-0.01	0.24	0.04
Adjacency Lag 5	-0.07	0.10	0.01
Adjacency Lag 6	-0.15	0.03	-0.02
Adjacency Lag 7	-0.13	0.08	-0.01
Adjacency Lag 8	-0.04	0.12	0.02
Adjacency Lag 9	-0.02	0.11	0.01
Adjacency Lag 10	-0.14	0.01	-0.02

Table 10: SSTAR Bootstrap Values: Perlis

6.10 Pulau Pinang

	2.5 Quantile	97.5 Quantile	Mean
Own Counts Lag 1	0.09	0.65	0.35
Own Counts Lag 2	0.00	0.37	0.11
Own Counts Lag 3	-0.13	0.24	0.01
Own Counts Lag 4	0.00	0.36	0.12
Own Counts Lag 5	-0.03	0.24	0.05
Own Counts Lag 6	0.00	0.39	0.13
Own Counts Lag 7	-0.33	0.02	-0.03
Own Counts Lag 8	-0.01	0.27	0.03
Own Counts Lag 9	-0.08	0.31	0.03
Own Counts Lag 10	-0.35	0.02	-0.05
Absolute Humidity Lag 1	0.00	36.23	7.88
Absolute Humidity Lag 2	-8.03	21.80	1.64
Absolute Humidity Lag 3	-19.70	7.36	-1.82
Absolute Humidity Lag 4	-32.05	2.27	-3.75
Absolute Humidity Lag 5	-24.55	13.56	-1.25
Absolute Humidity Lag 6	-21.39	16.72	-0.45
Absolute Humidity Lag 7	-10.13	22.45	1.43
Absolute Humidity Lag 8	-7.79	19.66	1.66
Absolute Humidity Lag 9	0.00	39.92	6.76
Absolute Humidity Lag 10	-21.61	4.91	-1.49
Relative Humidity Lag 1	-3.82	1.31	-0.17
Relative Humidity Lag 2	-24.51	1.52	-2.49
Relative Humidity Lag 3	-13.23	18.49	-0.17
Relative Humidity Lag 4	-24.68	0.00	-5.29
Relative Humidity Lag 5	-33.18	0.00	-7.56
Relative Humidity Lag 6	-1.28	18.66	1.79
Relative Humidity Lag 7	-17.42	4.47	-1.38
Relative Humidity Lag 8	0.00	25.26	4.16
Relative Humidity Lag 9	0.00	28.50	2.78
Relative Humidity Lag 10	0.00	26.47	4.29
Total Precipitation Lag 1	-6.46	12.23	0.75
Total Precipitation Lag 2	0.00	21.08	5.08
Total Precipitation Lag 3	-8.98	11.00	0.39
Total Precipitation Lag 4	-5.92	10.66	0.53
Total Precipitation Lag 5	0.00	27.12	5.37
Total Precipitation Lag 6	-10.23	8.53	0.14
Total Precipitation Lag 7	-5.52	18.85	1.88
Total Precipitation Lag 8	-12.69	7.09	-0.89
Total Precipitation Lag 9	-14.49	2.73	-3.07
Total Precipitation Lag 10	-15.10	6.98	-0.91
Temperature Lag 1	0.00	22.10	3.92
Temperature Lag 2	0.00	23.87	2.79
Temperature Lag 3	-17.74	5.95	-0.90
Temperature Lag 4	0.00	19.73	1.81
Temperature Lag 5	0.00	33.11	4.75
Temperature Lag 6	-26.02	0.00	-2.44
Temperature Lag 7	0.00	15.79	1.85
Temperature Lag 8	-19.61	0.00	-1.79
Temperature Lag 9	-0.22	9.54	0.77
Temperature Lag 10	-24.01	0.00	-3.27
Connectivity Lag 1	-0.13	0.11	0.00
Connectivity Lag 2	0.00	0.30	0.03
Connectivity Lag 3	-0.21	0.00	-0.01
Connectivity Lag 4	-0.32	0.04	-0.02
Connectivity Lag 5	-0.31	0.00	-0.02
Connectivity Lag 6	-0.04	0.24	0.01
Connectivity Lag 7	0.00	0.44	0.04
Connectivity Lag 8	-0.04	0.19	0.01
Connectivity Lag 9	-0.11	0.18	0.00
Connectivity Lag 10	-0.51	0.00	-0.05
Adjacency Lag 1	0.00	0.21	0.04
Adjacency Lag 2	0.00	0.18	0.04

Adjacency Lag 3	-0.10	0.08	-0.00
Adjacency Lag 4	0.00	0.20	0.02
Adjacency Lag 5	-0.09	0.07	-0.00
Adjacency Lag 6	0.00	0.25	0.04
Adjacency Lag 7	-0.16	0.03	-0.01
Adjacency Lag 8	-0.11	0.07	-0.00
Adjacency Lag 9	-0.13	0.06	-0.01
Adjacency Lag 10	0.00	0.21	0.02

Table 11: SSTAR Bootstrap Values: Pulau Pinang

6.11 Sabah

	2.5 Quantile	97.5 Quantile	Mean
Own Counts Lag 1	0.12	0.67	0.48
Own Counts Lag 2	-0.11	0.31	0.08
Own Counts Lag 3	-0.05	0.38	0.09
Own Counts Lag 4	-0.20	0.20	0.01
Own Counts Lag 5	-0.03	0.35	0.06
Own Counts Lag 6	-0.36	0.07	-0.04
Own Counts Lag 7	-0.35	0.09	-0.03
Own Counts Lag 8	-0.67	0.00	-0.25
Own Counts Lag 9	-0.43	0.00	-0.06
Own Counts Lag 10	-0.61	0.00	-0.11
Absolute Humidity Lag 1	-12.28	10.28	-0.01
Absolute Humidity Lag 2	-27.44	17.74	-0.88
Absolute Humidity Lag 3	-28.07	13.73	-1.93
Absolute Humidity Lag 4	-11.27	16.15	0.64
Absolute Humidity Lag 5	-22.01	7.37	-1.72
Absolute Humidity Lag 6	-17.12	9.02	-0.91
Absolute Humidity Lag 7	-6.29	33.65	5.13
Absolute Humidity Lag 8	-2.96	24.54	2.80
Absolute Humidity Lag 9	-26.20	2.13	-2.01
Absolute Humidity Lag 10	-16.02	15.54	-0.06
Relative Humidity Lag 1	-46.31	0.00	-7.00
Relative Humidity Lag 2	-12.85	14.06	-0.19
Relative Humidity Lag 3	-8.09	14.93	1.21
Relative Humidity Lag 4	-10.93	19.54	1.05
Relative Humidity Lag 5	-20.66	4.00	-2.82
Relative Humidity Lag 6	-18.94	0.00	-2.77
Relative Humidity Lag 7	-8.41	17.31	1.54
Relative Humidity Lag 8	-3.89	20.82	1.97
Relative Humidity Lag 9	-2.28	17.70	2.47
Relative Humidity Lag 10	0.00	19.55	2.83
Total Precipitation Lag 1	-14.38	31.22	1.01
Total Precipitation Lag 2	-21.90	12.08	-2.88
Total Precipitation Lag 3	-22.46	6.47	-2.04
Total Precipitation Lag 4	-34.11	4.49	-4.88
Total Precipitation Lag 5	-2.86	25.67	3.72
Total Precipitation Lag 6	-2.33	38.21	5.13
Total Precipitation Lag 7	0.00	29.77	5.63
Total Precipitation Lag 8	-21.10	9.24	-2.02
Total Precipitation Lag 9	-27.82	0.00	-5.14
Total Precipitation Lag 10	-27.53	3.87	-4.01
Temperature Lag 1	0.00	28.16	5.20
Temperature Lag 2	-11.02	15.87	0.39
Temperature Lag 3	-17.00	0.00	-1.40
Temperature Lag 4	-14.25	4.24	-0.92
Temperature Lag 5	-3.86	10.88	0.67
Temperature Lag 6	0.00	26.31	2.27
Temperature Lag 7	0.00	17.62	0.97
Temperature Lag 8	-10.39	8.67	0.04
Temperature Lag 9	-28.85	0.00	-3.49
Temperature Lag 10	-23.95	0.00	-2.32
Connectivity Lag 1	0.00	1.07	0.16
Connectivity Lag 2	-0.85	0.00	-0.07
Connectivity Lag 3	0.00	0.63	0.06
Connectivity Lag 4	0.00	0.54	0.03
Connectivity Lag 5	-0.00	0.57	0.04
Connectivity Lag 6	-0.54	0.20	-0.03
Connectivity Lag 7	-0.55	0.22	-0.03
Connectivity Lag 8	-0.34	0.48	0.01
Connectivity Lag 9	-0.06	0.66	0.04
Connectivity Lag 10	-0.22	0.27	0.00
Adjacency Lag 1	0.00	0.59	0.14
Adjacency Lag 2	0.00	0.78	0.11

Adjacency Lag 3	0.00	0.79	0.12
Adjacency Lag 4	-0.16	0.34	0.01
Adjacency Lag 5	0.00	0.86	0.11
Adjacency Lag 6	0.00	0.97	0.13
Adjacency Lag 7	-0.80	0.17	-0.05
Adjacency Lag 8	0.00	1.30	0.20
Adjacency Lag 9	-0.88	0.00	-0.13
Adjacency Lag 10	-0.18	0.29	0.01

Table 12: SSTAR Bootstrap Values: Sabah

6.12 Sarawak

	2.5 Quantile	97.5 Quantile	Mean
Own Counts Lag 1	0.19	0.80	0.54
Own Counts Lag 2	0.00	0.53	0.15
Own Counts Lag 3	-0.09	0.09	0.00
Own Counts Lag 4	-0.10	0.21	0.01
Own Counts Lag 5	-0.19	0.10	-0.01
Own Counts Lag 6	-0.13	0.05	-0.01
Own Counts Lag 7	-0.20	0.00	-0.02
Own Counts Lag 8	-0.29	0.00	-0.05
Own Counts Lag 9	-0.21	0.03	-0.01
Own Counts Lag 10	-0.05	0.11	0.00
Absolute Humidity Lag 1	-23.37	0.00	-3.14
Absolute Humidity Lag 2	-8.06	22.28	1.71
Absolute Humidity Lag 3	-26.14	0.00	-2.93
Absolute Humidity Lag 4	0.00	34.85	6.32
Absolute Humidity Lag 5	-10.53	12.79	0.37
Absolute Humidity Lag 6	-43.67	0.00	-5.73
Absolute Humidity Lag 7	-25.22	1.17	-2.53
Absolute Humidity Lag 8	-16.67	11.01	-0.53
Absolute Humidity Lag 9	-10.73	15.40	0.36
Absolute Humidity Lag 10	-14.08	9.24	-0.71
Relative Humidity Lag 1	-10.48	21.85	1.13
Relative Humidity Lag 2	-6.25	23.74	1.66
Relative Humidity Lag 3	-53.33	0.00	-11.60
Relative Humidity Lag 4	-5.60	20.95	1.79
Relative Humidity Lag 5	-12.78	11.10	-0.31
Relative Humidity Lag 6	-6.23	19.81	1.35
Relative Humidity Lag 7	-25.95	1.52	-2.99
Relative Humidity Lag 8	-14.36	16.94	-0.05
Relative Humidity Lag 9	-16.32	4.30	-1.39
Relative Humidity Lag 10	-1.12	38.04	4.46
Total Precipitation Lag 1	-26.18	3.39	-3.31
Total Precipitation Lag 2	-20.33	4.73	-2.25
Total Precipitation Lag 3	-14.16	15.45	-0.88
Total Precipitation Lag 4	-17.65	6.23	-1.81
Total Precipitation Lag 5	-5.96	16.35	1.37
Total Precipitation Lag 6	-23.76	7.14	-2.23
Total Precipitation Lag 7	-15.07	5.65	-1.82
Total Precipitation Lag 8	-25.47	0.00	-5.31
Total Precipitation Lag 9	-17.22	8.23	-1.41
Total Precipitation Lag 10	-40.42	0.00	-7.39
Temperature Lag 1	-38.20	0.00	-5.88
Temperature Lag 2	-16.07	9.54	-0.53
Temperature Lag 3	-2.66	22.58	1.74
Temperature Lag 4	-2.00	14.42	0.86
Temperature Lag 5	-6.78	11.24	0.21
Temperature Lag 6	-53.96	0.00	-11.07
Temperature Lag 7	-6.74	7.47	0.23
Temperature Lag 8	-10.67	9.91	-0.28
Temperature Lag 9	-2.72	14.60	0.89
Temperature Lag 10	-24.69	0.00	-3.10
Connectivity Lag 1	0.00	1.30	0.48
Connectivity Lag 2	-0.12	0.23	0.00
Connectivity Lag 3	0.00	0.48	0.05
Connectivity Lag 4	0.00	0.54	0.07
Connectivity Lag 5	-0.82	0.00	-0.07
Connectivity Lag 6	0.00	0.64	0.06
Connectivity Lag 7	0.00	0.35	0.03
Connectivity Lag 8	-0.13	0.10	-0.01
Connectivity Lag 9	0.00	0.36	0.03
Connectivity Lag 10	-0.10	0.40	0.04
Adjacency Lag 1	-0.91	0.15	-0.09
Adjacency Lag 2	-0.10	0.65	0.11

Adjacency Lag 3	-0.40	0.58	0.03
Adjacency Lag 4	0.00	2.64	1.07
Adjacency Lag 5	-1.26	0.36	-0.19
Adjacency Lag 6	-1.11	0.00	-0.19
Adjacency Lag 7	-0.33	0.70	0.04
Adjacency Lag 8	-0.83	0.30	-0.09
Adjacency Lag 9	-0.88	0.09	-0.10
Adjacency Lag 10	-0.87	0.42	-0.10

Table 13: SSTAR Bootstrap Values: Sarawak

6.13 Selangor

	2.5 Quantile	97.5 Quantile	Mean
Own Counts Lag 1	0.30	0.70	0.52
Own Counts Lag 2	-0.03	0.14	0.01
Own Counts Lag 3	0.00	0.24	0.05
Own Counts Lag 4	-0.08	0.19	0.02
Own Counts Lag 5	0.00	0.30	0.10
Own Counts Lag 6	0.00	0.25	0.06
Own Counts Lag 7	-0.23	0.03	-0.01
Own Counts Lag 8	-0.10	0.08	0.00
Own Counts Lag 9	-0.02	0.17	0.01
Own Counts Lag 10	0.00	0.17	0.02
Absolute Humidity Lag 1	-89.09	9.50	-16.28
Absolute Humidity Lag 2	-114.49	0.00	-25.76
Absolute Humidity Lag 3	-46.42	71.11	2.57
Absolute Humidity Lag 4	-45.96	74.53	5.86
Absolute Humidity Lag 5	0.00	154.00	57.13
Absolute Humidity Lag 6	-46.87	65.36	3.23
Absolute Humidity Lag 7	-56.28	29.17	-3.16
Absolute Humidity Lag 8	-44.64	58.71	2.75
Absolute Humidity Lag 9	0.00	61.42	5.46
Absolute Humidity Lag 10	-139.10	0.00	-56.64
Relative Humidity Lag 1	-16.52	60.44	3.54
Relative Humidity Lag 2	-50.01	5.65	-3.81
Relative Humidity Lag 3	0.00	85.16	16.35
Relative Humidity Lag 4	-10.46	40.48	2.58
Relative Humidity Lag 5	0.00	63.49	7.54
Relative Humidity Lag 6	-38.55	47.19	0.48
Relative Humidity Lag 7	-70.08	47.32	-1.42
Relative Humidity Lag 8	-106.13	4.55	-12.97
Relative Humidity Lag 9	0.00	139.87	38.12
Relative Humidity Lag 10	-59.23	0.00	-6.05
Total Precipitation Lag 1	-106.76	0.00	-27.84
Total Precipitation Lag 2	-53.37	27.45	-7.87
Total Precipitation Lag 3	-22.34	58.26	8.75
Total Precipitation Lag 4	-4.90	65.78	14.84
Total Precipitation Lag 5	-55.75	20.45	-6.99
Total Precipitation Lag 6	-36.85	38.21	0.56
Total Precipitation Lag 7	-12.77	73.68	10.45
Total Precipitation Lag 8	-26.97	79.91	7.58
Total Precipitation Lag 9	-109.05	5.80	-21.24
Total Precipitation Lag 10	-80.16	5.84	-14.47
Temperature Lag 1	-81.37	0.00	-12.10
Temperature Lag 2	-36.90	9.32	-2.14
Temperature Lag 3	-73.36	0.00	-10.80
Temperature Lag 4	-24.69	37.75	1.15
Temperature Lag 5	0.00	53.34	4.59
Temperature Lag 6	-32.71	51.08	1.94
Temperature Lag 7	-31.58	48.89	1.74
Temperature Lag 8	0.00	88.50	14.60
Temperature Lag 9	-84.09	0.00	-10.69
Temperature Lag 10	-80.48	0.00	-12.68
Connectivity Lag 1	-0.88	0.85	-0.00
Connectivity Lag 2	-0.93	0.00	-0.07
Connectivity Lag 3	-0.22	0.56	0.02
Connectivity Lag 4	0.00	1.62	0.39
Connectivity Lag 5	-0.50	0.20	-0.02
Connectivity Lag 6	-0.56	0.52	-0.00
Connectivity Lag 7	-1.16	0.00	-0.18
Connectivity Lag 8	-1.16	0.00	-0.13
Connectivity Lag 9	-0.48	0.50	0.00
Connectivity Lag 10	-0.42	0.54	0.02
Adjacency Lag 1	0.00	1.76	0.55
Adjacency Lag 2	-0.37	0.57	0.01

Adjacency Lag 3	-0.23	0.71	0.06
Adjacency Lag 4	0.00	1.20	0.22
Adjacency Lag 5	-1.10	0.00	-0.13
Adjacency Lag 6	-0.32	0.55	0.01
Adjacency Lag 7	-0.81	0.12	-0.06
Adjacency Lag 8	-1.36	0.00	-0.40
Adjacency Lag 9	-0.32	0.46	0.02
Adjacency Lag 10	-0.25	0.51	0.02

Table 14: SSTAR Bootstrap Values: Selangor

6.14 Singapore

	2.5 Quantile	97.5 Quantile	Mean
Own Counts Lag 1	0.33	0.82	0.61
Own Counts Lag 2	-0.19	0.26	0.02
Own Counts Lag 3	0.00	0.37	0.09
Own Counts Lag 4	-0.27	0.00	-0.03
Own Counts Lag 5	-0.18	0.17	-0.00
Own Counts Lag 6	-0.20	0.11	-0.01
Own Counts Lag 7	-0.14	0.23	0.01
Own Counts Lag 8	-0.17	0.08	-0.01
Own Counts Lag 9	-0.09	0.11	-0.00
Own Counts Lag 10	-0.10	0.10	-0.00
Absolute Humidity Lag 1	-54.56	6.51	-9.03
Absolute Humidity Lag 2	-54.56	6.35	-6.38
Absolute Humidity Lag 3	-29.02	24.47	-0.84
Absolute Humidity Lag 4	0.00	79.06	16.73
Absolute Humidity Lag 5	0.00	87.86	28.22
Absolute Humidity Lag 6	-31.25	31.59	0.53
Absolute Humidity Lag 7	0.00	72.35	17.79
Absolute Humidity Lag 8	0.00	98.13	32.10
Absolute Humidity Lag 9	0.00	66.71	14.11
Absolute Humidity Lag 10	-9.19	22.63	1.23
Relative Humidity Lag 1	-31.88	4.90	-5.27
Relative Humidity Lag 2	-6.22	38.38	4.64
Relative Humidity Lag 3	-35.95	13.00	-4.10
Relative Humidity Lag 4	-32.13	36.27	-0.21
Relative Humidity Lag 5	0.00	44.75	5.31
Relative Humidity Lag 6	-46.39	8.51	-9.72
Relative Humidity Lag 7	-5.54	22.45	1.81
Relative Humidity Lag 8	-6.94	22.12	1.31
Relative Humidity Lag 9	-1.89	44.00	7.46
Relative Humidity Lag 10	-29.55	6.25	-4.42
Total Precipitation Lag 1	-20.49	26.64	1.23
Total Precipitation Lag 2	-84.31	0.00	-25.14
Total Precipitation Lag 3	-11.06	45.80	7.70
Total Precipitation Lag 4	-60.21	1.69	-15.92
Total Precipitation Lag 5	-57.64	0.00	-12.97
Total Precipitation Lag 6	-30.57	33.12	0.76
Total Precipitation Lag 7	-37.97	23.95	-2.13
Total Precipitation Lag 8	-19.22	34.50	1.67
Total Precipitation Lag 9	-33.52	33.99	0.40
Total Precipitation Lag 10	-51.38	4.84	-11.97
Temperature Lag 1	-22.69	17.96	-0.64
Temperature Lag 2	-84.67	0.00	-11.78
Temperature Lag 3	-3.14	35.58	2.46
Temperature Lag 4	0.00	50.90	7.29
Temperature Lag 5	0.00	38.84	4.04
Temperature Lag 6	0.00	40.97	5.38
Temperature Lag 7	0.00	38.36	4.43
Temperature Lag 8	0.00	48.41	7.21
Temperature Lag 9	-3.89	33.45	1.91
Temperature Lag 10	0.00	55.22	9.45
Connectivity Lag 1	-0.11	0.23	0.01
Connectivity Lag 2	-0.09	0.12	0.00
Connectivity Lag 3	-0.27	0.18	-0.01
Connectivity Lag 4	-0.08	0.29	0.02
Connectivity Lag 5	-0.40	0.00	-0.05
Connectivity Lag 6	-0.28	0.03	-0.02
Connectivity Lag 7	-0.56	0.00	-0.07
Connectivity Lag 8	-0.57	0.00	-0.07
Connectivity Lag 9	-0.43	0.00	-0.05
Connectivity Lag 10	-0.31	0.05	-0.02
Adjacency Lag 1	-0.12	0.09	-0.00
Adjacency Lag 2	-0.26	0.00	-0.02

Adjacency Lag 3	-0.26	0.00	-0.02
Adjacency Lag 4	-0.13	0.09	-0.00
Adjacency Lag 5	-0.39	0.03	-0.02
Adjacency Lag 6	0.00	0.37	0.03
Adjacency Lag 7	-0.16	0.00	-0.01
Adjacency Lag 8	0.00	0.74	0.07
Adjacency Lag 9	0.00	0.12	0.00
Adjacency Lag 10	0.00	0.95	0.23

Table 15: SSTAR Bootstrap Values: Singapore

6.15 Terengganu

	2.5 Quantile	97.5 Quantile	Mean
Own Counts Lag 1	0.00	0.41	0.15
Own Counts Lag 2	-0.35	0.00	-0.06
Own Counts Lag 3	-0.27	0.09	-0.05
Own Counts Lag 4	-0.26	0.04	-0.03
Own Counts Lag 5	-0.14	0.21	-0.00
Own Counts Lag 6	-0.25	0.04	-0.03
Own Counts Lag 7	-0.16	0.08	-0.01
Own Counts Lag 8	-0.16	0.18	0.00
Own Counts Lag 9	-0.29	0.02	-0.06
Own Counts Lag 10	-0.21	0.06	-0.02
Absolute Humidity Lag 1	-25.38	0.00	-4.39
Absolute Humidity Lag 2	-33.56	3.31	-6.46
Absolute Humidity Lag 3	-5.42	21.00	1.62
Absolute Humidity Lag 4	-15.06	19.95	0.70
Absolute Humidity Lag 5	-32.74	0.00	-4.94
Absolute Humidity Lag 6	-25.89	1.92	-3.64
Absolute Humidity Lag 7	-2.27	21.99	2.06
Absolute Humidity Lag 8	-13.14	10.74	0.02
Absolute Humidity Lag 9	-14.07	4.17	-0.93
Absolute Humidity Lag 10	-1.52	16.08	1.49
Relative Humidity Lag 1	-16.42	4.08	-1.08
Relative Humidity Lag 2	-27.95	0.55	-5.51
Relative Humidity Lag 3	-30.43	2.94	-3.70
Relative Humidity Lag 4	0.00	23.12	3.98
Relative Humidity Lag 5	-25.81	0.30	-3.39
Relative Humidity Lag 6	-14.47	7.20	-0.79
Relative Humidity Lag 7	-17.04	2.42	-1.23
Relative Humidity Lag 8	-3.06	12.40	1.15
Relative Humidity Lag 9	-14.77	2.48	-2.09
Relative Humidity Lag 10	-7.57	9.21	0.19
Total Precipitation Lag 1	-10.77	15.39	1.42
Total Precipitation Lag 2	-6.69	17.63	1.25
Total Precipitation Lag 3	-0.08	31.94	5.12
Total Precipitation Lag 4	-14.28	7.46	-1.06
Total Precipitation Lag 5	-8.60	19.47	1.23
Total Precipitation Lag 6	0.61	37.00	16.51
Total Precipitation Lag 7	-5.64	18.14	2.73
Total Precipitation Lag 8	-6.50	18.96	2.45
Total Precipitation Lag 9	-19.23	5.23	-2.86
Total Precipitation Lag 10	-13.84	11.14	-0.41
Temperature Lag 1	-14.75	0.00	-1.27
Temperature Lag 2	-8.07	1.38	-0.27
Temperature Lag 3	0.00	21.98	2.06
Temperature Lag 4	-15.58	0.00	-1.20
Temperature Lag 5	-4.02	4.66	0.12
Temperature Lag 6	-10.54	1.53	-0.68
Temperature Lag 7	0.00	25.09	2.60
Temperature Lag 8	-11.16	2.50	-0.76
Temperature Lag 9	-0.58	11.21	0.76
Temperature Lag 10	-2.62	8.40	0.54
Connectivity Lag 1	-0.06	0.20	0.01
Connectivity Lag 2	-0.49	0.00	-0.05
Connectivity Lag 3	-0.01	0.29	0.02
Connectivity Lag 4	-0.07	0.18	0.00
Connectivity Lag 5	0.00	0.27	0.02
Connectivity Lag 6	-0.01	0.21	0.01
Connectivity Lag 7	-0.06	0.08	0.00
Connectivity Lag 8	-0.22	0.07	-0.02
Connectivity Lag 9	-0.07	0.20	0.01
Connectivity Lag 10	-0.29	0.03	-0.03
Adjacency Lag 1	-0.12	0.27	0.02
Adjacency Lag 2	-0.39	0.00	-0.08

Adjacency Lag 3	-0.01	0.28	0.03
Adjacency Lag 4	-0.06	0.29	0.03
Adjacency Lag 5	0.00	0.58	0.19
Adjacency Lag 6	0.00	0.90	0.48
Adjacency Lag 7	0.00	0.59	0.08
Adjacency Lag 8	-0.09	0.14	0.00
Adjacency Lag 9	-0.19	0.18	-0.00
Adjacency Lag 10	-0.02	0.31	0.02

Table 16: SSTAR Bootstrap Values: Terengganu

7 Climate Sensitivity Analysis

	Johor Mean	Johor Max	Johor Min
Own Counts Lag 1	0.90	0.90	0.92
Own Counts Lag 2	0.04	0.04	0.02
Own Counts Lag 3	0.00	0.00	0.00
Own Counts Lag 4	0.00	0.00	0.00
Own Counts Lag 5	0.00	0.00	0.00
Own Counts Lag 6	0.00	0.00	0.00
Own Counts Lag 7	0.00	0.00	0.00
Own Counts Lag 8	0.00	0.00	0.00
Own Counts Lag 9	0.00	0.00	0.00
Own Counts Lag 10	0.00	0.00	0.00
Absolute Humidity Lag 1	0.00	0.00	0.00
Absolute Humidity Lag 2	0.02	0.01	0.00
Absolute Humidity Lag 3	0.00	0.01	0.01
Absolute Humidity Lag 4	0.00	0.00	0.00
Absolute Humidity Lag 5	0.00	0.00	0.00
Absolute Humidity Lag 6	0.00	0.00	0.00
Absolute Humidity Lag 7	0.00	0.00	0.00
Absolute Humidity Lag 8	0.00	0.00	0.00
Absolute Humidity Lag 9	0.00	0.00	0.00
Absolute Humidity Lag 10	0.00	0.00	0.00
Relative Humidity Lag 1	0.00	0.00	0.00
Relative Humidity Lag 2	0.00	0.00	0.00
Relative Humidity Lag 3	0.00	0.00	0.00
Relative Humidity Lag 4	0.00	0.00	0.00
Relative Humidity Lag 5	0.00	0.00	0.00
Relative Humidity Lag 6	0.00	0.00	0.00
Relative Humidity Lag 7	0.00	0.00	0.00
Relative Humidity Lag 8	0.00	0.00	0.00
Relative Humidity Lag 9	0.00	0.00	0.00
Relative Humidity Lag 10	-0.00	0.00	0.00
Total Precipitation Lag 1	0.03	0.02	0.00
Total Precipitation Lag 2	0.02	0.02	0.00
Total Precipitation Lag 3	0.00	0.00	0.00
Total Precipitation Lag 4	0.00	0.00	0.00
Total Precipitation Lag 5	0.00	0.00	0.01
Total Precipitation Lag 6	0.00	0.00	0.00
Total Precipitation Lag 7	0.00	0.00	0.00
Total Precipitation Lag 8	0.00	0.00	0.00
Total Precipitation Lag 9	0.00	0.00	0.00
Total Precipitation Lag 10	-0.00	0.00	0.00
Temperature Lag 1	0.00	0.00	0.00
Temperature Lag 2	0.00	0.00	0.00
Temperature Lag 3	0.00	0.00	0.00
Temperature Lag 4	0.00	0.00	0.00
Temperature Lag 5	0.00	0.00	0.00
Temperature Lag 6	0.00	0.00	0.00
Temperature Lag 7	0.00	0.00	0.00
Temperature Lag 8	0.00	0.00	0.00
Temperature Lag 9	0.00	0.00	0.00
Temperature Lag 10	0.00	0.00	0.00

Connectivity Lag 1	0.01	0.00	0.00
Connectivity Lag 2	0.00	0.00	0.00
Connectivity Lag 3	0.00	0.00	0.00
Connectivity Lag 4	0.00	0.00	0.00
Connectivity Lag 5	0.00	0.00	0.00
Connectivity Lag 6	0.00	0.00	0.00
Connectivity Lag 7	0.00	0.00	0.00
Connectivity Lag 8	0.00	0.00	0.00
Connectivity Lag 9	0.00	0.00	0.00
Connectivity Lag 10	0.00	0.00	0.00
Adjacency Lag 1	0.00	0.00	0.00
Adjacency Lag 2	0.00	0.00	0.00
Adjacency Lag 3	0.00	0.00	0.00
Adjacency Lag 4	0.00	0.00	0.00
Adjacency Lag 5	0.00	0.00	0.00
Adjacency Lag 6	0.00	0.00	0.00
Adjacency Lag 7	0.00	0.00	0.00
Adjacency Lag 8	0.00	0.00	0.00
Adjacency Lag 9	0.00	0.00	0.00
Adjacency Lag 10	0.00	0.00	0.00

	Kedah Mean	Kedah Max	Kedah Min
Own Counts Lag 1	0.43	0.43	0.43
Own Counts Lag 2	0.01	0.00	0.00
Own Counts Lag 3	0.10	0.09	0.09
Own Counts Lag 4	0.12	0.12	0.13
Own Counts Lag 5	0.04	0.03	0.04
Own Counts Lag 6	0.00	0.00	0.00
Own Counts Lag 7	0.00	0.00	0.00
Own Counts Lag 8	0.00	0.00	0.00
Own Counts Lag 9	0.00	0.00	0.00
Own Counts Lag 10	0.00	0.00	0.00
Absolute Humidity Lag 1	0.00	0.00	0.00
Absolute Humidity Lag 2	0.00	0.00	0.00
Absolute Humidity Lag 3	0.03	0.00	0.00
Absolute Humidity Lag 4	0.01	0.00	0.00
Absolute Humidity Lag 5	0.00	0.00	0.00
Absolute Humidity Lag 6	0.00	0.00	0.00
Absolute Humidity Lag 7	0.00	0.00	0.00
Absolute Humidity Lag 8	0.00	0.00	0.00
Absolute Humidity Lag 9	0.00	0.00	-0.00
Absolute Humidity Lag 10	-0.04	0.00	0.00
Relative Humidity Lag 1	0.00	0.00	0.00
Relative Humidity Lag 2	0.00	0.01	0.02
Relative Humidity Lag 3	0.00	0.00	0.00
Relative Humidity Lag 4	0.00	0.00	0.00
Relative Humidity Lag 5	0.00	0.00	0.00
Relative Humidity Lag 6	0.00	0.00	0.00
Relative Humidity Lag 7	0.00	0.00	-0.01
Relative Humidity Lag 8	0.00	0.00	0.00
Relative Humidity Lag 9	0.00	0.00	0.00
Relative Humidity Lag 10	0.00	0.00	-0.07
Total Precipitation Lag 1	-0.02	0.00	0.00
Total Precipitation Lag 2	0.03	0.00	0.02
Total Precipitation Lag 3	0.00	0.00	0.00
Total Precipitation Lag 4	0.00	0.00	0.00
Total Precipitation Lag 5	0.00	0.00	0.00
Total Precipitation Lag 6	0.03	0.00	0.03
Total Precipitation Lag 7	0.00	0.00	0.00
Total Precipitation Lag 8	0.00	0.00	0.00
Total Precipitation Lag 9	0.00	0.00	0.00
Total Precipitation Lag 10	0.00	0.00	0.08
Temperature Lag 1	0.00	0.00	0.00
Temperature Lag 2	0.00	0.00	0.00
Temperature Lag 3	0.00	0.00	0.00

Temperature Lag 4	0.01	0.00	0.05
Temperature Lag 5	0.03	0.00	0.00
Temperature Lag 6	0.00	0.00	0.03
Temperature Lag 7	0.03	0.09	0.00
Temperature Lag 8	0.00	0.00	0.00
Temperature Lag 9	0.00	0.00	0.00
Temperature Lag 10	0.00	0.00	0.00
Connectivity Lag 1	0.02	0.01	0.01
Connectivity Lag 2	0.00	0.00	0.00
Connectivity Lag 3	0.00	0.00	0.00
Connectivity Lag 4	0.00	0.00	0.00
Connectivity Lag 5	0.00	0.00	0.00
Connectivity Lag 6	0.00	0.00	0.00
Connectivity Lag 7	0.03	0.00	0.03
Connectivity Lag 8	0.00	0.00	0.00
Connectivity Lag 9	0.00	0.00	0.00
Connectivity Lag 10	0.00	0.00	0.00
Adjacency Lag 1	0.00	0.00	0.00
Adjacency Lag 2	0.00	0.00	0.00
Adjacency Lag 3	0.00	0.00	0.00
Adjacency Lag 4	0.00	0.00	0.00
Adjacency Lag 5	0.00	0.00	0.00
Adjacency Lag 6	0.00	0.00	0.00
Adjacency Lag 7	0.00	0.00	0.00
Adjacency Lag 8	0.00	0.00	0.00
Adjacency Lag 9	0.00	0.00	0.00
Adjacency Lag 10	0.00	0.00	0.00

	Kelantan Mean	Kelantan Max	Kelantan Min
Own Counts Lag 1	0.95	0.95	0.95
Own Counts Lag 2	0.00	0.00	0.00
Own Counts Lag 3	0.00	0.00	0.00
Own Counts Lag 4	0.00	0.00	0.00
Own Counts Lag 5	0.00	0.00	0.00
Own Counts Lag 6	-0.06	-0.06	-0.06
Own Counts Lag 7	0.00	0.00	0.00
Own Counts Lag 8	0.00	0.00	0.00
Own Counts Lag 9	0.00	0.00	0.00
Own Counts Lag 10	0.00	0.00	0.00
Absolute Humidity Lag 1	0.00	0.00	0.00
Absolute Humidity Lag 2	0.00	0.00	0.00
Absolute Humidity Lag 3	0.00	0.00	0.00
Absolute Humidity Lag 4	0.00	0.00	0.00
Absolute Humidity Lag 5	0.00	0.00	0.00
Absolute Humidity Lag 6	0.00	0.00	0.00
Absolute Humidity Lag 7	0.02	0.02	0.01
Absolute Humidity Lag 8	0.00	0.00	0.00
Absolute Humidity Lag 9	0.00	0.00	0.00
Absolute Humidity Lag 10	0.00	0.00	0.00
Relative Humidity Lag 1	0.00	0.00	0.00
Relative Humidity Lag 2	0.00	0.00	0.00
Relative Humidity Lag 3	0.00	0.00	0.00
Relative Humidity Lag 4	0.00	0.00	0.00
Relative Humidity Lag 5	0.00	0.00	0.00
Relative Humidity Lag 6	0.00	0.00	0.00
Relative Humidity Lag 7	0.00	0.00	0.00
Relative Humidity Lag 8	0.00	0.00	0.00
Relative Humidity Lag 9	0.00	0.00	0.00
Relative Humidity Lag 10	0.00	0.00	0.00
Total Precipitation Lag 1	0.00	-0.00	0.00
Total Precipitation Lag 2	0.00	0.00	0.00
Total Precipitation Lag 3	-0.01	-0.00	0.00
Total Precipitation Lag 4	0.00	0.00	0.00
Total Precipitation Lag 5	0.00	0.00	0.00
Total Precipitation Lag 6	0.00	0.00	0.00

Total Precipitation Lag 7	0.00	0.00	0.00
Total Precipitation Lag 8	0.00	0.00	0.00
Total Precipitation Lag 9	0.00	0.00	0.00
Total Precipitation Lag 10	0.00	0.00	0.00
Temperature Lag 1	0.00	0.00	0.00
Temperature Lag 2	0.00	0.00	0.00
Temperature Lag 3	0.00	0.00	0.00
Temperature Lag 4	0.00	0.00	0.00
Temperature Lag 5	0.00	0.00	0.00
Temperature Lag 6	0.00	0.00	0.00
Temperature Lag 7	0.00	0.00	0.00
Temperature Lag 8	0.00	0.00	0.00
Temperature Lag 9	0.00	0.00	0.00
Temperature Lag 10	0.00	0.00	0.00
Connectivity Lag 1	0.00	0.00	0.00
Connectivity Lag 2	0.00	0.00	0.00
Connectivity Lag 3	0.00	0.00	0.00
Connectivity Lag 4	0.01	0.01	0.01
Connectivity Lag 5	0.00	0.00	0.00
Connectivity Lag 6	0.00	0.00	0.00
Connectivity Lag 7	0.00	0.00	0.00
Connectivity Lag 8	0.00	0.00	0.00
Connectivity Lag 9	0.00	0.00	0.00
Connectivity Lag 10	0.00	0.00	0.00
Adjacency Lag 1	0.00	0.00	0.00
Adjacency Lag 2	0.00	0.00	0.00
Adjacency Lag 3	0.00	0.00	0.00
Adjacency Lag 4	0.00	0.00	0.00
Adjacency Lag 5	0.00	0.00	0.00
Adjacency Lag 6	0.00	0.00	0.00
Adjacency Lag 7	0.00	0.00	0.00
Adjacency Lag 8	0.00	0.00	0.00
Adjacency Lag 9	0.00	0.00	0.00
Adjacency Lag 10	0.00	0.00	0.00

	Melaka Mean	Melaka Max	Melaka Min
Own Counts Lag 1	0.60	0.59	0.61
Own Counts Lag 2	0.07	0.07	0.07
Own Counts Lag 3	0.05	0.05	0.04
Own Counts Lag 4	0.06	0.06	0.06
Own Counts Lag 5	0.00	0.00	0.01
Own Counts Lag 6	0.00	0.00	0.00
Own Counts Lag 7	0.00	0.00	0.00
Own Counts Lag 8	0.00	0.00	0.00
Own Counts Lag 9	0.00	0.00	0.00
Own Counts Lag 10	0.00	0.00	0.00
Absolute Humidity Lag 1	0.00	0.00	0.00
Absolute Humidity Lag 2	0.02	0.00	0.03
Absolute Humidity Lag 3	0.04	0.04	0.05
Absolute Humidity Lag 4	0.02	0.00	0.00
Absolute Humidity Lag 5	0.02	0.01	0.04
Absolute Humidity Lag 6	0.00	0.00	0.00
Absolute Humidity Lag 7	0.00	0.05	0.00
Absolute Humidity Lag 8	0.00	0.00	0.00
Absolute Humidity Lag 9	0.00	-0.02	0.00
Absolute Humidity Lag 10	0.00	0.00	0.00
Relative Humidity Lag 1	0.00	0.00	-0.02
Relative Humidity Lag 2	0.00	0.07	0.00
Relative Humidity Lag 3	0.00	0.00	0.00
Relative Humidity Lag 4	0.00	0.00	0.00
Relative Humidity Lag 5	0.00	0.01	0.00
Relative Humidity Lag 6	0.00	-0.03	-0.02
Relative Humidity Lag 7	0.00	0.00	0.00
Relative Humidity Lag 8	0.00	0.03	0.00
Relative Humidity Lag 9	0.00	0.00	-0.03

Relative Humidity Lag 10	0.00	0.00	-0.00
Total Precipitation Lag 1	0.00	0.00	0.13
Total Precipitation Lag 2	0.00	0.00	0.00
Total Precipitation Lag 3	0.00	0.00	0.00
Total Precipitation Lag 4	0.00	0.00	-0.00
Total Precipitation Lag 5	0.00	0.00	0.00
Total Precipitation Lag 6	0.00	0.02	-0.01
Total Precipitation Lag 7	0.00	0.00	0.00
Total Precipitation Lag 8	0.00	0.00	0.00
Total Precipitation Lag 9	0.00	0.01	0.00
Total Precipitation Lag 10	0.00	0.04	0.00
Temperature Lag 1	0.00	0.03	0.00
Temperature Lag 2	0.00	0.00	0.02
Temperature Lag 3	0.00	0.00	0.00
Temperature Lag 4	0.00	0.00	0.00
Temperature Lag 5	0.00	0.00	0.00
Temperature Lag 6	0.02	0.06	0.00
Temperature Lag 7	0.00	0.00	0.03
Temperature Lag 8	0.00	0.00	0.00
Temperature Lag 9	0.00	0.03	0.00
Temperature Lag 10	0.00	0.01	0.00
Connectivity Lag 1	0.00	0.00	0.00
Connectivity Lag 2	0.13	0.14	0.14
Connectivity Lag 3	0.06	0.06	0.04
Connectivity Lag 4	0.00	0.00	0.00
Connectivity Lag 5	0.00	0.00	0.00
Connectivity Lag 6	0.00	0.00	0.00
Connectivity Lag 7	0.00	0.00	0.00
Connectivity Lag 8	0.00	0.00	0.00
Connectivity Lag 9	0.00	0.00	0.00
Connectivity Lag 10	0.03	0.05	0.03
Adjacency Lag 1	0.00	0.00	0.00
Adjacency Lag 2	0.00	0.00	0.00
Adjacency Lag 3	0.00	0.00	0.00
Adjacency Lag 4	0.00	0.00	0.00
Adjacency Lag 5	0.00	0.00	0.00
Adjacency Lag 6	0.00	0.00	0.00
Adjacency Lag 7	0.00	0.00	0.00
Adjacency Lag 8	0.00	0.00	0.00
Adjacency Lag 9	0.00	0.00	0.00
Adjacency Lag 10	0.00	0.00	0.00

	Negeri Sembilan Mean	Negeri Sembilan Max	Negeri Sembilan Min
Own Counts Lag 1	0.48	0.47	0.48
Own Counts Lag 2	0.19	0.19	0.21
Own Counts Lag 3	0.00	0.00	0.00
Own Counts Lag 4	0.08	0.09	0.06
Own Counts Lag 5	0.03	0.03	0.03
Own Counts Lag 6	0.03	0.03	0.04
Own Counts Lag 7	0.00	0.00	0.00
Own Counts Lag 8	0.00	0.00	0.00
Own Counts Lag 9	0.00	0.00	0.00
Own Counts Lag 10	0.04	0.04	0.04
Absolute Humidity Lag 1	0.00	0.00	0.00
Absolute Humidity Lag 2	0.02	0.00	0.00
Absolute Humidity Lag 3	0.00	0.00	-0.01
Absolute Humidity Lag 4	0.00	0.00	-0.04
Absolute Humidity Lag 5	0.00	0.00	0.01
Absolute Humidity Lag 6	0.00	0.00	0.00
Absolute Humidity Lag 7	0.00	0.00	0.02
Absolute Humidity Lag 8	0.00	0.06	0.00
Absolute Humidity Lag 9	0.00	0.00	0.00
Absolute Humidity Lag 10	0.00	0.00	0.00
Relative Humidity Lag 1	0.00	0.00	0.00
Relative Humidity Lag 2	0.02	0.00	0.08

Relative Humidity Lag 3	0.00	0.00	0.00
Relative Humidity Lag 4	0.00	0.00	0.00
Relative Humidity Lag 5	0.00	0.02	0.00
Relative Humidity Lag 6	0.00	0.00	0.00
Relative Humidity Lag 7	0.00	0.00	0.00
Relative Humidity Lag 8	0.00	0.00	-0.04
Relative Humidity Lag 9	0.00	0.00	0.00
Relative Humidity Lag 10	0.00	0.00	0.00
Total Precipitation Lag 1	0.00	0.00	-0.01
Total Precipitation Lag 2	0.00	0.00	-0.07
Total Precipitation Lag 3	0.00	0.00	0.00
Total Precipitation Lag 4	0.00	0.00	0.00
Total Precipitation Lag 5	0.01	0.00	-0.01
Total Precipitation Lag 6	-0.01	0.00	-0.02
Total Precipitation Lag 7	0.00	0.00	0.00
Total Precipitation Lag 8	0.00	0.00	0.00
Total Precipitation Lag 9	0.00	0.00	0.00
Total Precipitation Lag 10	0.00	0.00	0.00
Temperature Lag 1	0.00	0.00	0.01
Temperature Lag 2	0.00	0.00	0.03
Temperature Lag 3	0.00	0.00	0.00
Temperature Lag 4	0.00	0.00	0.00
Temperature Lag 5	0.00	0.00	0.00
Temperature Lag 6	0.00	0.00	0.01
Temperature Lag 7	0.00	0.00	0.00
Temperature Lag 8	0.03	0.00	0.02
Temperature Lag 9	0.00	0.00	0.00
Temperature Lag 10	0.00	0.00	0.00
Connectivity Lag 1	0.00	0.00	0.00
Connectivity Lag 2	0.00	0.00	0.00
Connectivity Lag 3	0.00	0.00	0.00
Connectivity Lag 4	-0.01	-0.01	-0.02
Connectivity Lag 5	0.00	0.00	0.00
Connectivity Lag 6	0.00	0.00	0.00
Connectivity Lag 7	0.00	0.00	0.00
Connectivity Lag 8	0.00	0.00	0.00
Connectivity Lag 9	0.00	0.00	0.00
Connectivity Lag 10	0.00	0.00	0.00
Adjacency Lag 1	0.00	0.00	0.00
Adjacency Lag 2	0.00	0.00	-0.00
Adjacency Lag 3	0.00	0.00	0.00
Adjacency Lag 4	-0.01	0.00	-0.01
Adjacency Lag 5	0.00	0.00	0.00
Adjacency Lag 6	0.00	0.00	0.00
Adjacency Lag 7	0.00	0.00	0.00
Adjacency Lag 8	0.00	0.00	0.00
Adjacency Lag 9	0.00	0.00	0.00
Adjacency Lag 10	0.00	0.00	0.00

	Sabah Mean	Sabah Max	Sabah Min
Own Counts Lag 1	0.30	0.31	0.31
Own Counts Lag 2	0.06	0.06	0.06
Own Counts Lag 3	0.13	0.13	0.14
Own Counts Lag 4	0.02	0.02	0.02
Own Counts Lag 5	0.00	0.00	0.00
Own Counts Lag 6	0.00	0.00	0.00
Own Counts Lag 7	0.00	0.00	0.01
Own Counts Lag 8	0.01	0.01	0.04
Own Counts Lag 9	0.00	0.00	0.00
Own Counts Lag 10	0.00	0.00	0.00
Absolute Humidity Lag 1	0.00	0.00	0.00
Absolute Humidity Lag 2	0.00	0.00	0.00
Absolute Humidity Lag 3	0.00	0.00	0.00
Absolute Humidity Lag 4	0.00	0.00	0.00
Absolute Humidity Lag 5	0.00	0.00	0.00

Absolute Humidity Lag 6	0.00	0.00	0.00
Absolute Humidity Lag 7	0.00	0.00	0.00
Absolute Humidity Lag 8	0.00	0.00	0.00
Absolute Humidity Lag 9	0.00	0.00	0.00
Absolute Humidity Lag 10	0.00	0.00	0.00
Relative Humidity Lag 1	0.00	0.00	0.00
Relative Humidity Lag 2	0.00	0.00	0.00
Relative Humidity Lag 3	0.00	0.00	0.00
Relative Humidity Lag 4	0.00	0.00	0.00
Relative Humidity Lag 5	0.00	0.00	0.04
Relative Humidity Lag 6	0.00	0.00	0.00
Relative Humidity Lag 7	0.00	0.00	0.00
Relative Humidity Lag 8	0.02	0.00	0.04
Relative Humidity Lag 9	0.00	0.00	0.00
Relative Humidity Lag 10	0.00	0.00	0.00
Total Precipitation Lag 1	0.00	0.00	0.00
Total Precipitation Lag 2	0.00	0.00	-0.01
Total Precipitation Lag 3	0.00	0.00	0.00
Total Precipitation Lag 4	0.00	0.00	0.00
Total Precipitation Lag 5	0.00	0.00	0.00
Total Precipitation Lag 6	0.02	0.03	-0.08
Total Precipitation Lag 7	0.00	0.00	0.00
Total Precipitation Lag 8	0.00	0.00	0.00
Total Precipitation Lag 9	0.05	0.06	0.00
Total Precipitation Lag 10	0.00	0.00	0.00
Temperature Lag 1	-0.02	-0.03	0.00
Temperature Lag 2	0.00	0.00	0.09
Temperature Lag 3	0.00	0.00	0.00
Temperature Lag 4	0.00	0.00	0.00
Temperature Lag 5	0.00	0.00	0.00
Temperature Lag 6	0.00	0.00	0.00
Temperature Lag 7	0.00	-0.00	0.00
Temperature Lag 8	0.00	-0.00	0.00
Temperature Lag 9	0.00	0.00	0.00
Temperature Lag 10	0.00	0.00	0.00
Connectivity Lag 1	0.07	0.05	0.01
Connectivity Lag 2	0.07	0.08	0.08
Connectivity Lag 3	0.00	0.00	0.00
Connectivity Lag 4	0.00	0.00	0.00
Connectivity Lag 5	0.00	0.00	0.00
Connectivity Lag 6	0.00	0.00	0.00
Connectivity Lag 7	0.00	0.00	0.00
Connectivity Lag 8	0.00	0.00	0.00
Connectivity Lag 9	0.00	0.00	0.00
Connectivity Lag 10	0.00	0.00	0.00
Adjacency Lag 1	0.20	0.22	0.27
Adjacency Lag 2	0.00	0.00	0.00
Adjacency Lag 3	0.00	0.00	0.00
Adjacency Lag 4	0.00	0.00	0.00
Adjacency Lag 5	0.00	0.00	0.00
Adjacency Lag 6	0.00	0.00	0.00
Adjacency Lag 7	0.00	0.00	0.00
Adjacency Lag 8	0.00	0.00	0.00
Adjacency Lag 9	0.00	0.00	0.00
Adjacency Lag 10	-0.10	-0.11	-0.13

	Sarawak Mean	Sarawak Max	Sarawak Min
Own Counts Lag 1	0.63	0.63	0.62
Own Counts Lag 2	0.23	0.23	0.23
Own Counts Lag 3	0.00	0.00	0.00
Own Counts Lag 4	0.00	0.00	0.00
Own Counts Lag 5	0.04	0.05	0.05
Own Counts Lag 6	0.00	0.00	0.00
Own Counts Lag 7	0.00	0.00	0.00
Own Counts Lag 8	0.00	0.00	0.00

Own Counts Lag 9	0.00	0.00	0.00
Own Counts Lag 10	0.00	0.00	0.00
Absolute Humidity Lag 1	0.00	0.00	0.00
Absolute Humidity Lag 2	0.00	0.00	0.00
Absolute Humidity Lag 3	0.00	0.00	0.00
Absolute Humidity Lag 4	0.00	0.00	0.00
Absolute Humidity Lag 5	0.00	0.00	0.00
Absolute Humidity Lag 6	0.00	0.00	0.00
Absolute Humidity Lag 7	0.00	0.00	0.00
Absolute Humidity Lag 8	0.00	0.00	0.00
Absolute Humidity Lag 9	0.00	0.00	0.00
Absolute Humidity Lag 10	0.00	0.00	0.00
Relative Humidity Lag 1	0.03	0.07	0.04
Relative Humidity Lag 2	0.00	0.00	0.00
Relative Humidity Lag 3	0.00	0.02	0.00
Relative Humidity Lag 4	0.00	0.00	0.00
Relative Humidity Lag 5	0.00	0.00	0.00
Relative Humidity Lag 6	0.00	0.00	0.00
Relative Humidity Lag 7	0.00	0.04	0.00
Relative Humidity Lag 8	0.00	0.00	0.00
Relative Humidity Lag 9	0.00	0.00	-0.01
Relative Humidity Lag 10	0.00	0.00	0.00
Total Precipitation Lag 1	0.00	0.00	0.02
Total Precipitation Lag 2	0.01	0.02	0.00
Total Precipitation Lag 3	0.00	0.00	0.00
Total Precipitation Lag 4	0.00	0.00	0.00
Total Precipitation Lag 5	0.00	0.00	0.00
Total Precipitation Lag 6	0.00	0.00	0.00
Total Precipitation Lag 7	0.00	0.00	0.05
Total Precipitation Lag 8	0.00	0.00	0.00
Total Precipitation Lag 9	0.00	0.00	0.00
Total Precipitation Lag 10	0.00	-0.02	0.00
Temperature Lag 1	0.00	0.00	0.00
Temperature Lag 2	0.00	0.00	0.00
Temperature Lag 3	0.00	0.00	0.00
Temperature Lag 4	0.00	0.00	0.00
Temperature Lag 5	0.00	0.00	0.00
Temperature Lag 6	0.00	0.03	0.00
Temperature Lag 7	0.00	0.00	0.00
Temperature Lag 8	0.00	0.00	0.00
Temperature Lag 9	0.00	0.00	0.00
Temperature Lag 10	0.00	0.00	0.00
Connectivity Lag 1	0.03	0.00	0.03
Connectivity Lag 2	0.00	0.00	0.00
Connectivity Lag 3	0.00	0.00	0.00
Connectivity Lag 4	0.00	0.00	0.00
Connectivity Lag 5	0.00	0.00	0.00
Connectivity Lag 6	0.00	0.00	0.00
Connectivity Lag 7	0.00	0.00	0.00
Connectivity Lag 8	0.00	0.00	0.00
Connectivity Lag 9	0.00	0.00	0.00
Connectivity Lag 10	0.00	0.00	0.00
Adjacency Lag 1	0.00	0.02	0.01
Adjacency Lag 2	0.00	0.00	0.00
Adjacency Lag 3	0.00	0.00	0.00
Adjacency Lag 4	0.00	0.00	0.00
Adjacency Lag 5	0.00	0.00	0.00
Adjacency Lag 6	0.00	0.00	0.00
Adjacency Lag 7	0.00	0.01	0.00
Adjacency Lag 8	0.00	0.02	0.01
Adjacency Lag 9	0.00	0.00	0.00
Adjacency Lag 10	0.00	0.00	0.00

	Selangor Mean	Selangor Max	Selangor Min

Own Counts Lag 1	0.62	0.62	0.62
Own Counts Lag 2	0.25	0.25	0.24
Own Counts Lag 3	0.04	0.03	0.04
Own Counts Lag 4	0.00	0.00	0.00
Own Counts Lag 5	0.00	0.00	0.00
Own Counts Lag 6	0.00	0.00	0.00
Own Counts Lag 7	0.00	0.00	0.00
Own Counts Lag 8	0.00	0.00	0.00
Own Counts Lag 9	0.00	0.00	0.00
Own Counts Lag 10	0.00	0.00	0.00
Absolute Humidity Lag 1	0.03	0.02	0.01
Absolute Humidity Lag 2	0.00	0.00	0.00
Absolute Humidity Lag 3	0.00	0.00	0.00
Absolute Humidity Lag 4	0.00	0.00	0.02
Absolute Humidity Lag 5	0.00	0.00	0.00
Absolute Humidity Lag 6	0.01	0.01	0.03
Absolute Humidity Lag 7	0.06	0.04	0.01
Absolute Humidity Lag 8	0.00	0.01	0.00
Absolute Humidity Lag 9	0.00	0.00	0.00
Absolute Humidity Lag 10	0.00	0.00	0.00
Relative Humidity Lag 1	0.00	0.00	0.00
Relative Humidity Lag 2	0.00	0.02	0.00
Relative Humidity Lag 3	0.00	0.00	0.00
Relative Humidity Lag 4	0.00	0.00	0.00
Relative Humidity Lag 5	0.00	0.00	0.00
Relative Humidity Lag 6	0.00	0.00	0.00
Relative Humidity Lag 7	0.00	0.00	0.01
Relative Humidity Lag 8	0.00	0.00	0.00
Relative Humidity Lag 9	0.00	0.00	0.00
Relative Humidity Lag 10	0.00	0.00	-0.03
Total Precipitation Lag 1	0.00	0.02	0.00
Total Precipitation Lag 2	0.00	0.00	0.00
Total Precipitation Lag 3	0.00	0.00	0.00
Total Precipitation Lag 4	0.03	0.03	0.06
Total Precipitation Lag 5	0.00	-0.02	0.00
Total Precipitation Lag 6	0.00	-0.01	0.00
Total Precipitation Lag 7	0.00	0.00	0.00
Total Precipitation Lag 8	0.00	0.00	0.00
Total Precipitation Lag 9	0.00	0.00	0.00
Total Precipitation Lag 10	-0.03	-0.05	0.00
Temperature Lag 1	0.00	0.00	0.00
Temperature Lag 2	0.00	0.00	0.00
Temperature Lag 3	0.00	0.00	0.00
Temperature Lag 4	0.00	0.00	0.00
Temperature Lag 5	0.00	0.00	0.00
Temperature Lag 6	0.00	0.00	0.00
Temperature Lag 7	0.00	0.00	0.00
Temperature Lag 8	0.00	0.00	0.01
Temperature Lag 9	0.00	0.00	0.00
Temperature Lag 10	0.00	0.01	0.00
Connectivity Lag 1	0.00	0.00	0.00
Connectivity Lag 2	0.00	0.00	0.00
Connectivity Lag 3	0.00	0.00	0.00
Connectivity Lag 4	0.00	0.00	0.00
Connectivity Lag 5	0.00	0.00	0.00
Connectivity Lag 6	0.00	0.00	0.00
Connectivity Lag 7	0.00	0.00	0.00
Connectivity Lag 8	0.00	0.00	0.00
Connectivity Lag 9	0.00	0.00	0.00
Connectivity Lag 10	0.00	0.00	0.00
Adjacency Lag 1	0.00	0.00	0.00
Adjacency Lag 2	0.00	0.00	0.00
Adjacency Lag 3	0.00	0.00	0.00
Adjacency Lag 4	0.00	0.00	0.00
Adjacency Lag 5	0.00	0.00	0.00

Adjacency Lag 6	0.00	0.02	0.00
Adjacency Lag 7	0.00	0.00	0.00
Adjacency Lag 8	0.00	0.00	0.00
Adjacency Lag 9	0.00	0.00	0.00
Adjacency Lag 10	0.00	0.00	0.00

	Terengganu Mean	Terengganu Max	Terengganu Min
Own Counts Lag 1	0.65	0.65	0.65
Own Counts Lag 2	0.20	0.20	0.20
Own Counts Lag 3	0.03	0.04	0.03
Own Counts Lag 4	0.00	0.00	0.00
Own Counts Lag 5	0.00	0.00	0.00
Own Counts Lag 6	0.00	0.00	0.00
Own Counts Lag 7	0.00	0.00	0.00
Own Counts Lag 8	0.00	0.00	0.00
Own Counts Lag 9	0.00	0.00	0.00
Own Counts Lag 10	0.00	0.00	0.00
Absolute Humidity Lag 1	0.00	0.00	0.00
Absolute Humidity Lag 2	0.00	0.00	0.00
Absolute Humidity Lag 3	0.00	0.00	0.00
Absolute Humidity Lag 4	0.00	0.00	0.00
Absolute Humidity Lag 5	0.00	0.00	0.00
Absolute Humidity Lag 6	0.00	0.00	0.00
Absolute Humidity Lag 7	0.00	0.00	0.00
Absolute Humidity Lag 8	0.00	0.00	0.00
Absolute Humidity Lag 9	0.00	0.00	0.00
Absolute Humidity Lag 10	0.00	0.00	0.00
Relative Humidity Lag 1	0.00	0.00	0.00
Relative Humidity Lag 2	0.00	0.00	0.00
Relative Humidity Lag 3	0.00	0.00	0.00
Relative Humidity Lag 4	0.00	0.00	0.00
Relative Humidity Lag 5	0.00	0.00	0.00
Relative Humidity Lag 6	0.00	0.00	0.00
Relative Humidity Lag 7	0.00	0.00	0.00
Relative Humidity Lag 8	-0.00	-0.02	0.00
Relative Humidity Lag 9	0.00	0.00	0.00
Relative Humidity Lag 10	0.00	0.00	0.00
Total Precipitation Lag 1	0.00	0.00	0.00
Total Precipitation Lag 2	0.00	0.00	0.00
Total Precipitation Lag 3	0.00	0.00	0.00
Total Precipitation Lag 4	0.00	0.00	0.00
Total Precipitation Lag 5	0.00	0.00	0.00
Total Precipitation Lag 6	0.00	0.00	0.00
Total Precipitation Lag 7	0.00	0.00	0.00
Total Precipitation Lag 8	0.00	0.00	0.00
Total Precipitation Lag 9	0.00	0.00	0.00
Total Precipitation Lag 10	0.00	0.00	0.00
Temperature Lag 1	0.00	0.00	0.00
Temperature Lag 2	0.00	0.00	0.00
Temperature Lag 3	0.00	0.00	0.00
Temperature Lag 4	0.00	0.00	0.00
Temperature Lag 5	0.00	0.00	0.00
Temperature Lag 6	0.00	0.00	0.00
Temperature Lag 7	0.00	0.00	0.00
Temperature Lag 8	0.00	0.00	0.00
Temperature Lag 9	0.00	0.00	0.00
Temperature Lag 10	0.00	0.00	0.00
Connectivity Lag 1	0.00	0.00	0.00
Connectivity Lag 2	0.00	0.00	0.00
Connectivity Lag 3	0.00	0.00	0.00
Connectivity Lag 4	0.00	0.00	0.00
Connectivity Lag 5	0.00	0.00	0.00
Connectivity Lag 6	0.00	0.00	0.00
Connectivity Lag 7	0.00	0.00	0.00
Connectivity Lag 8	0.00	0.00	0.00

Connectivity Lag 9	0.00	0.00	0.00
Connectivity Lag 10	0.00	0.00	0.00
Adjacency Lag 1	0.00	0.00	0.00
Adjacency Lag 2	0.00	0.00	0.00
Adjacency Lag 3	0.00	0.00	0.00
Adjacency Lag 4	0.00	0.00	0.00
Adjacency Lag 5	0.00	0.00	0.00
Adjacency Lag 6	0.00	0.00	0.00
Adjacency Lag 7	0.00	0.00	0.00
Adjacency Lag 8	0.00	0.00	0.00
Adjacency Lag 9	0.00	0.00	0.00
Adjacency Lag 10	0.00	0.00	0.00

	Kuala Lumpur Mean	Kuala Lumpur Max	Kuala Lumpur Min
Own Counts Lag 1	0.56	0.57	0.58
Own Counts Lag 2	0.09	0.09	0.08
Own Counts Lag 3	0.23	0.22	0.20
Own Counts Lag 4	0.00	0.00	0.00
Own Counts Lag 5	0.00	0.00	0.00
Own Counts Lag 6	0.00	0.00	0.00
Own Counts Lag 7	0.00	0.00	0.00
Own Counts Lag 8	0.00	0.00	0.00
Own Counts Lag 9	0.00	0.00	0.00
Own Counts Lag 10	0.00	0.00	0.00
Absolute Humidity Lag 1	0.00	0.00	0.00
Absolute Humidity Lag 2	0.00	0.00	0.00
Absolute Humidity Lag 3	0.00	0.00	0.00
Absolute Humidity Lag 4	0.03	0.03	0.02
Absolute Humidity Lag 5	0.02	0.00	0.02
Absolute Humidity Lag 6	0.00	0.00	0.00
Absolute Humidity Lag 7	0.00	0.00	0.00
Absolute Humidity Lag 8	0.01	0.05	0.00
Absolute Humidity Lag 9	0.00	0.00	0.00
Absolute Humidity Lag 10	-0.01	-0.02	-0.01
Relative Humidity Lag 1	0.00	0.00	0.00
Relative Humidity Lag 2	0.00	0.01	0.01
Relative Humidity Lag 3	0.00	0.00	0.01
Relative Humidity Lag 4	0.04	0.03	0.04
Relative Humidity Lag 5	0.00	0.00	0.00
Relative Humidity Lag 6	0.00	0.00	0.00
Relative Humidity Lag 7	0.01	0.03	0.00
Relative Humidity Lag 8	0.00	0.00	0.00
Relative Humidity Lag 9	0.00	0.00	0.00
Relative Humidity Lag 10	-0.00	-0.01	0.00
Total Precipitation Lag 1	0.01	0.00	0.00
Total Precipitation Lag 2	0.00	0.00	0.00
Total Precipitation Lag 3	0.00	0.00	0.00
Total Precipitation Lag 4	0.00	0.01	0.00
Total Precipitation Lag 5	0.00	0.00	0.00
Total Precipitation Lag 6	0.02	0.02	0.01
Total Precipitation Lag 7	0.00	0.00	0.04
Total Precipitation Lag 8	0.00	0.00	0.00
Total Precipitation Lag 9	0.00	0.00	0.00
Total Precipitation Lag 10	0.00	0.00	0.00
Temperature Lag 1	0.00	-0.00	0.00
Temperature Lag 2	0.00	0.00	0.00
Temperature Lag 3	0.00	-0.00	0.00
Temperature Lag 4	0.00	0.00	0.00
Temperature Lag 5	0.00	0.00	0.00
Temperature Lag 6	0.00	0.00	0.00
Temperature Lag 7	0.00	0.00	0.00
Temperature Lag 8	0.00	0.00	0.00
Temperature Lag 9	0.00	0.00	0.00
Temperature Lag 10	0.00	0.00	0.00
Connectivity Lag 1	0.00	0.00	0.00

Connectivity Lag 2	0.00	0.00	0.00
Connectivity Lag 3	0.00	0.00	0.00
Connectivity Lag 4	0.00	0.00	0.00
Connectivity Lag 5	0.00	0.00	0.00
Connectivity Lag 6	0.00	0.00	0.00
Connectivity Lag 7	-0.03	-0.03	0.00
Connectivity Lag 8	-0.00	-0.00	0.00
Connectivity Lag 9	0.00	0.00	0.00
Connectivity Lag 10	0.00	0.00	0.00
Adjacency Lag 1	0.12	0.12	0.11
Adjacency Lag 2	0.00	0.00	0.00
Adjacency Lag 3	0.00	0.00	0.00
Adjacency Lag 4	0.00	0.00	0.00
Adjacency Lag 5	0.00	0.00	0.00
Adjacency Lag 6	0.00	0.00	0.00
Adjacency Lag 7	0.00	0.00	0.00
Adjacency Lag 8	0.00	0.00	0.00
Adjacency Lag 9	0.00	0.00	0.00
Adjacency Lag 10	0.00	0.00	0.00

	Labuan Mean	Labuan Max	Labuan Min
Own Counts Lag 1	0.94	0.94	0.94
Own Counts Lag 2	0.00	0.00	0.00
Own Counts Lag 3	0.00	0.00	0.00
Own Counts Lag 4	0.00	0.00	0.00
Own Counts Lag 5	0.00	0.00	0.00
Own Counts Lag 6	0.00	0.00	0.00
Own Counts Lag 7	0.00	0.00	0.00
Own Counts Lag 8	0.00	0.00	0.00
Own Counts Lag 9	0.00	0.00	0.00
Own Counts Lag 10	0.00	0.00	0.00
Absolute Humidity Lag 1	0.00	0.01	0.00
Absolute Humidity Lag 2	0.00	0.00	0.00
Absolute Humidity Lag 3	0.00	0.00	0.00
Absolute Humidity Lag 4	0.00	0.00	0.00
Absolute Humidity Lag 5	0.00	0.00	0.01
Absolute Humidity Lag 6	0.00	0.00	0.00
Absolute Humidity Lag 7	0.00	0.00	0.00
Absolute Humidity Lag 8	0.00	0.00	0.00
Absolute Humidity Lag 9	0.00	0.00	0.00
Absolute Humidity Lag 10	0.00	0.00	0.00
Relative Humidity Lag 1	0.00	0.01	0.00
Relative Humidity Lag 2	0.00	0.00	0.00
Relative Humidity Lag 3	0.00	0.00	0.00
Relative Humidity Lag 4	0.00	0.00	0.00
Relative Humidity Lag 5	0.00	0.00	0.00
Relative Humidity Lag 6	0.00	0.00	0.00
Relative Humidity Lag 7	0.00	0.00	0.01
Relative Humidity Lag 8	0.00	0.00	0.00
Relative Humidity Lag 9	0.00	0.00	0.00
Relative Humidity Lag 10	0.00	0.00	0.00
Total Precipitation Lag 1	0.00	0.00	0.00
Total Precipitation Lag 2	0.00	0.00	0.00
Total Precipitation Lag 3	0.00	0.00	0.00
Total Precipitation Lag 4	0.00	0.00	0.00
Total Precipitation Lag 5	0.00	0.00	0.00
Total Precipitation Lag 6	0.00	0.00	0.00
Total Precipitation Lag 7	0.00	0.00	0.00
Total Precipitation Lag 8	0.00	0.00	0.00
Total Precipitation Lag 9	0.00	0.00	0.00
Total Precipitation Lag 10	0.00	0.00	0.00
Temperature Lag 1	0.00	0.00	0.00
Temperature Lag 2	0.00	0.00	0.00
Temperature Lag 3	0.00	0.00	0.00
Temperature Lag 4	0.00	0.00	0.00

Temperature Lag 5	0.00	0.00	0.00
Temperature Lag 6	0.00	0.00	0.00
Temperature Lag 7	0.00	0.00	0.00
Temperature Lag 8	0.00	0.00	0.00
Temperature Lag 9	0.00	0.00	0.00
Temperature Lag 10	0.00	0.00	0.00
Connectivity Lag 1	0.00	0.00	0.00
Connectivity Lag 2	0.00	0.00	0.00
Connectivity Lag 3	0.00	0.00	0.00
Connectivity Lag 4	0.00	0.00	0.00
Connectivity Lag 5	0.00	0.00	0.00
Connectivity Lag 6	0.00	0.00	0.00
Connectivity Lag 7	0.00	0.00	0.00
Connectivity Lag 8	0.00	0.00	0.00
Connectivity Lag 9	0.00	0.00	0.00
Connectivity Lag 10	0.00	0.00	0.00
Adjacency Lag 1	0.00	0.00	0.00
Adjacency Lag 2	0.00	0.00	0.00
Adjacency Lag 3	0.00	0.00	0.00
Adjacency Lag 4	0.00	0.00	0.00
Adjacency Lag 5	0.00	0.00	0.00
Adjacency Lag 6	0.00	0.00	0.00
Adjacency Lag 7	0.00	0.00	0.00
Adjacency Lag 8	0.00	0.00	0.00
Adjacency Lag 9	0.00	0.00	0.00
Adjacency Lag 10	0.00	0.00	0.00

	Singapore Mean	Singapore Max	Singapore Min
Own Counts Lag 1	0.84	0.84	0.83
Own Counts Lag 2	0.01	0.02	0.00
Own Counts Lag 3	0.00	0.00	0.00
Own Counts Lag 4	0.00	0.00	0.00
Own Counts Lag 5	0.00	0.00	0.00
Own Counts Lag 6	0.00	0.00	0.00
Own Counts Lag 7	0.00	0.00	0.00
Own Counts Lag 8	0.00	0.00	0.00
Own Counts Lag 9	0.00	0.00	0.00
Own Counts Lag 10	0.00	0.00	0.00
Absolute Humidity Lag 1	0.00	0.00	0.00
Absolute Humidity Lag 2	0.00	0.00	0.00
Absolute Humidity Lag 3	0.00	0.00	0.00
Absolute Humidity Lag 4	0.00	0.00	0.00
Absolute Humidity Lag 5	0.00	0.00	0.00
Absolute Humidity Lag 6	0.00	0.00	0.00
Absolute Humidity Lag 7	0.01	0.01	0.00
Absolute Humidity Lag 8	0.00	0.00	0.00
Absolute Humidity Lag 9	0.00	0.00	0.00
Absolute Humidity Lag 10	0.00	0.00	0.00
Relative Humidity Lag 1	0.00	0.00	0.00
Relative Humidity Lag 2	0.00	0.00	0.00
Relative Humidity Lag 3	0.01	0.03	0.00
Relative Humidity Lag 4	0.00	0.00	0.00
Relative Humidity Lag 5	0.00	0.00	0.00
Relative Humidity Lag 6	0.01	0.00	0.00
Relative Humidity Lag 7	0.00	0.01	0.00
Relative Humidity Lag 8	0.00	0.00	0.00
Relative Humidity Lag 9	0.00	0.00	0.00
Relative Humidity Lag 10	0.00	0.00	0.00
Total Precipitation Lag 1	0.01	0.01	0.00
Total Precipitation Lag 2	0.01	0.00	0.00
Total Precipitation Lag 3	0.00	0.00	0.00
Total Precipitation Lag 4	0.00	0.00	0.02
Total Precipitation Lag 5	0.00	0.00	0.00
Total Precipitation Lag 6	0.00	0.00	0.00
Total Precipitation Lag 7	0.00	0.00	0.00

Total Precipitation Lag 8	0.00	0.00	0.00
Total Precipitation Lag 9	0.00	0.00	0.00
Total Precipitation Lag 10	0.00	0.00	0.00
Temperature Lag 1	0.00	0.00	0.00
Temperature Lag 2	0.00	0.00	0.00
Temperature Lag 3	0.00	0.00	0.00
Temperature Lag 4	0.00	0.00	0.00
Temperature Lag 5	0.00	0.00	0.00
Temperature Lag 6	0.00	0.00	0.00
Temperature Lag 7	0.00	0.00	0.00
Temperature Lag 8	0.00	0.00	0.00
Temperature Lag 9	0.00	0.00	0.00
Temperature Lag 10	0.00	0.00	0.00
Connectivity Lag 1	0.04	0.04	0.02
Connectivity Lag 2	0.00	0.00	0.00
Connectivity Lag 3	0.00	0.00	0.00
Connectivity Lag 4	0.00	0.00	0.00
Connectivity Lag 5	0.00	0.00	0.00
Connectivity Lag 6	0.00	0.00	0.00
Connectivity Lag 7	0.00	0.00	0.00
Connectivity Lag 8	0.00	0.00	0.00
Connectivity Lag 9	0.00	0.00	0.00
Connectivity Lag 10	0.00	0.00	0.00
Adjacency Lag 1	0.00	0.00	0.00
Adjacency Lag 2	0.00	0.00	0.00
Adjacency Lag 3	0.00	0.00	0.00
Adjacency Lag 4	0.03	0.03	0.01
Adjacency Lag 5	0.00	0.00	0.00
Adjacency Lag 6	0.00	0.00	0.00
Adjacency Lag 7	0.00	0.00	0.00
Adjacency Lag 8	0.00	0.00	0.00
Adjacency Lag 9	0.00	0.00	0.00
Adjacency Lag 10	0.00	0.00	0.00

References

[1] Jianqing Fan, Shaojun Guo, and Ning Hao. Variance estimation using refitted cross-validation in ultrahigh dimensional regression. *Journal of the Royal Statistical Society: Series B (Statistical Methodology)*, 74(1):37–65, 2012.

[2] Helmut Lütkepohl. *New introduction to multiple time series analysis*. Springer Science & Business Media, 2005.

[3] H Hashem Pesaran and Yongcheol Shin. Generalized impulse response analysis in linear multivariate models. *Economics letters*, 58(1):17–29, 1998.
