# Supplementary material for: Explicit characterization of human population connectivity reveals long run persistence of interregional dengue shocks
Source: J R Soc Interface. 2020 Jul 22;17(168):20200340. doi: 10.1098/rsif.2020.0340 (PMC7423435; doi:10.1098/rsif.2020.0340)
Supplement: Technical Appendix 2 [file rsif20200340supp2.pdf]

Technical Appendix 2

June 23, 2020

Contents

1 Forecast Error Impulse Response Functions for SSTAR **2**

2 Generalized Impulse Response Functions for SSTAR **18**

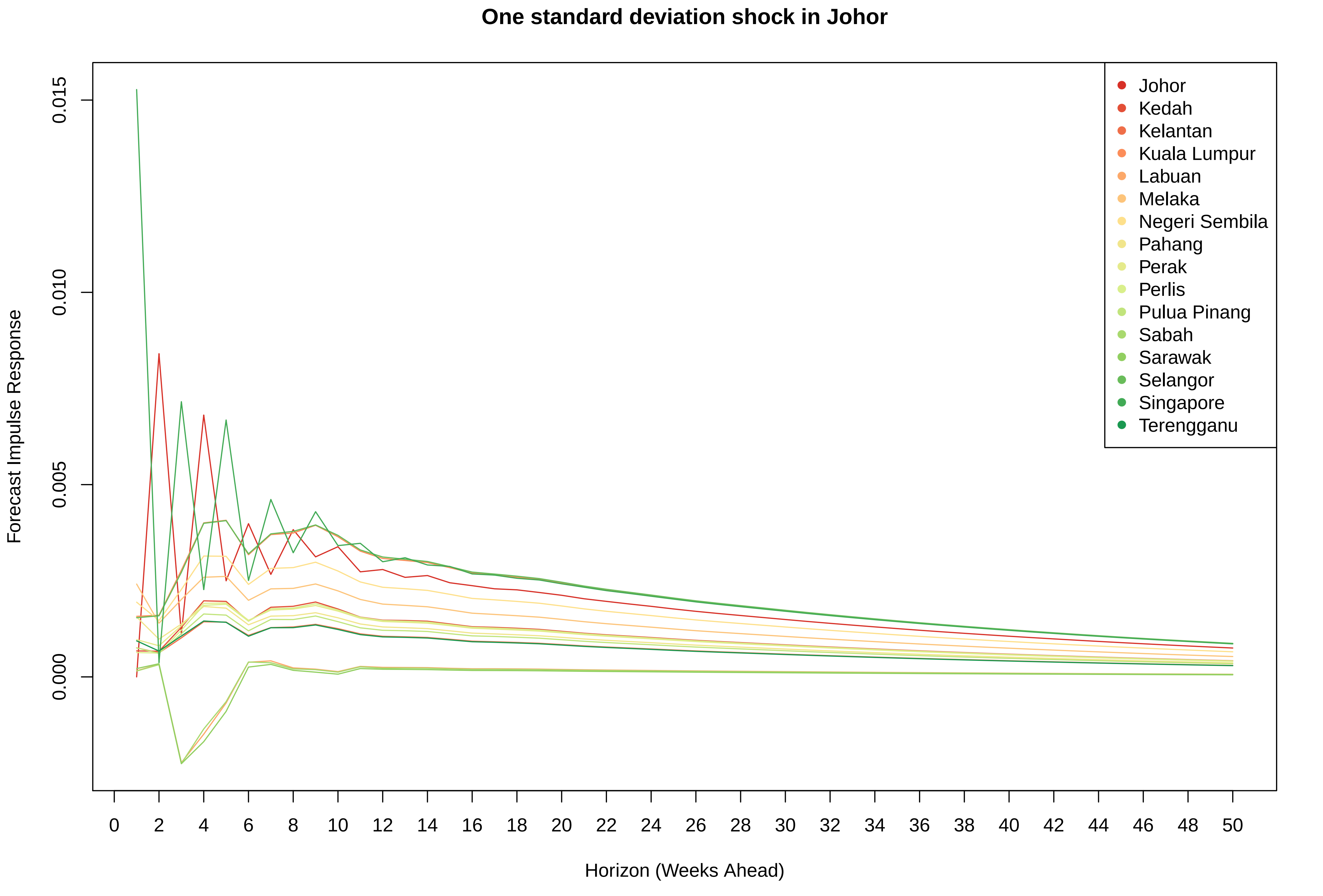

One standard deviation shock in Johor
Forecast Impulse Response
Horizon (Weeks Ahead)
0.000
0.005
0.010
0.015
0
2
4
6
8
10
12
14
16
18
20
22
24
26
28
30
32
34
36
38
40
42
44
46
48
50
Johor
Kedah
Kelantan
Kuala Lumpur
Labuan
Melaka
Negeri Sembila
Pahang
Perak
Perlis
Pulua Pinang
Sabah
Sarawak
Selangor
Singapore
Terengganu

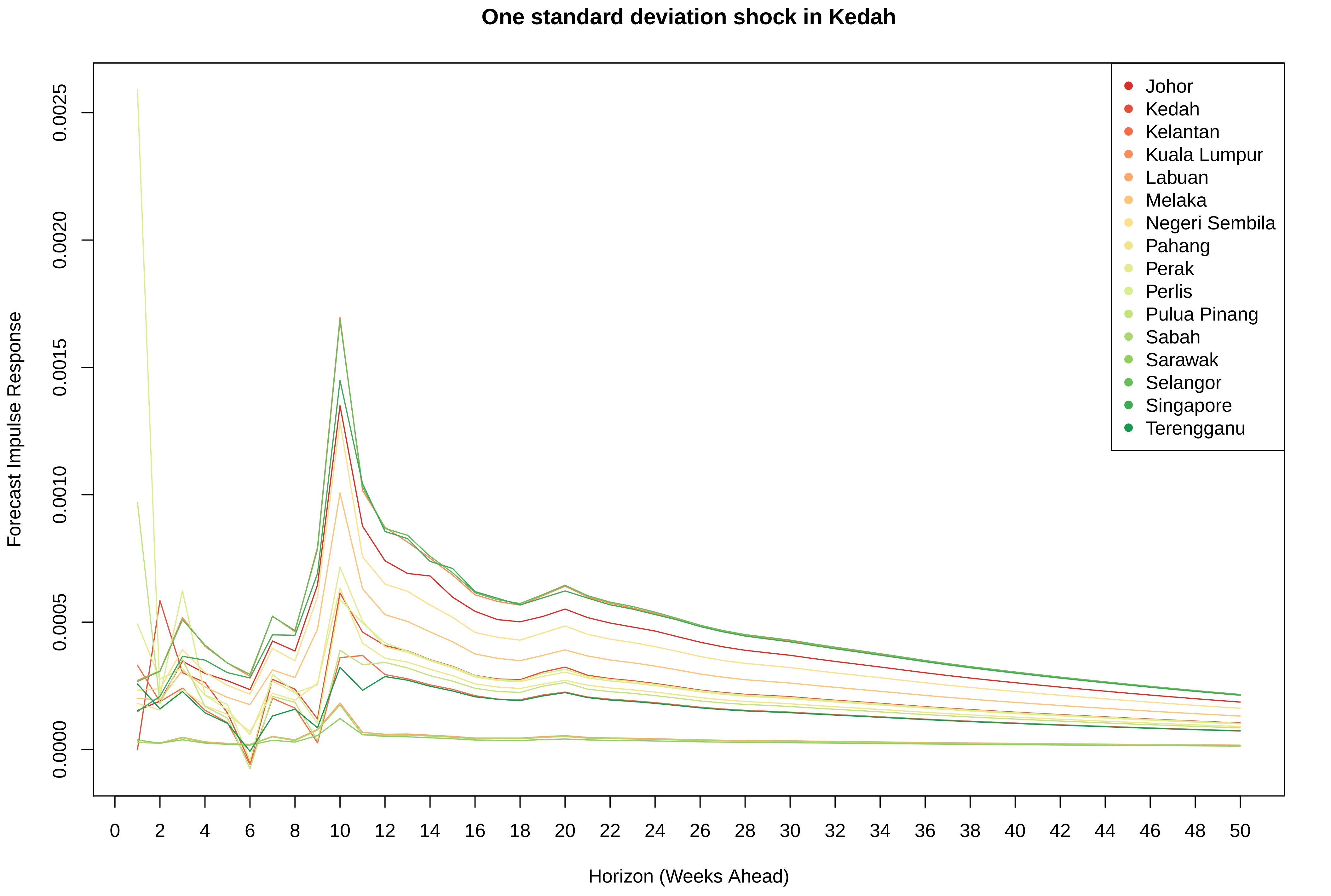
One standard deviation shock in Kedah
Forecast Impulse Response
Horizon (Weeks Ahead)
0.0000
0.0005
0.0010
0.0015
0.0020
0.0025
0
2
4
6
8
10
12
14
16
18
20
22
24
26
28
30
32
34
36
38
40
42
44
46
48
50
Johor
Kedah
Kelantan
Kuala Lumpur
Labuan
Melaka
Negeri Sembila
Pahang
Perak
Perlis
Pulua Pinang
Sabah
Sarawak
Selangor
Singapore
Terengganu

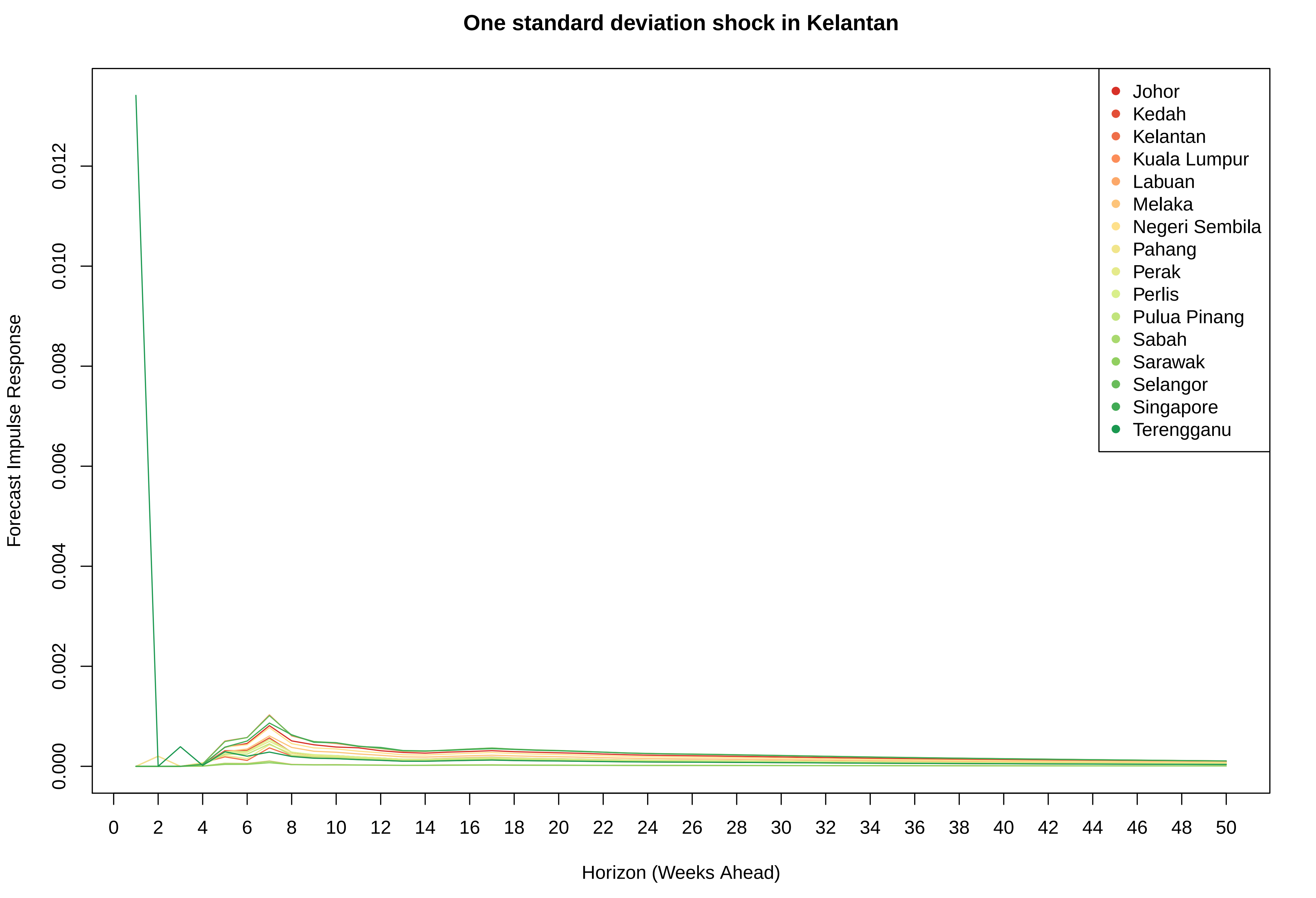
One standard deviation shock in Kelantan
Forecast Impulse Response
0.000
0.002
0.004
0.006
0.008
0.010
0.012
Horizon (Weeks Ahead)
0
2
4
6
8
10
12
14
16
18
20
22
24
26
28
30
32
34
36
38
40
42
44
46
48
50
Johor
Kedah
Kelantan
Kuala Lumpur
Labuan
Melaka
Negeri Sembila
Pahang
Perak
Perlis
Pulua Pinang
Sabah
Sarawak
Selangor
Singapore
Terengganu

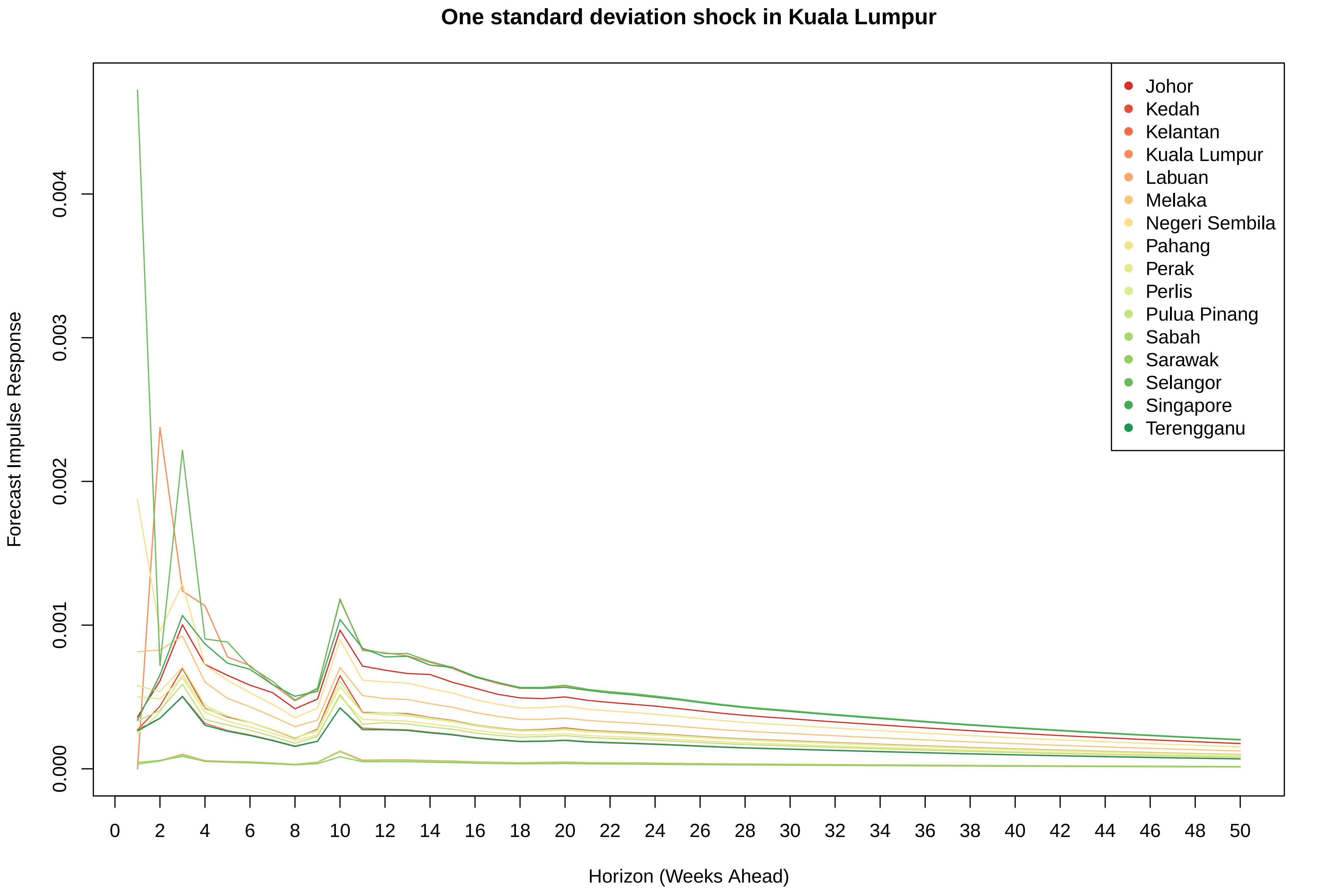

One standard deviation shock in Kuala Lumpur
Forecast Impulse Response
0.000
0.001
0.002
0.003
0.004
Horizon (Weeks Ahead)
0
2
4
6
8
10
12
14
16
18
20
22
24
26
28
30
32
34
36
38
40
42
44
46
48
50
Johor
Kedah
Kelantan
Kuala Lumpur
Labuan
Melaka
Negeri Sembila
Pahang
Perak
Perlis
Pulua Pinang
Sabah
Sarawak
Selangor
Singapore
Terengganu

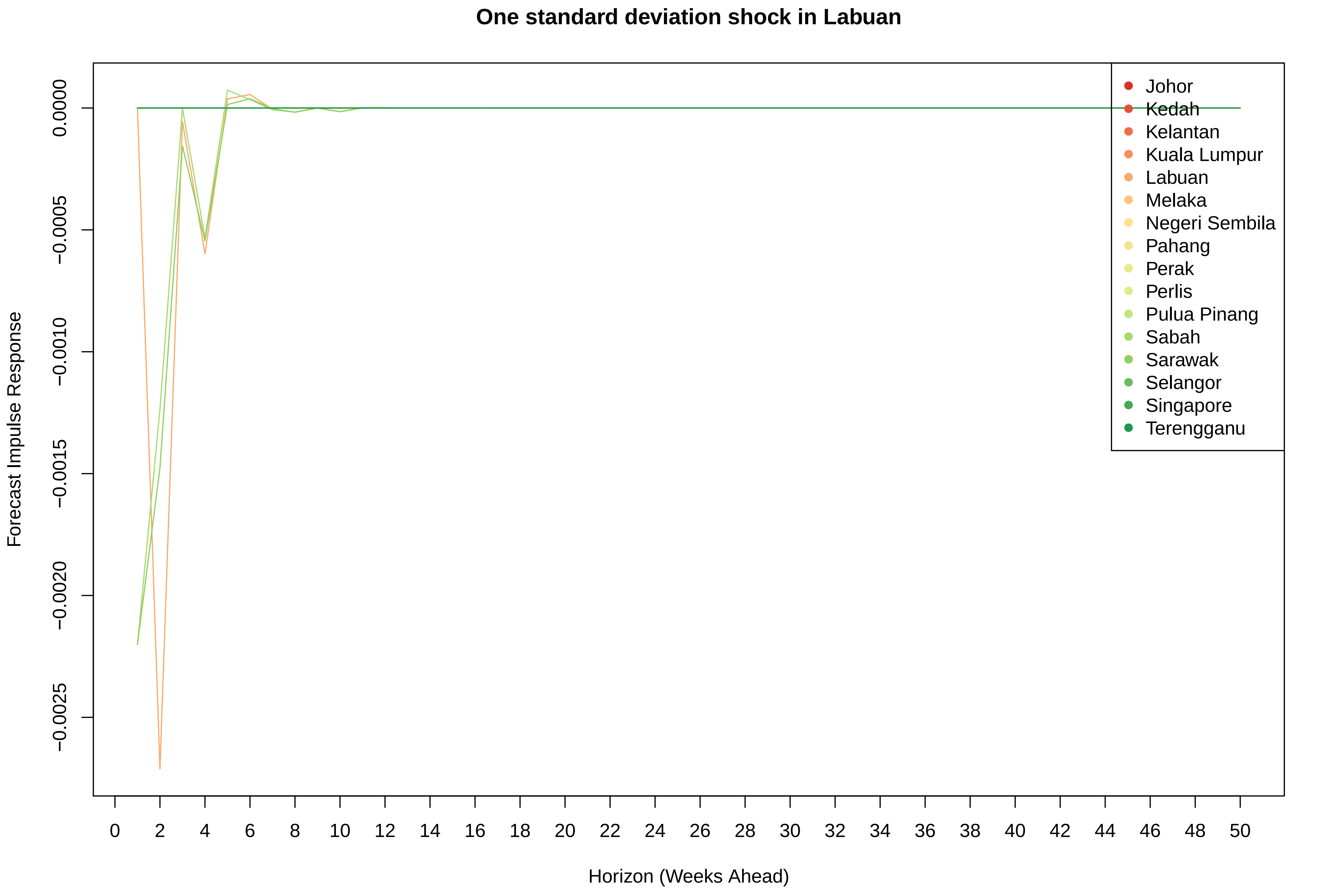

One standard deviation shock in Labuan
Forecast Impulse Response
0.0000
−0.0005
−0.0010
−0.0015
−0.0020
−0.0025
Horizon (Weeks Ahead)
0
2
4
6
8
10
12
14
16
18
20
22
24
26
28
30
32
34
36
38
40
42
44
46
48
50
Johor
Kedah
Kelantan
Kuala Lumpur
Labuan
Melaka
Negeri Sembila
Pahang
Perak
Perlis
Pulua Pinang
Sabah
Sarawak
Selangor
Singapore
Terengganu

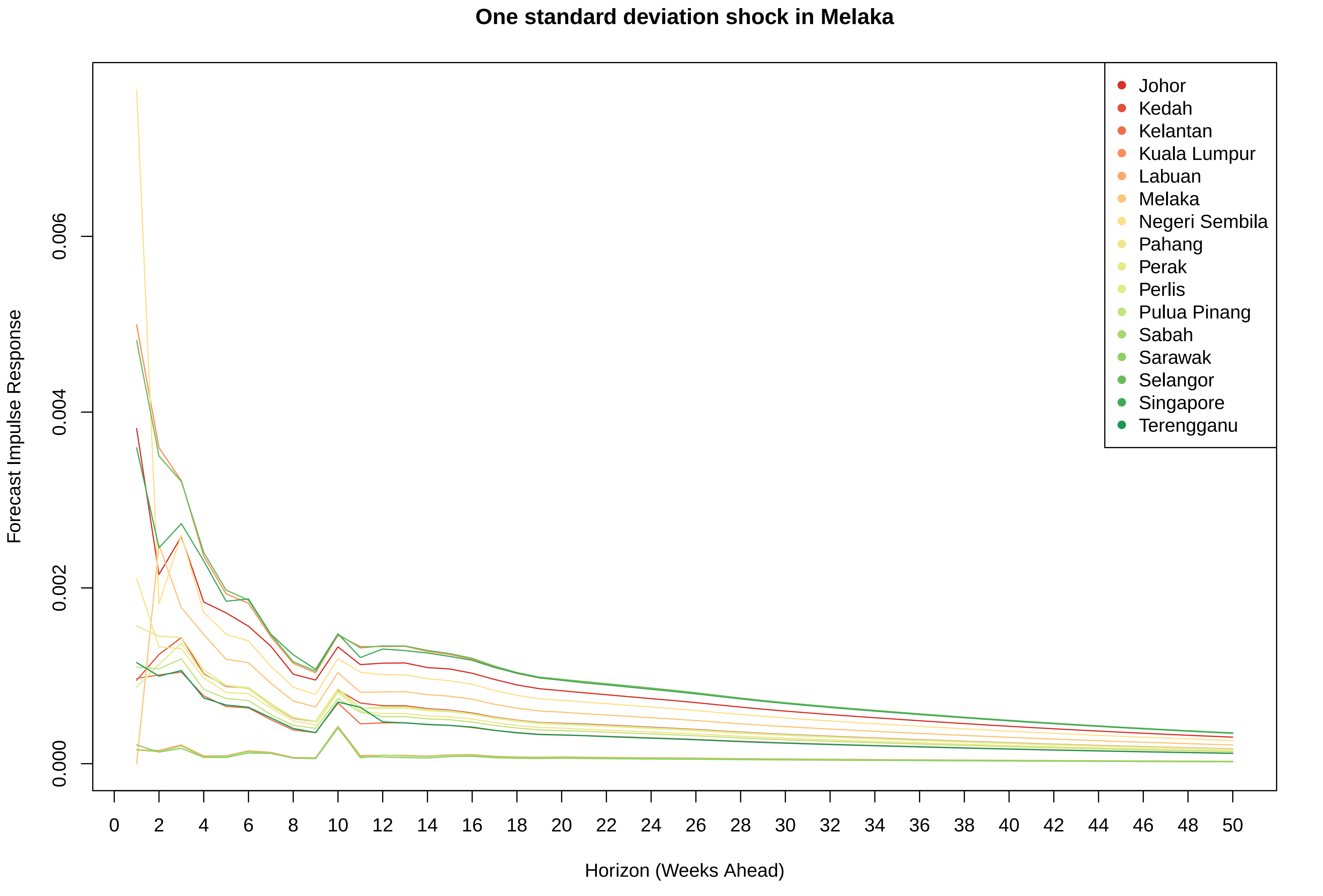
One standard deviation shock in Melaka
Forecast Impulse Response
Horizon (Weeks Ahead)
0.000
0.002
0.004
0.006
0
2
4
6
8
10
12
14
16
18
20
22
24
26
28
30
32
34
36
38
40
42
44
46
48
50
Johor
Kedah
Kelantan
Kuala Lumpur
Labuan
Melaka
Negeri Sembila
Pahang
Perak
Perlis
Pulua Pinang
Sabah
Sarawak
Selangor
Singapore
Terengganu

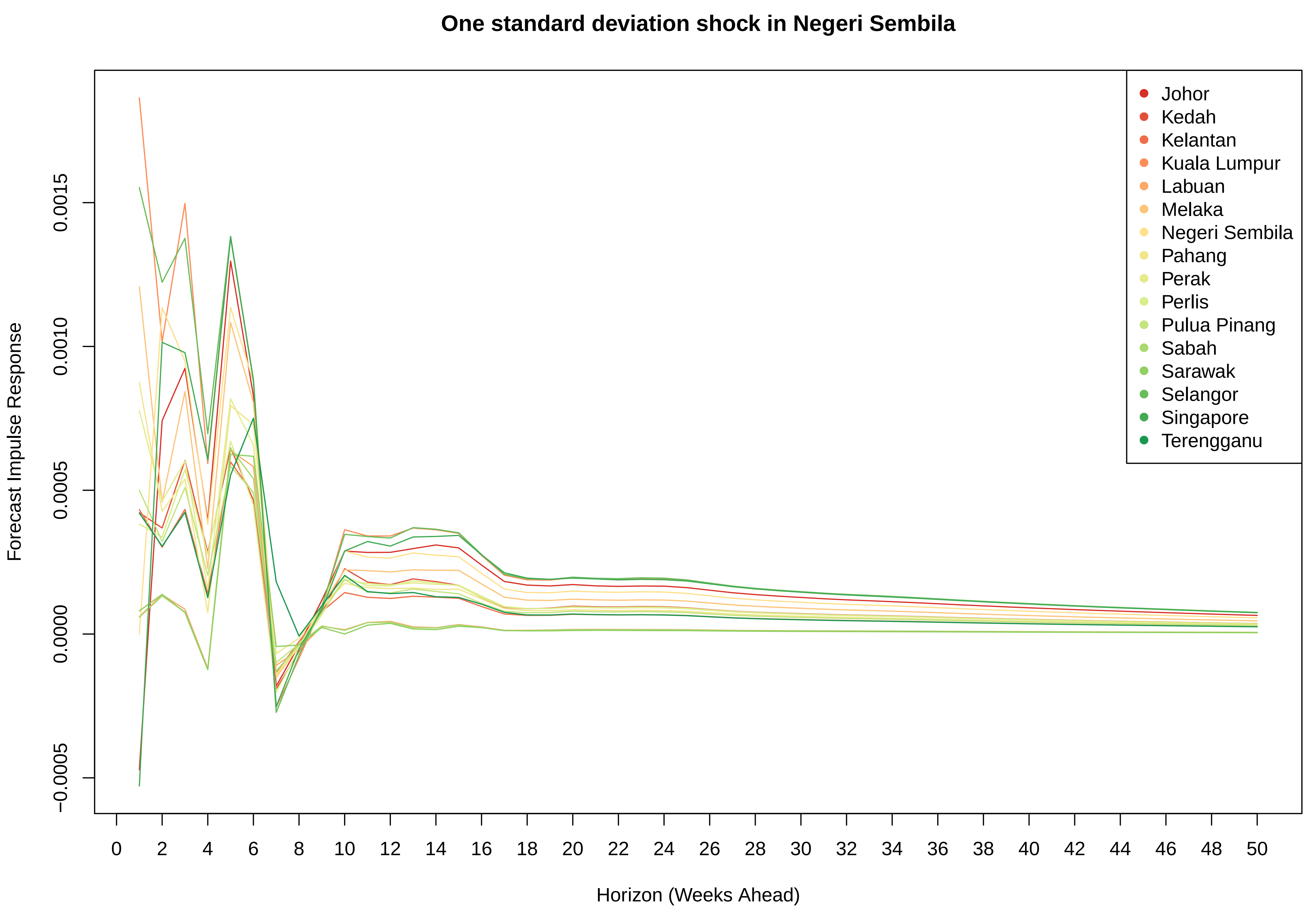
One standard deviation shock in Negeri Sembila
Forecast Impulse Response
−0.0005
0.0000
0.0005
0.0010
0.0015
0
2
4
6
8
10
12
14
16
18
20
22
24
26
28
30
32
34
36
38
40
42
44
46
48
50
Horizon (Weeks Ahead)
Johor
Kedah
Kelantan
Kuala Lumpur
Labuan
Melaka
Negeri Sembila
Pahang
Perak
Perlis
Pulua Pinang
Sabah
Sarawak
Selangor
Singapore
Terengganu

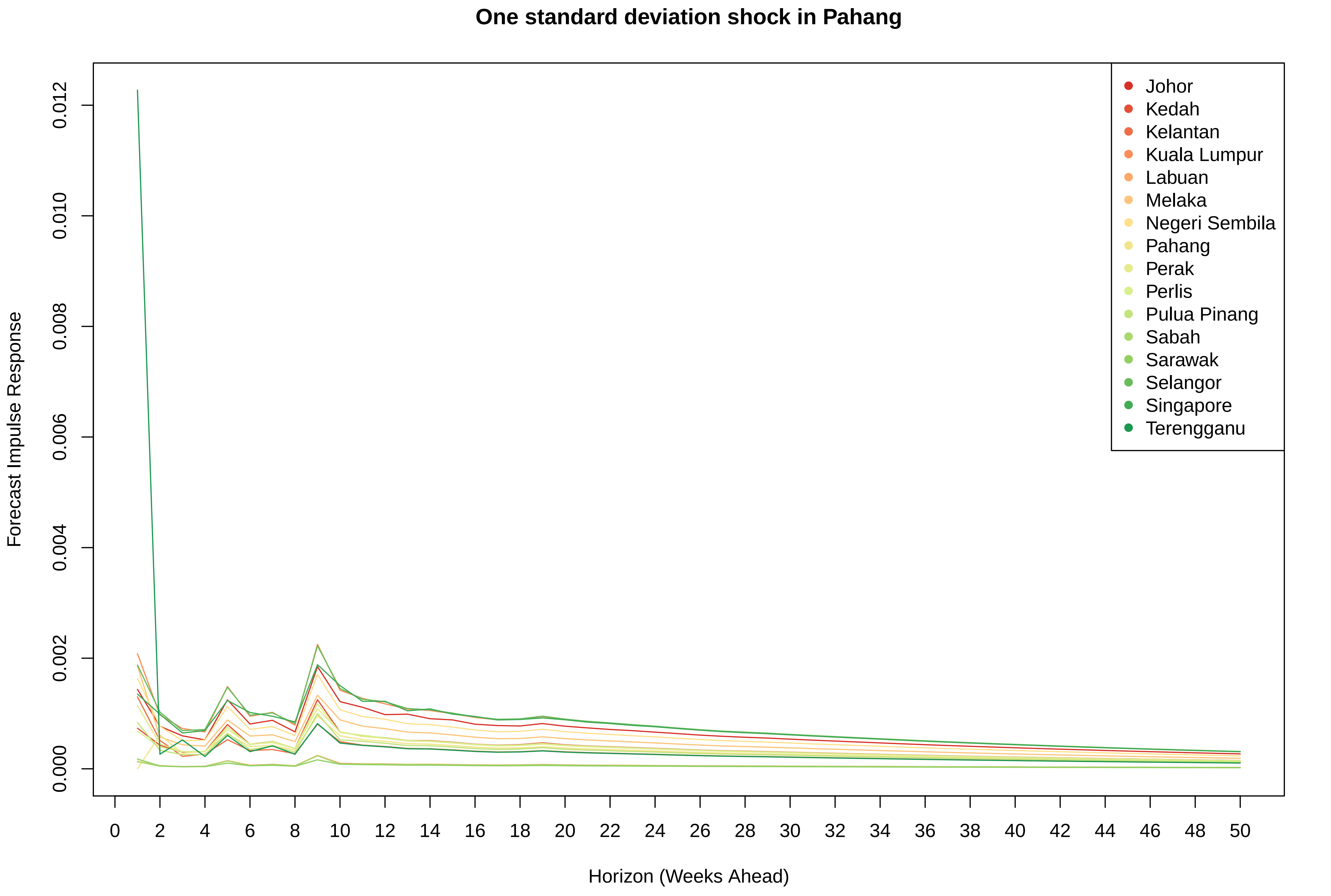

One standard deviation shock in Pahang
Forecast Impulse Response
Horizon (Weeks Ahead)
0.000
0.002
0.004
0.006
0.008
0.010
0.012
0
2
4
6
8
10
12
14
16
18
20
22
24
26
28
30
32
34
36
38
40
42
44
46
48
50
Johor
Kedah
Kelantan
Kuala Lumpur
Labuan
Melaka
Negeri Sembila
Pahang
Perak
Perlis
Pulua Pinang
Sabah
Sarawak
Selangor
Singapore
Terengganu

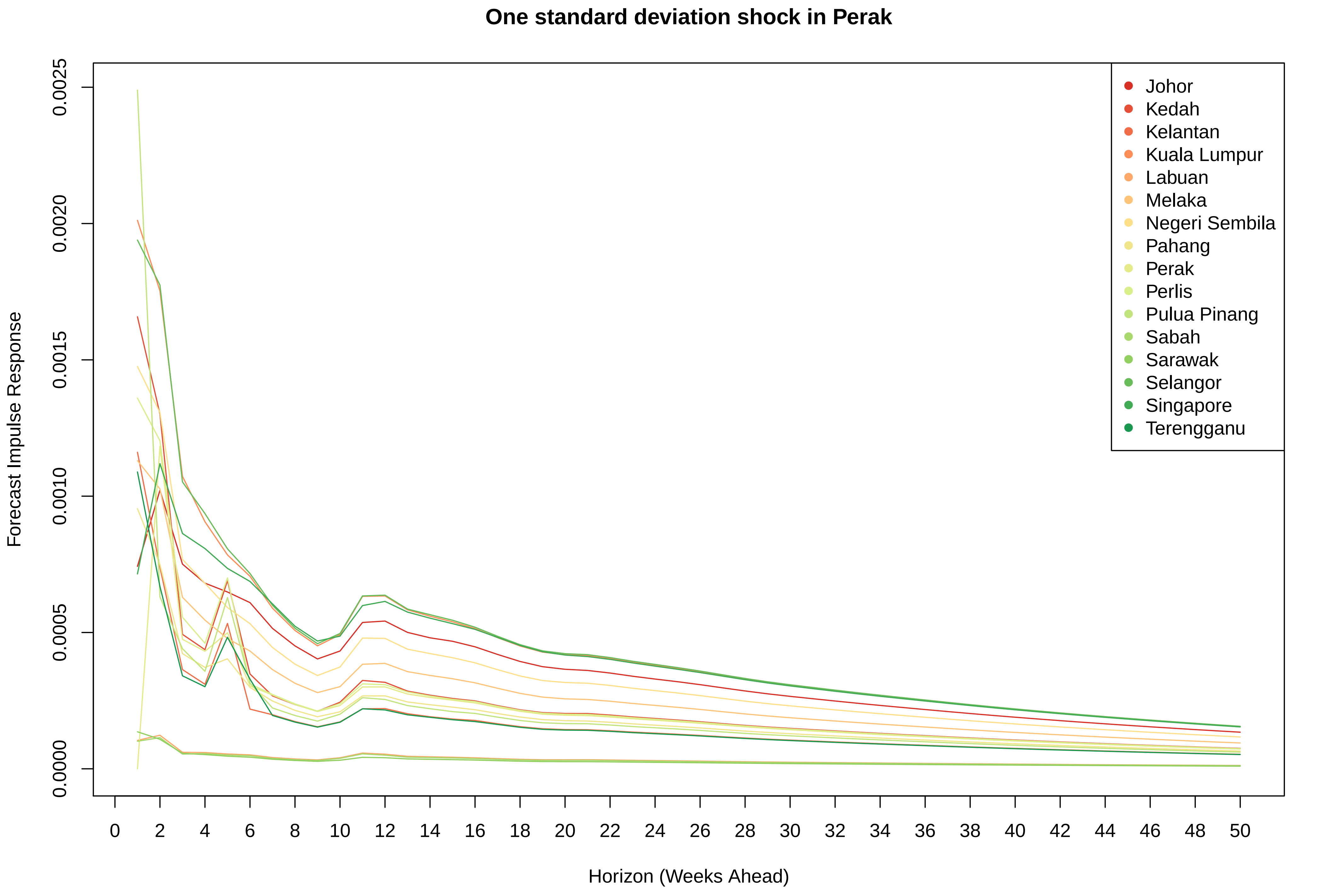

One standard deviation shock in Perak
Forecast Impulse Response
0.0000
0.0005
0.0010
0.0015
0.0020
0.0025
Horizon (Weeks Ahead)
0
2
4
6
8
10
12
14
16
18
20
22
24
26
28
30
32
34
36
38
40
42
44
46
48
50
Johor
Kedah
Kelantan
Kuala Lumpur
Labuan
Melaka
Negeri Sembila
Pahang
Perak
Perlis
Pulua Pinang
Sabah
Sarawak
Selangor
Singapore
Terengganu

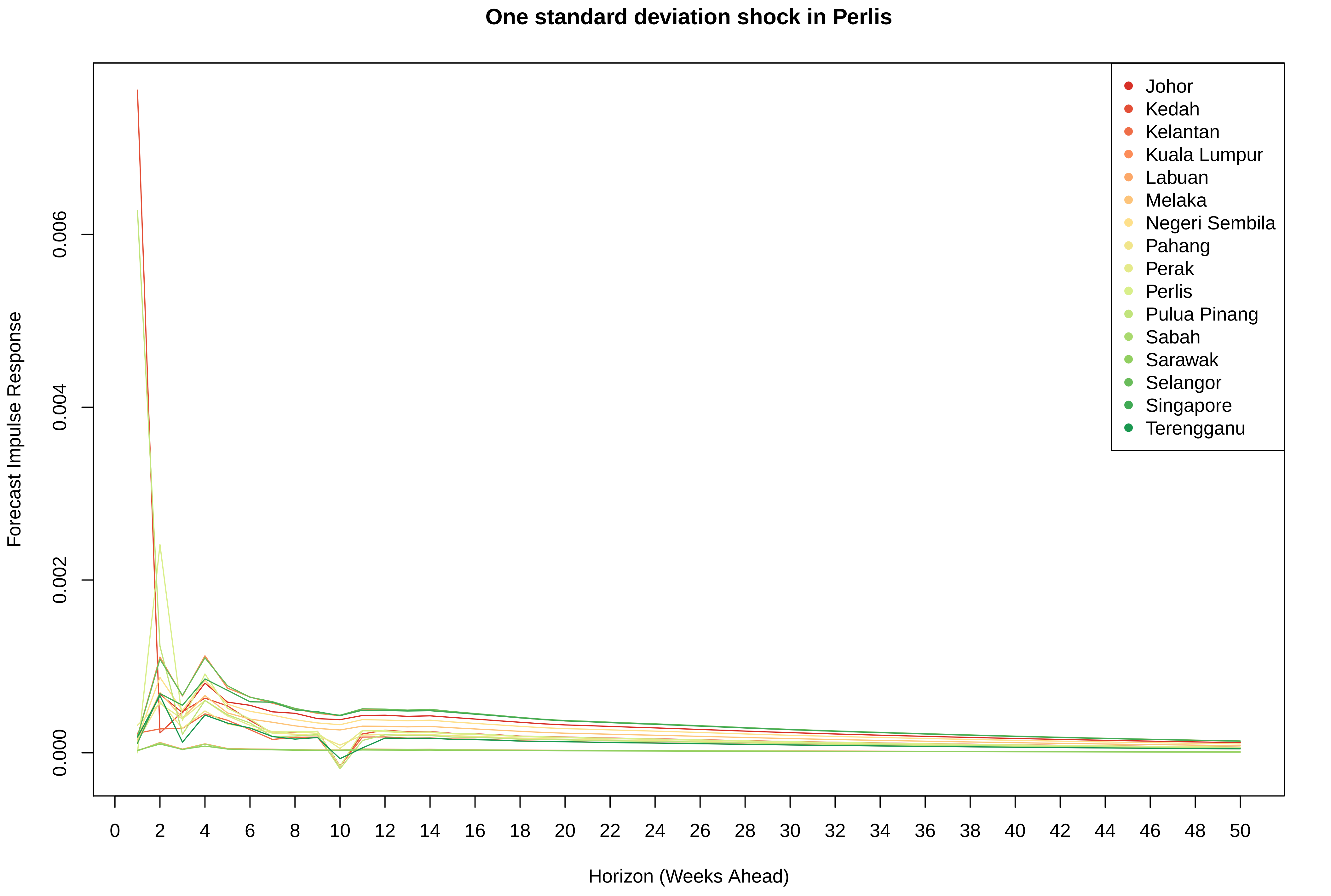
One standard deviation shock in Perlis
Forecast Impulse Response
0.000
0.002
0.004
0.006
Horizon (Weeks Ahead)
0
2
4
6
8
10
12
14
16
18
20
22
24
26
28
30
32
34
36
38
40
42
44
46
48
50
Johor
Kedah
Kelantan
Kuala Lumpur
Labuan
Melaka
Negeri Sembila
Pahang
Perak
Perlis
Pulua Pinang
Sabah
Sarawak
Selangor
Singapore
Terengganu

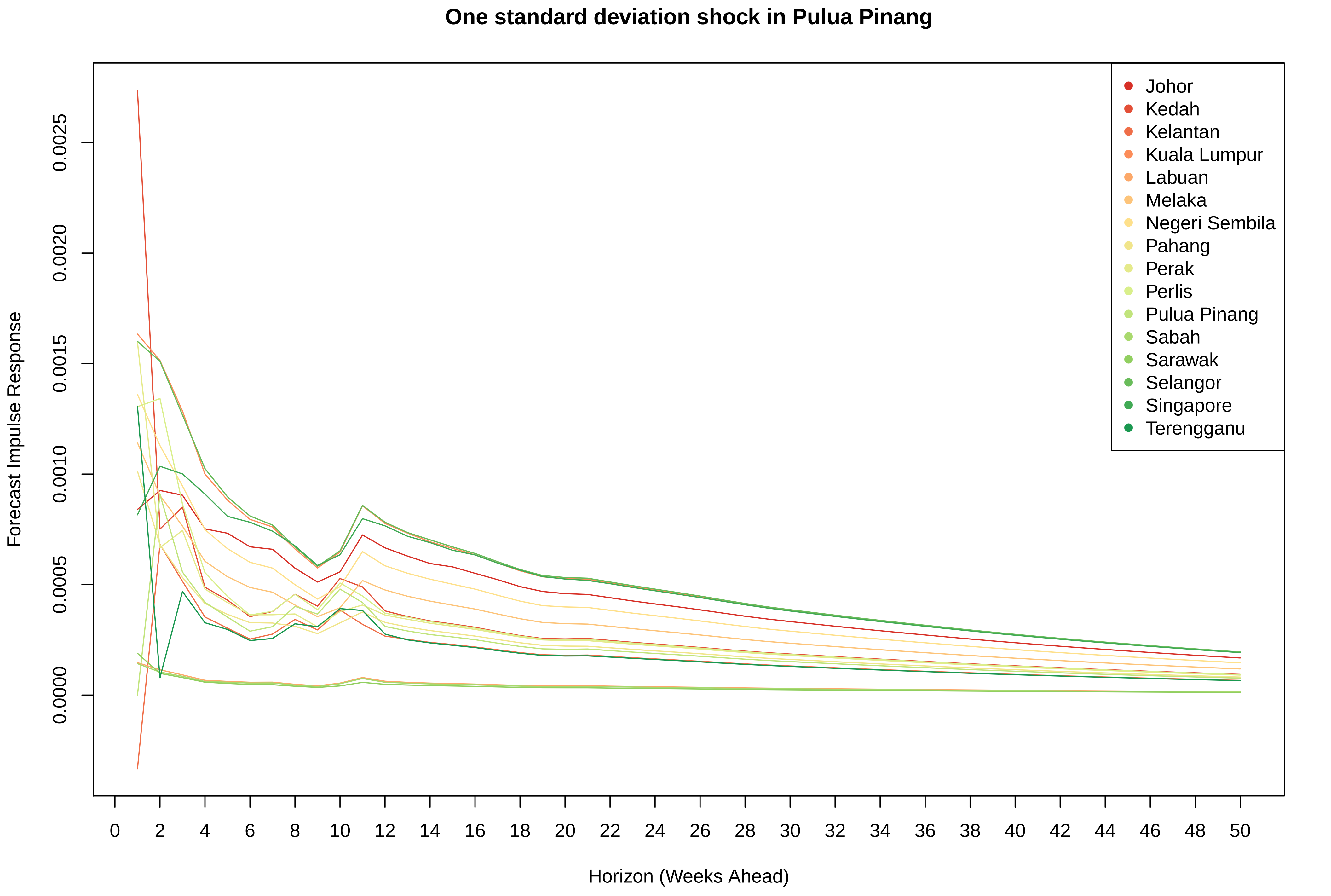
One standard deviation shock in Pulua Pinang
Forecast Impulse Response
Horizon (Weeks Ahead)
0.0000
0.0005
0.0010
0.0015
0.0020
0.0025
0
2
4
6
8
10
12
14
16
18
20
22
24
26
28
30
32
34
36
38
40
42
44
46
48
50
Johor
Kedah
Kelantan
Kuala Lumpur
Labuan
Melaka
Negeri Sembila
Pahang
Perak
Perlis
Pulua Pinang
Sabah
Sarawak
Selangor
Singapore
Terengganu

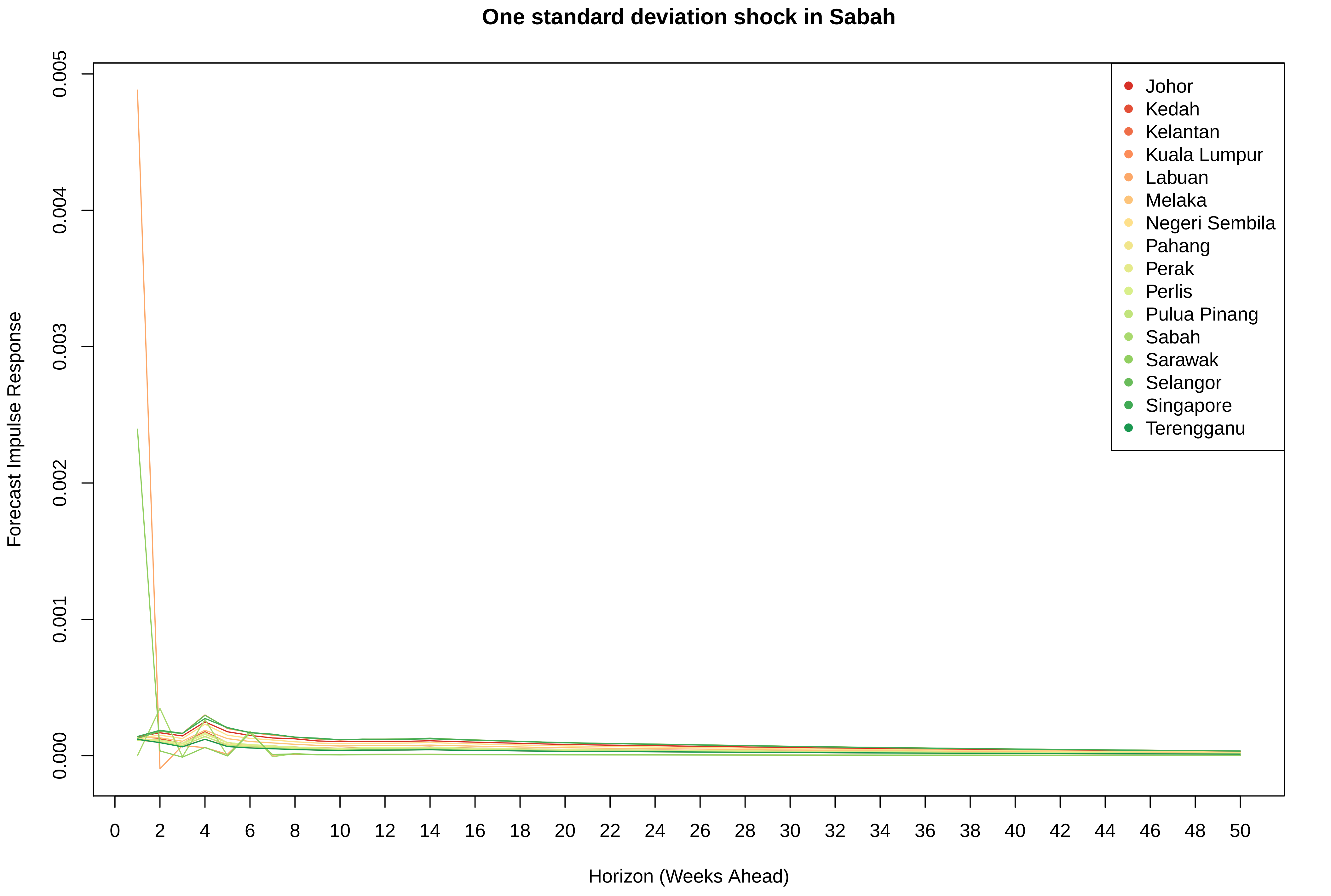

One standard deviation shock in Sabah
Forecast Impulse Response
0.000
0.001
0.002
0.003
0.004
0.005
Horizon (Weeks Ahead)
0
2
4
6
8
10
12
14
16
18
20
22
24
26
28
30
32
34
36
38
40
42
44
46
48
50
Johor
Kedah
Kelantan
Kuala Lumpur
Labuan
Melaka
Negeri Sembila
Pahang
Perak
Perlis
Pulua Pinang
Sabah
Sarawak
Selangor
Singapore
Terengganu

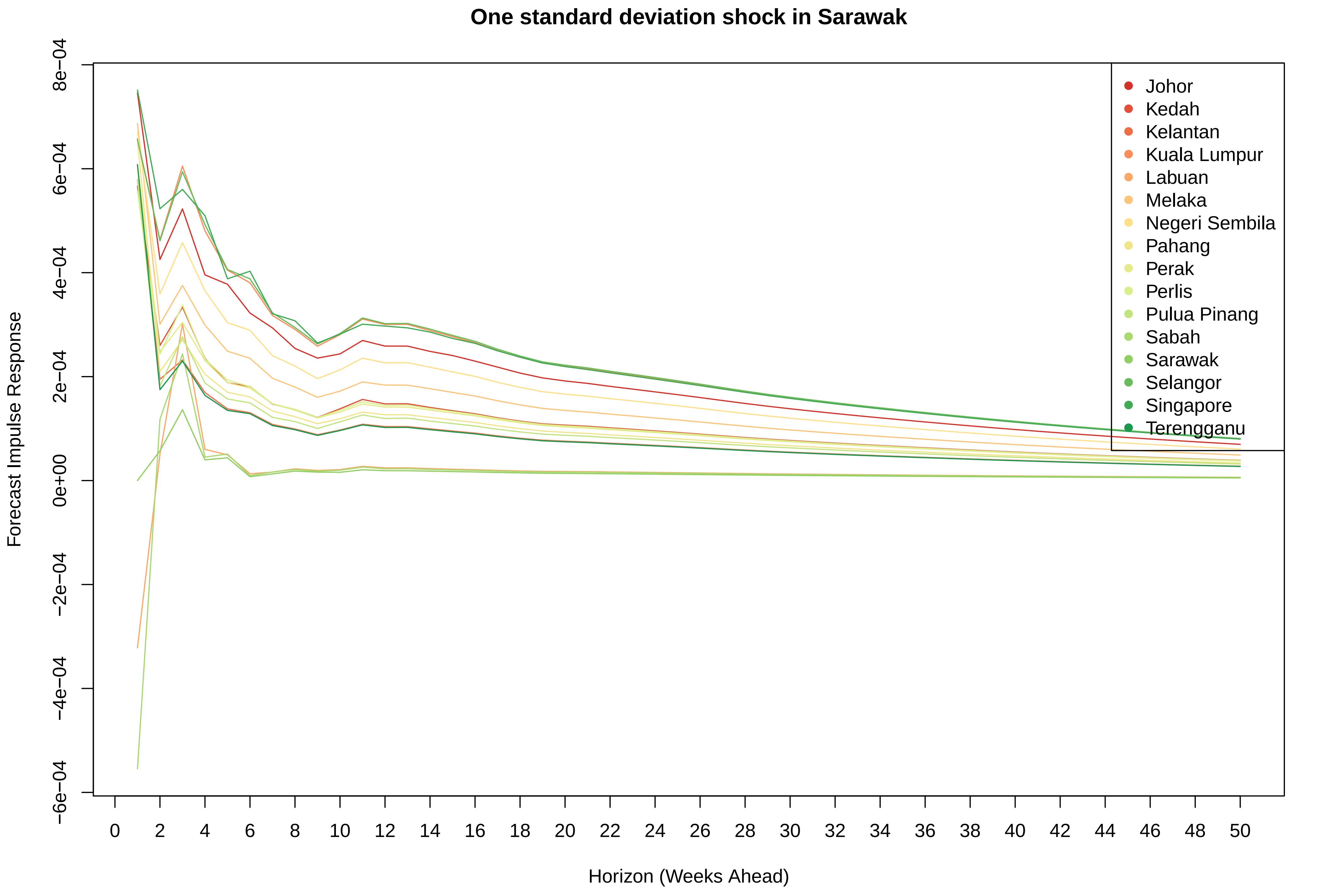

One standard deviation shock in Sarawak
Forecast Impulse Response
Horizon (Weeks Ahead)
8e−04
6e−04
4e−04
2e−04
0e+00
−2e−04
−4e−04
−6e−04
0
2
4
6
8
10
12
14
16
18
20
22
24
26
28
30
32
34
36
38
40
42
44
46
48
50
Johor
Kedah
Kelantan
Kuala Lumpur
Labuan
Melaka
Negeri Sembila
Pahang
Perak
Perlis
Pulua Pinang
Sabah
Sarawak
Selangor
Singapore
Terengganu

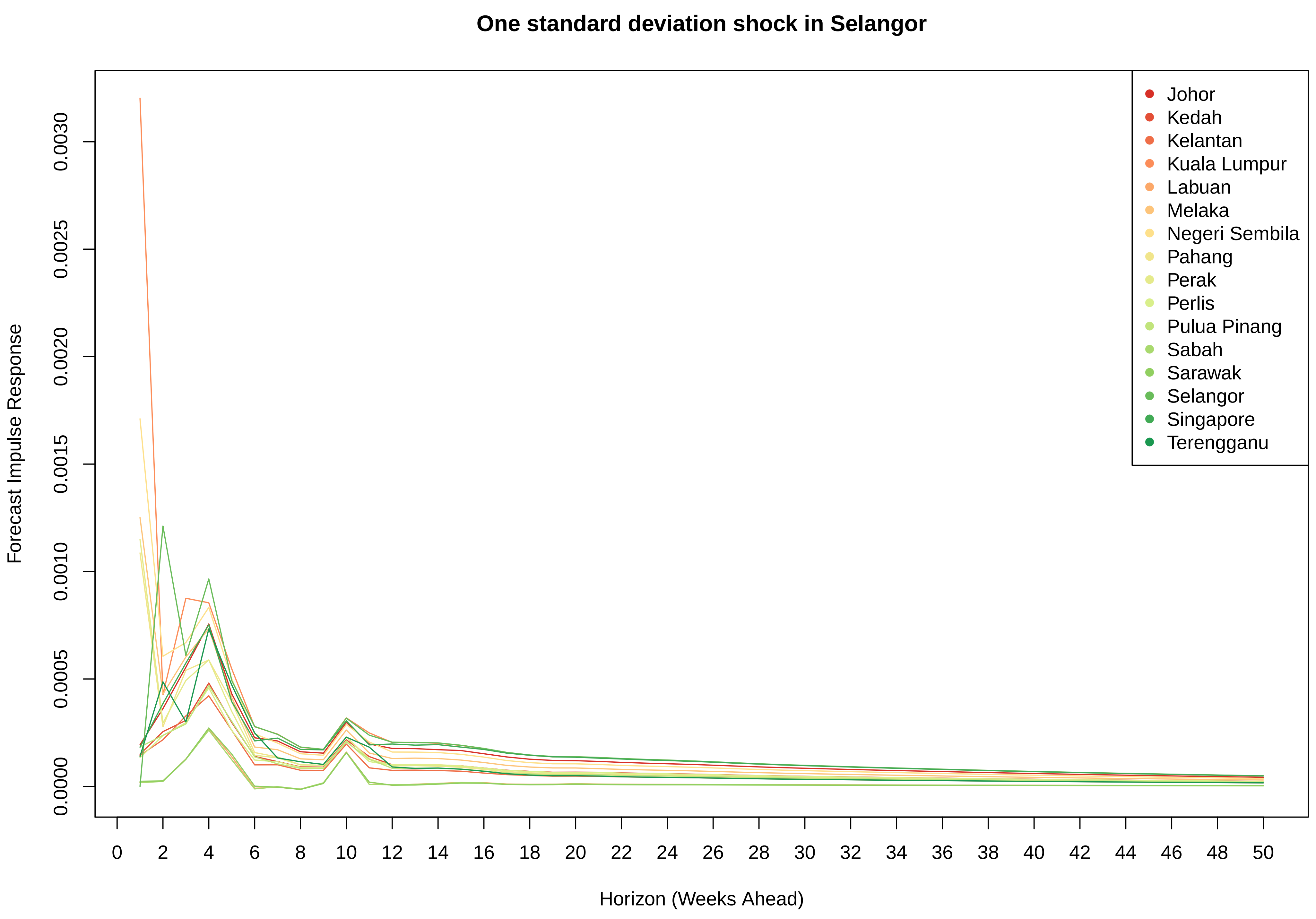
One standard deviation shock in Selangor
Forecast Impulse Response
Horizon (Weeks Ahead)
0.0000
0.0005
0.0010
0.0015
0.0020
0.0025
0.0030
0 2 4 6 8 10 12 14 16 18 20 22 24 26 28 30 32 34 36 38 40 42 44 46 48 50
Johor
Kedah
Kelantan
Kuala Lumpur
Labuan
Melaka
Negeri Sembila
Pahang
Perak
Perlis
Pulua Pinang
Sabah
Sarawak
Selangor
Singapore
Terengganu

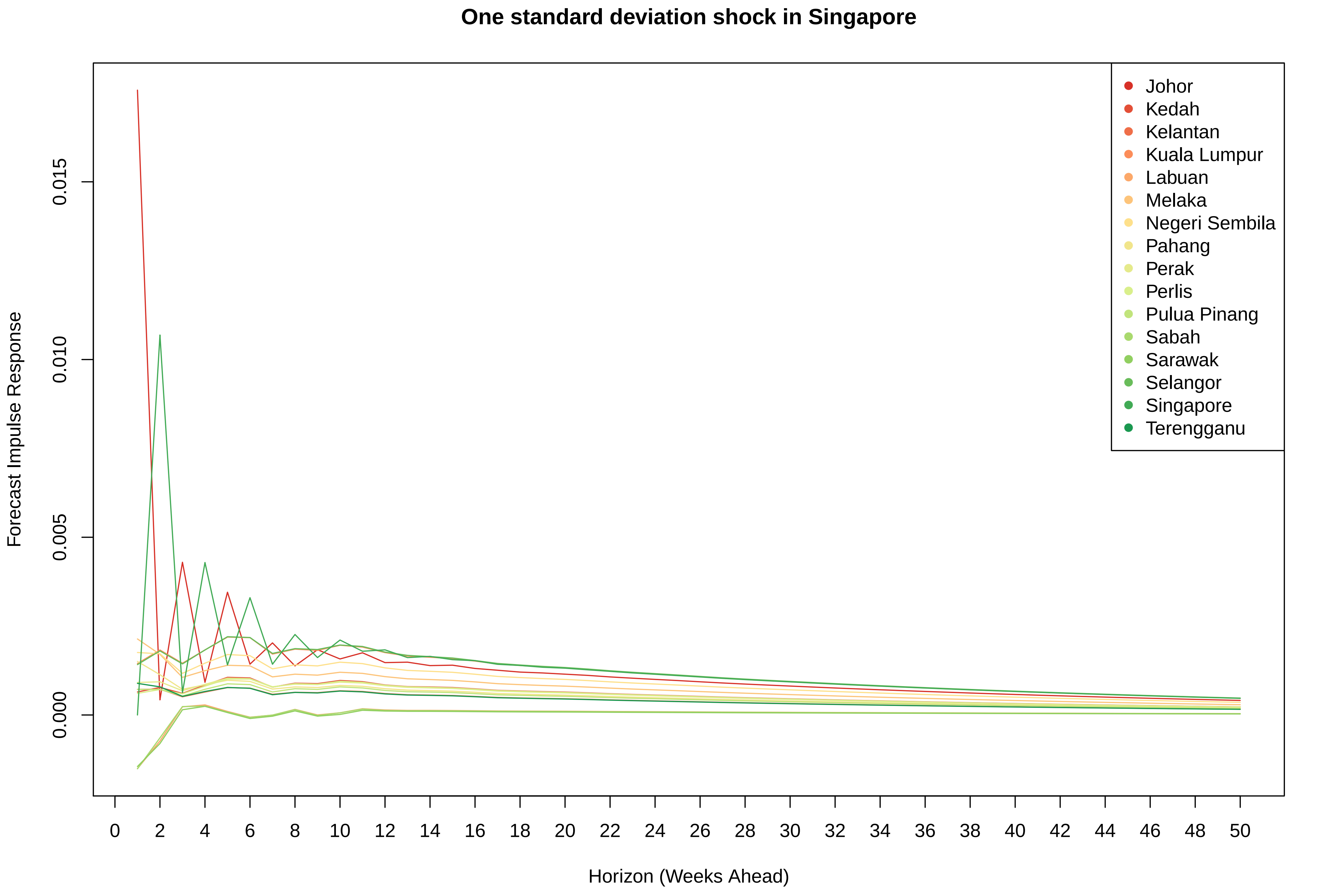
One standard deviation shock in Singapore
Forecast Impulse Response
Horizon (Weeks Ahead)
0.000
0.005
0.010
0.015
0
2
4
6
8
10
12
14
16
18
20
22
24
26
28
30
32
34
36
38
40
42
44
46
48
50
Johor
Kedah
Kelantan
Kuala Lumpur
Labuan
Melaka
Negeri Sembila
Pahang
Perak
Perlis
Pulua Pinang
Sabah
Sarawak
Selangor
Singapore
Terengganu

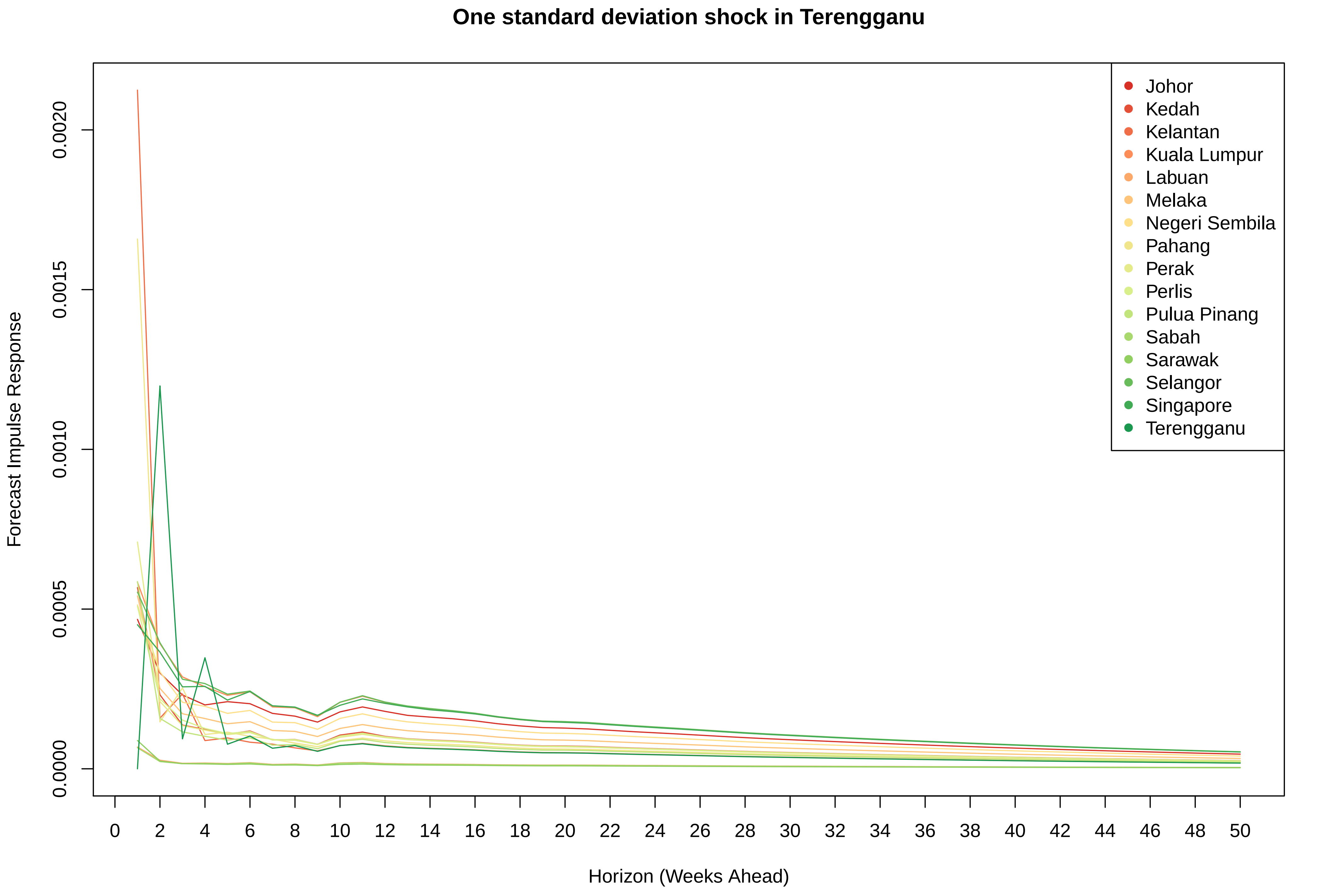
One standard deviation shock in Terengganu
Forecast Impulse Response
0.0000
0.0005
0.0010
0.0015
0.0020
Horizon (Weeks Ahead)
0 2 4 6 8 10 12 14 16 18 20 22 24 26 28 30 32 34 36 38 40 42 44 46 48 50
Johor
Kedah
Kelantan
Kuala Lumpur
Labuan
Melaka
Negeri Sembila
Pahang
Perak
Perlis
Pulua Pinang
Sabah
Sarawak
Selangor
Singapore
Terengganu

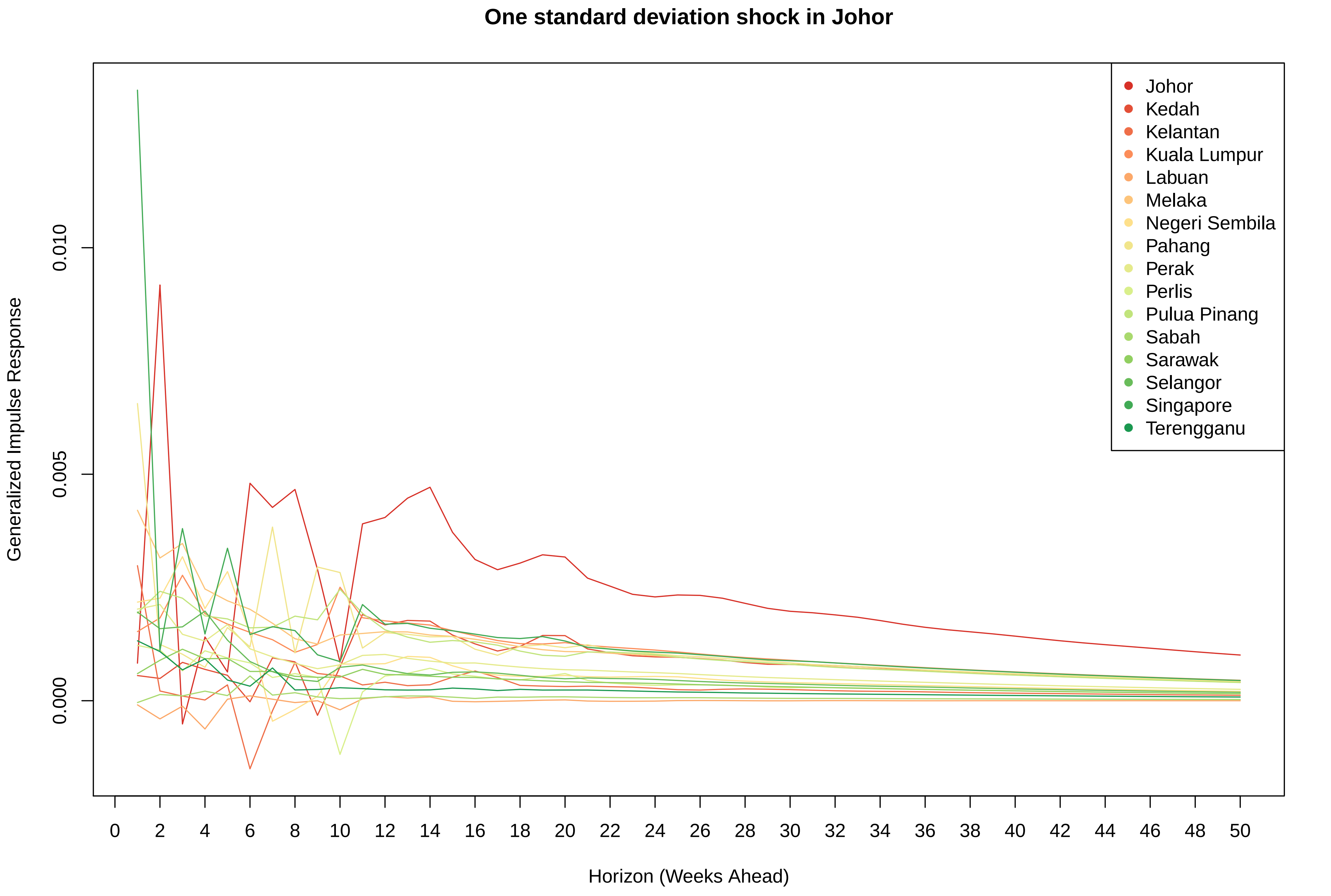

One standard deviation shock in Johor
Generalized Impulse Response
Horizon (Weeks Ahead)
0.000
0.005
0.010
0
2
4
6
8
10
12
14
16
18
20
22
24
26
28
30
32
34
36
38
40
42
44
46
48
50
Johor
Kedah
Kelantan
Kuala Lumpur
Labuan
Melaka
Negeri Sembila
Pahang
Perak
Perlis
Pulua Pinang
Sabah
Sarawak
Selangor
Singapore
Terengganu

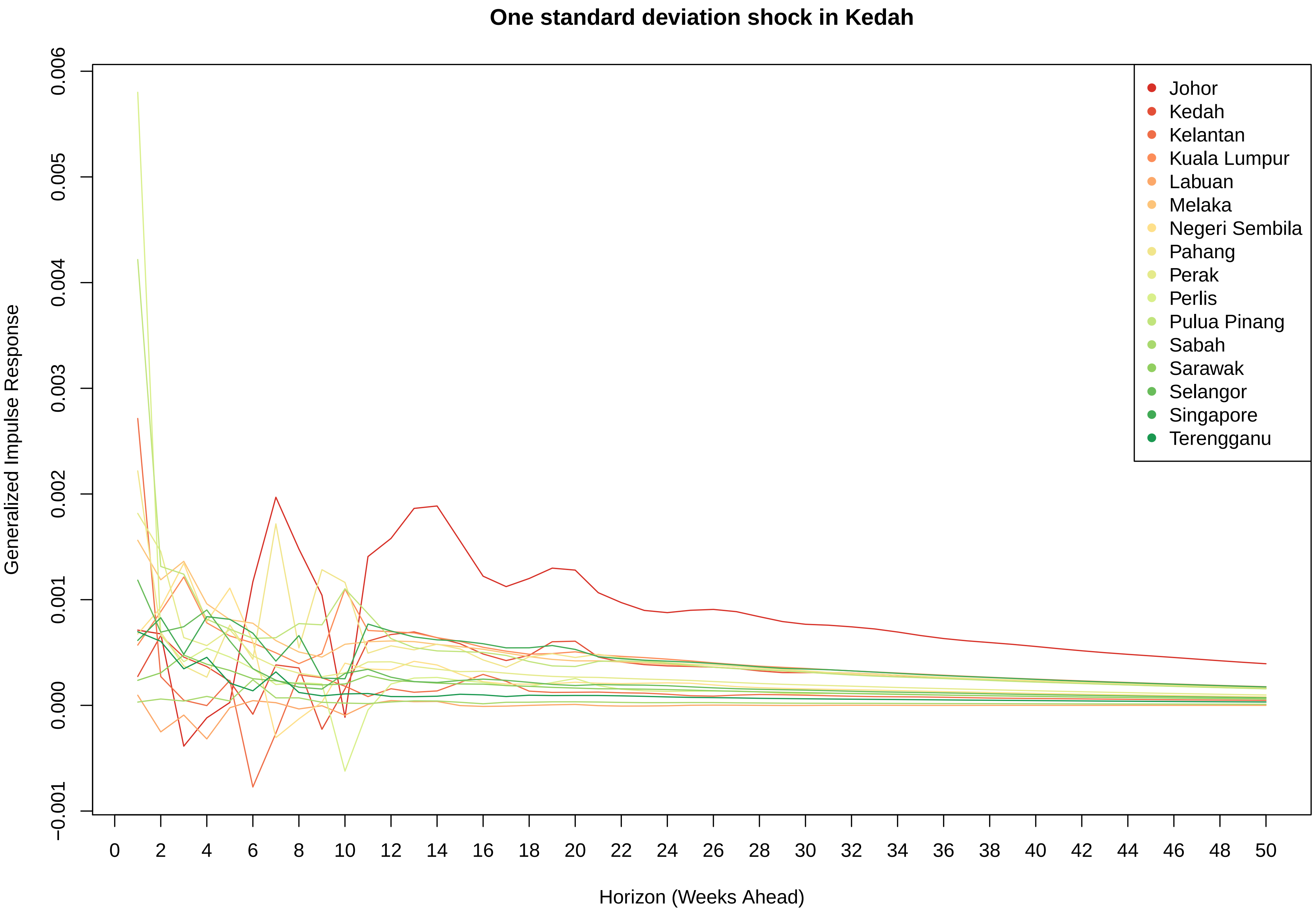

One standard deviation shock in Kedah
Generalized Impulse Response
Horizon (Weeks Ahead)
Johor
Kedah
Kelantan
Kuala Lumpur
Labuan
Melaka
Negeri Sembila
Pahang
Perak
Perlis
Pulua Pinang
Sabah
Sarawak
Selangor
Singapore
Terengganu

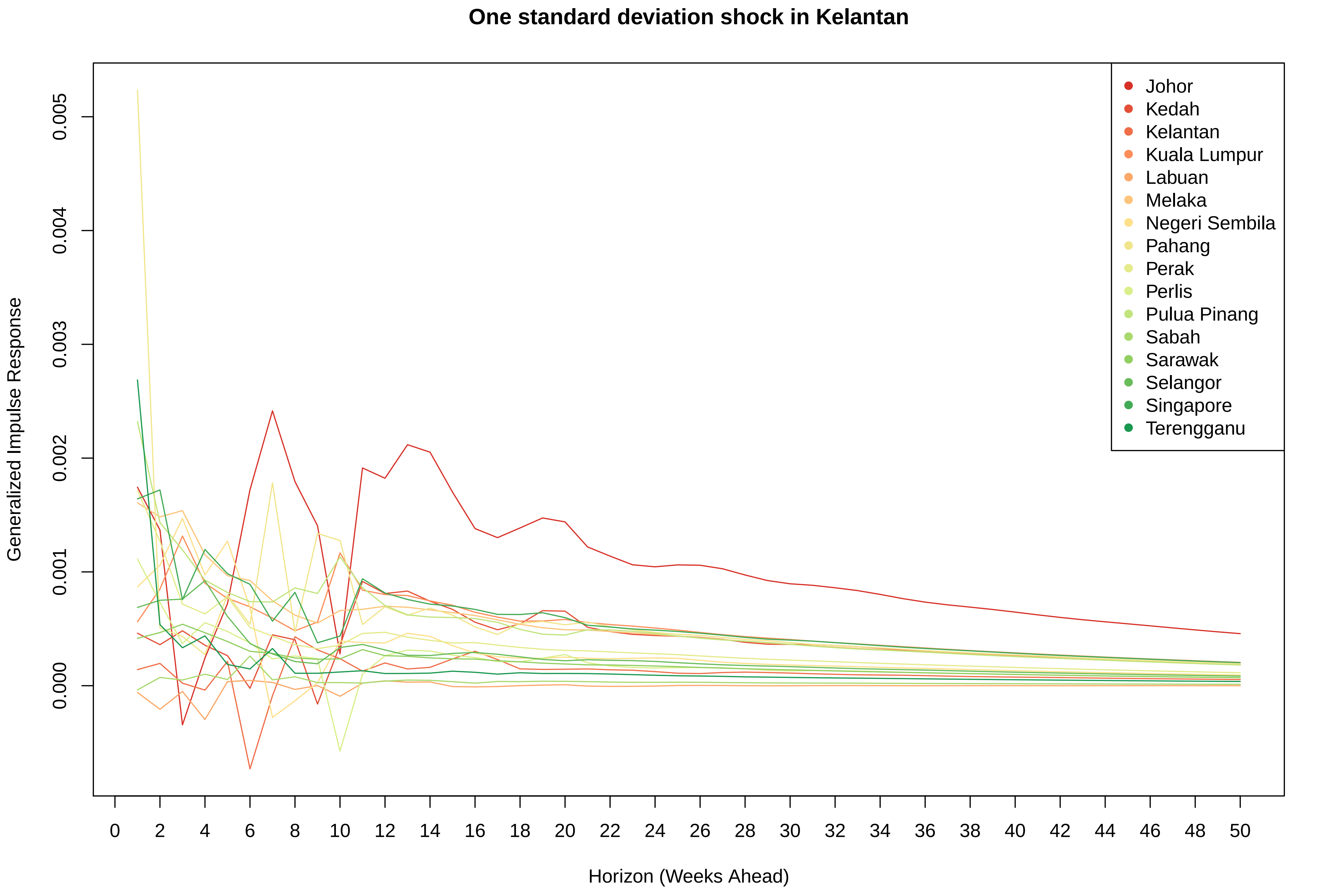

One standard deviation shock in Kelantan
Generalized Impulse Response
0.000
0.001
0.002
0.003
0.004
0.005
0
2
4
6
8
10
12
14
16
18
20
22
24
26
28
30
32
34
36
38
40
42
44
46
48
50
Horizon (Weeks Ahead)
Johor
Kedah
Kelantan
Kuala Lumpur
Labuan
Melaka
Negeri Sembila
Pahang
Perak
Perlis
Pulua Pinang
Sabah
Sarawak
Selangor
Singapore
Terengganu

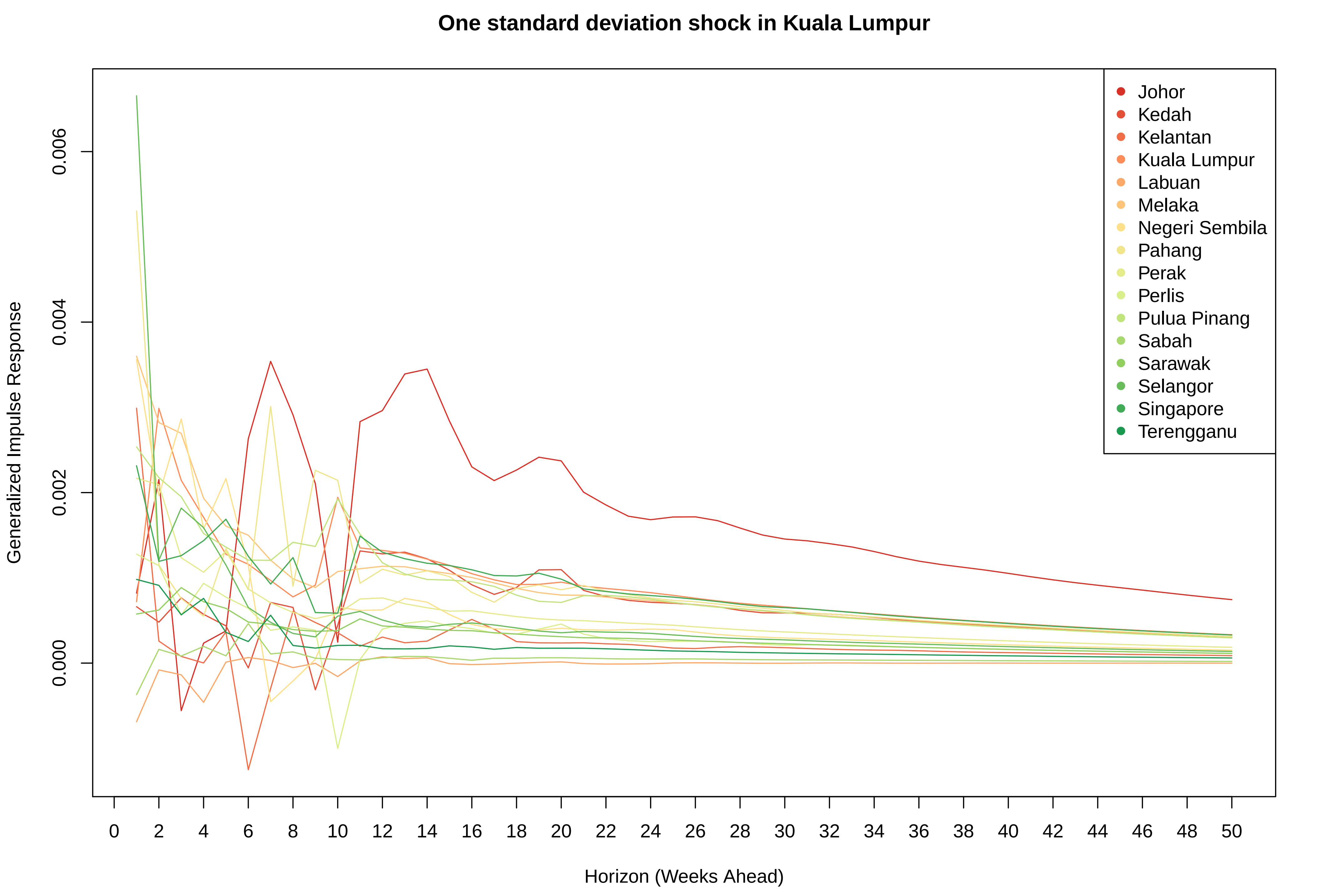
One standard deviation shock in Kuala Lumpur
Generalized Impulse Response
0.000
0.002
0.004
0.006
Horizon (Weeks Ahead)
0
2
4
6
8
10
12
14
16
18
20
22
24
26
28
30
32
34
36
38
40
42
44
46
48
50
Johor
Kedah
Kelantan
Kuala Lumpur
Labuan
Melaka
Negeri Sembila
Pahang
Perak
Perlis
Pulua Pinang
Sabah
Sarawak
Selangor
Singapore
Terengganu

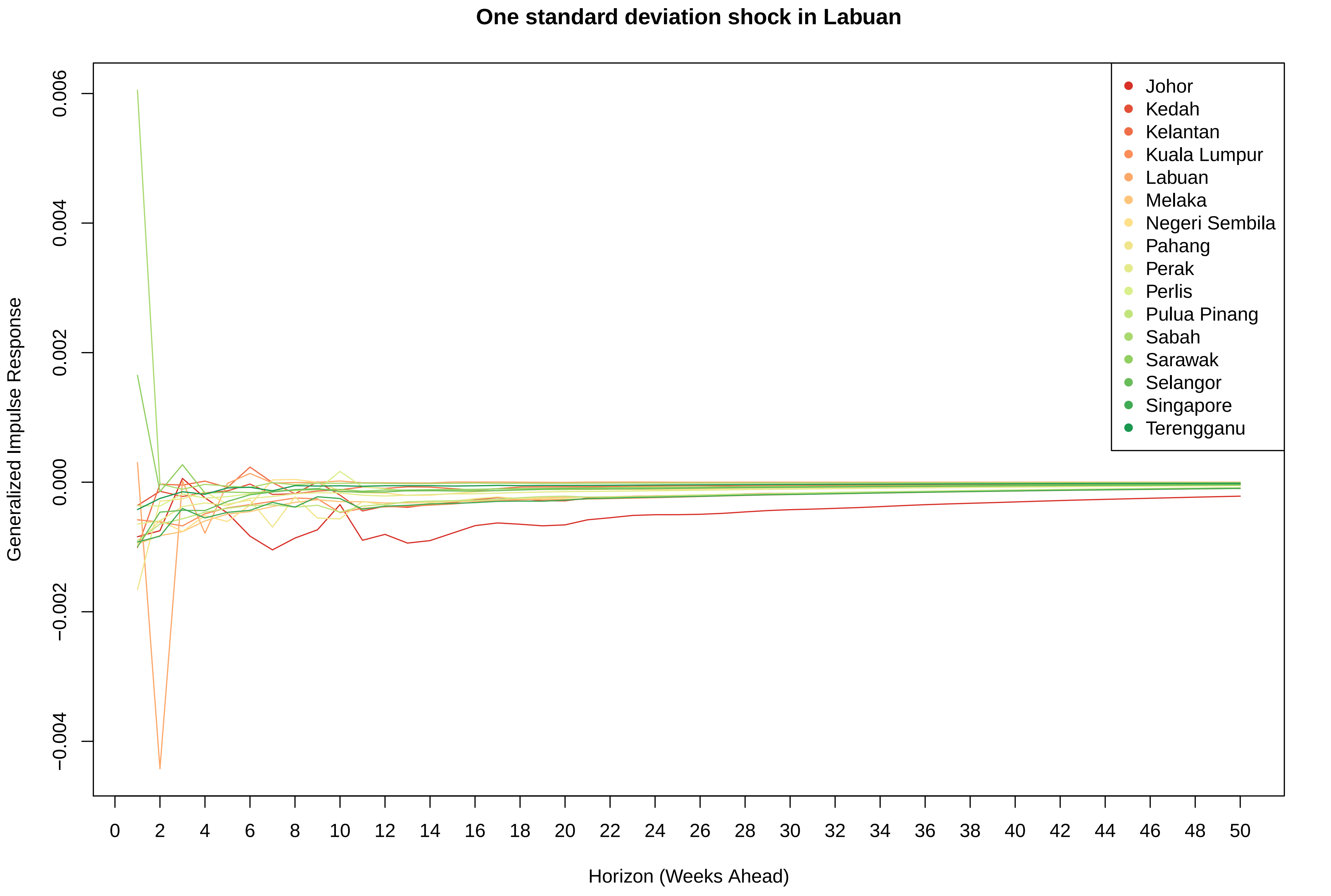
One standard deviation shock in Labuan
Generalized Impulse Response
Horizon (Weeks Ahead)
0.006
0.004
0.002
0.000
−0.002
−0.004
0
2
4
6
8
10
12
14
16
18
20
22
24
26
28
30
32
34
36
38
40
42
44
46
48
50
Johor
Kedah
Kelantan
Kuala Lumpur
Labuan
Melaka
Negeri Sembila
Pahang
Perak
Perlis
Pulua Pinang
Sabah
Sarawak
Selangor
Singapore
Terengganu

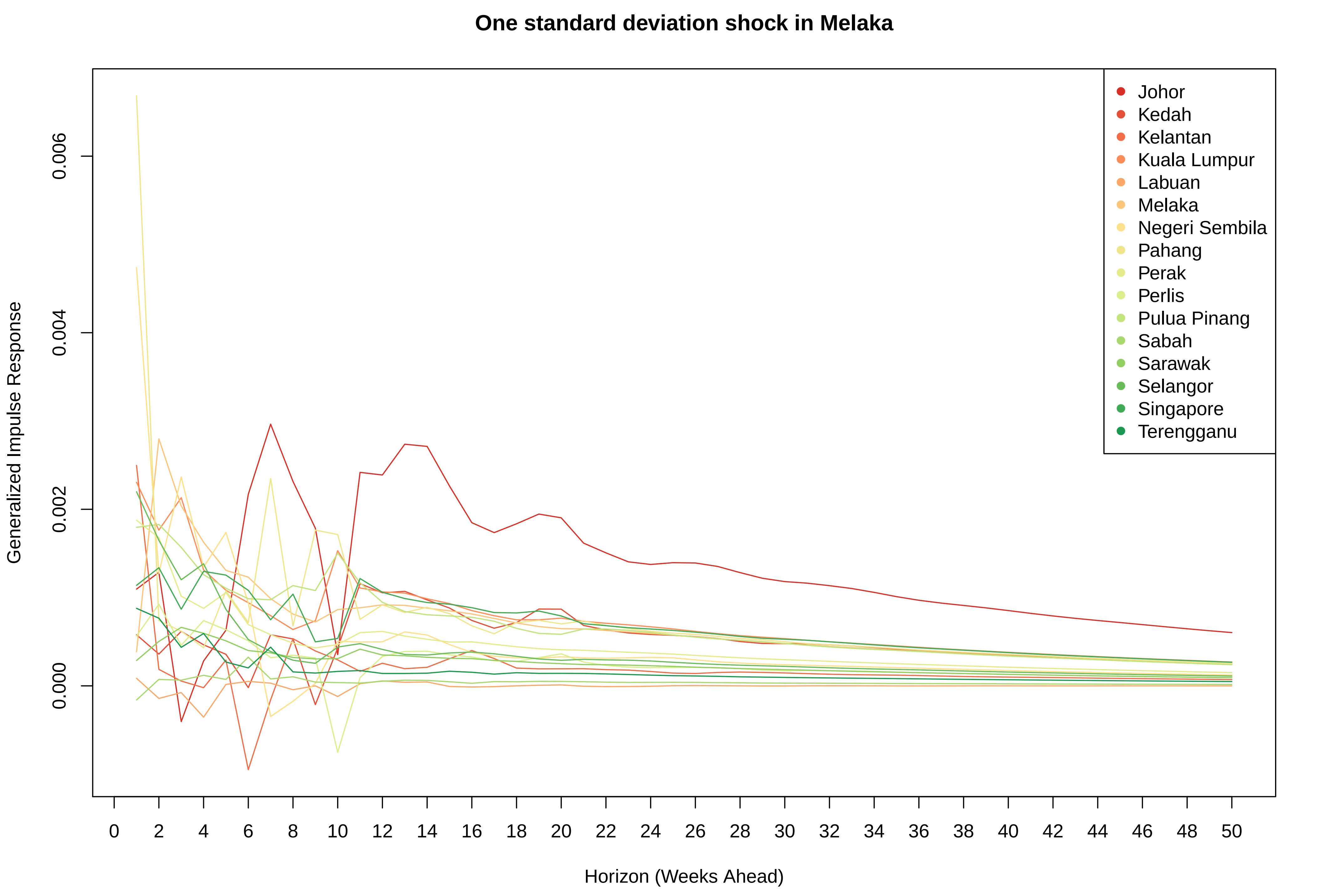

One standard deviation shock in Melaka
Generalized Impulse Response
0.000
0.002
0.004
0.006
Horizon (Weeks Ahead)
0
2
4
6
8
10
12
14
16
18
20
22
24
26
28
30
32
34
36
38
40
42
44
46
48
50
Johor
Kedah
Kelantan
Kuala Lumpur
Labuan
Melaka
Negeri Sembila
Pahang
Perak
Perlis
Pulua Pinang
Sabah
Sarawak
Selangor
Singapore
Terengganu

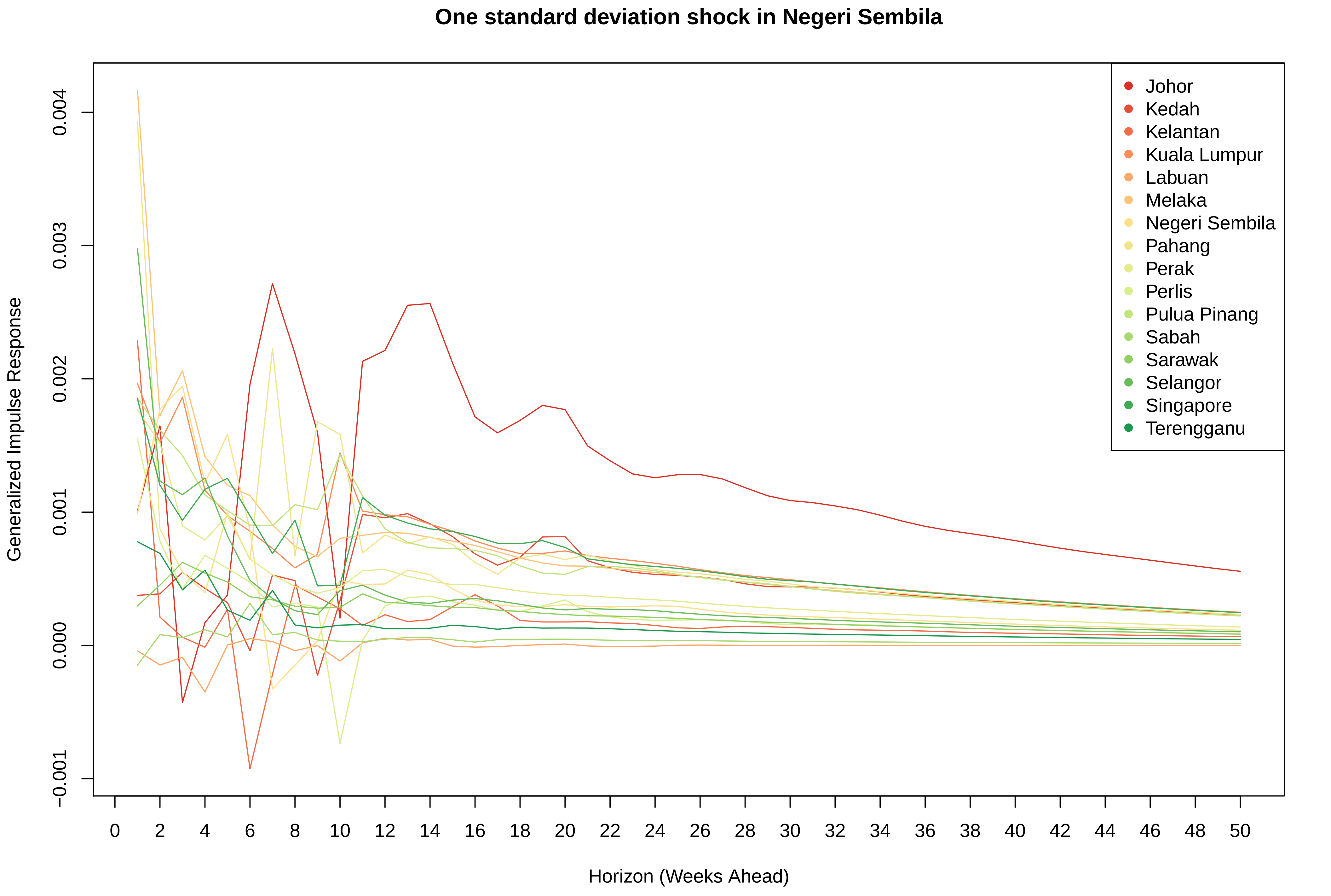
One standard deviation shock in Negeri Sembila
Generalized Impulse Response
Horizon (Weeks Ahead)
−0.001
0.000
0.001
0.002
0.003
0.004
0
2
4
6
8
10
12
14
16
18
20
22
24
26
28
30
32
34
36
38
40
42
44
46
48
50
Johor
Kedah
Kelantan
Kuala Lumpur
Labuan
Melaka
Negeri Sembila
Pahang
Perak
Perlis
Pulua Pinang
Sabah
Sarawak
Selangor
Singapore
Terengganu

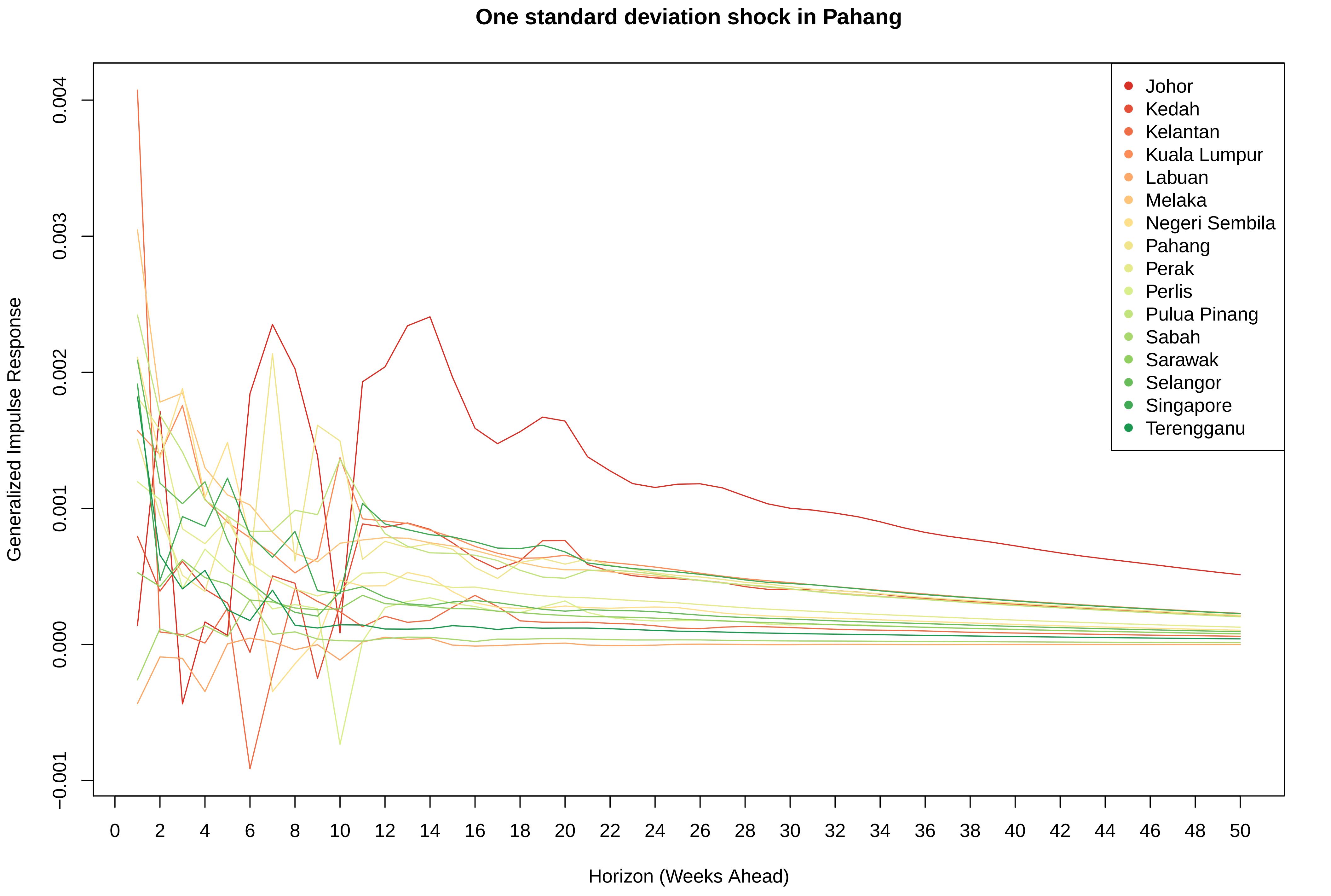
One standard deviation shock in Pahang
Generalized Impulse Response
Horizon (Weeks Ahead)
0.004
0.003
0.002
0.001
0.000
−0.001
0 2 4 6 8 10 12 14 16 18 20 22 24 26 28 30 32 34 36 38 40 42 44 46 48 50
Johor
Kedah
Kelantan
Kuala Lumpur
Labuan
Melaka
Negeri Sembila
Pahang
Perak
Perlis
Pulua Pinang
Sabah
Sarawak
Selangor
Singapore
Terengganu

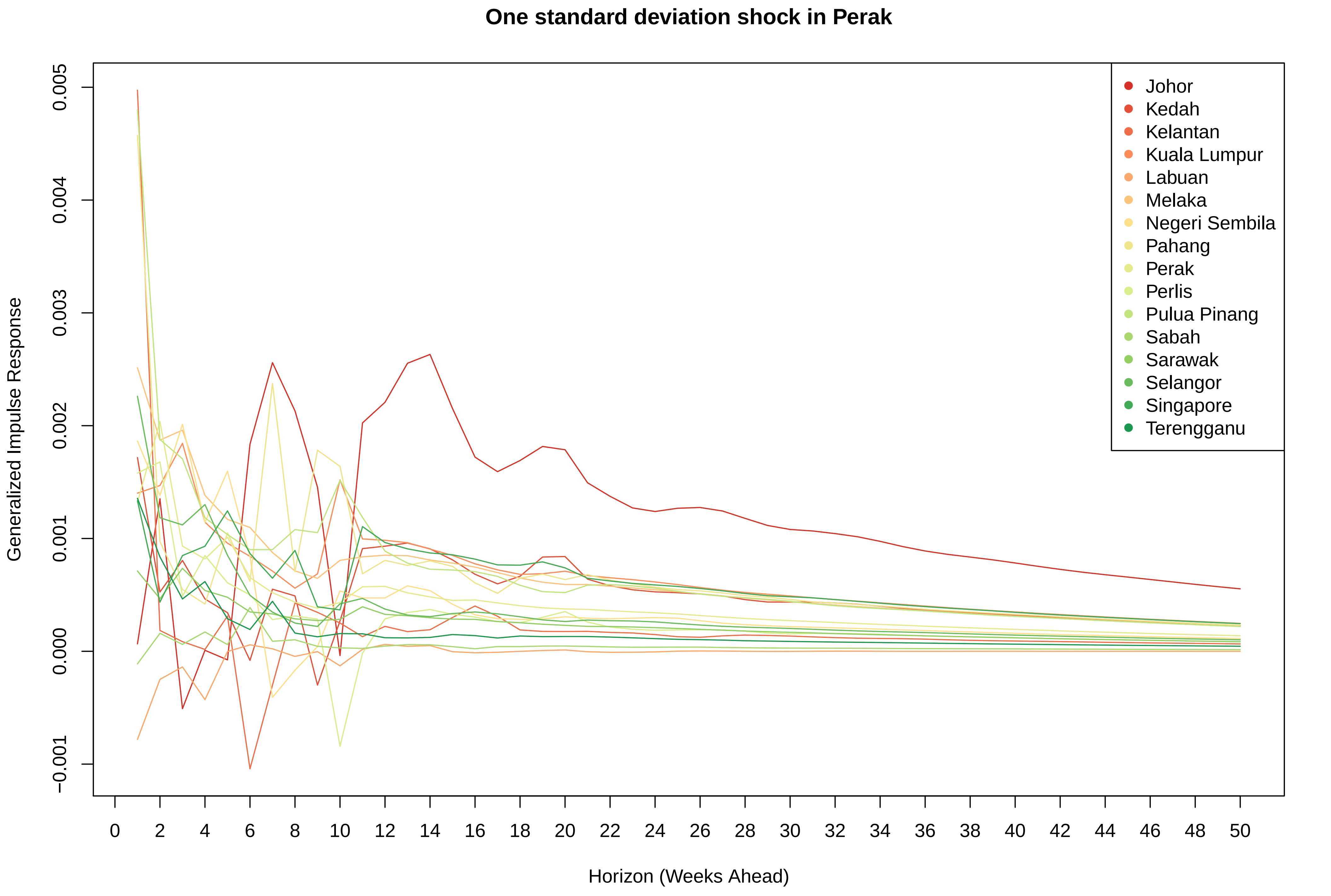

One standard deviation shock in Perak
Generalized Impulse Response
Horizon (Weeks Ahead)
-0.001
0.000
0.001
0.002
0.003
0.004
0.005
0
2
4
6
8
10
12
14
16
18
20
22
24
26
28
30
32
34
36
38
40
42
44
46
48
50
Johor
Kedah
Kelantan
Kuala Lumpur
Labuan
Melaka
Negeri Sembila
Pahang
Perak
Perlis
Pulua Pinang
Sabah
Sarawak
Selangor
Singapore
Terengganu

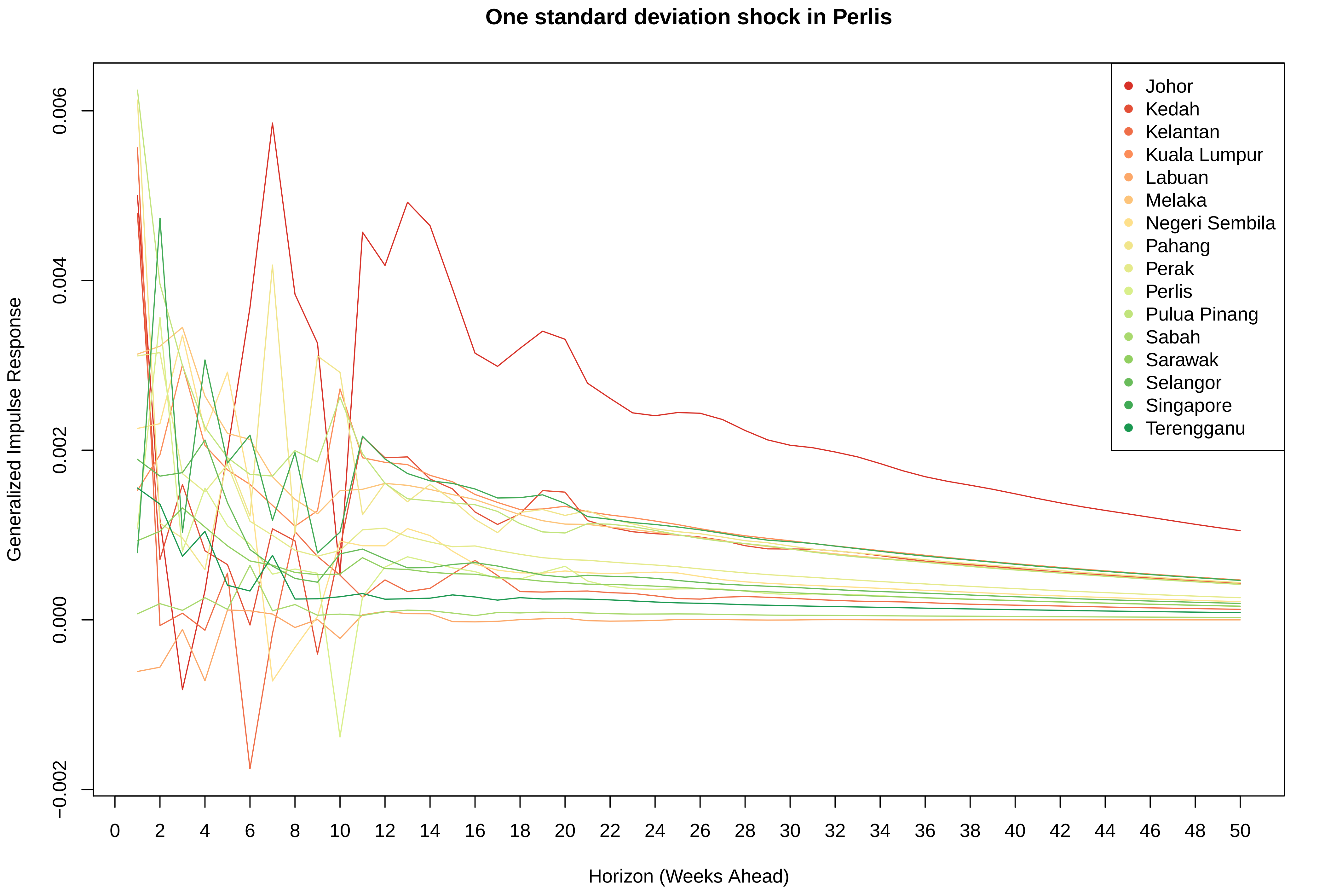

One standard deviation shock in Perlis
Generalized Impulse Response
Horizon (Weeks Ahead)
−0.002
0.000
0.002
0.004
0.006
0
2
4
6
8
10
12
14
16
18
20
22
24
26
28
30
32
34
36
38
40
42
44
46
48
50
Johor
Kedah
Kelantan
Kuala Lumpur
Labuan
Melaka
Negeri Sembila
Pahang
Perak
Perlis
Pulua Pinang
Sabah
Sarawak
Selangor
Singapore
Terengganu

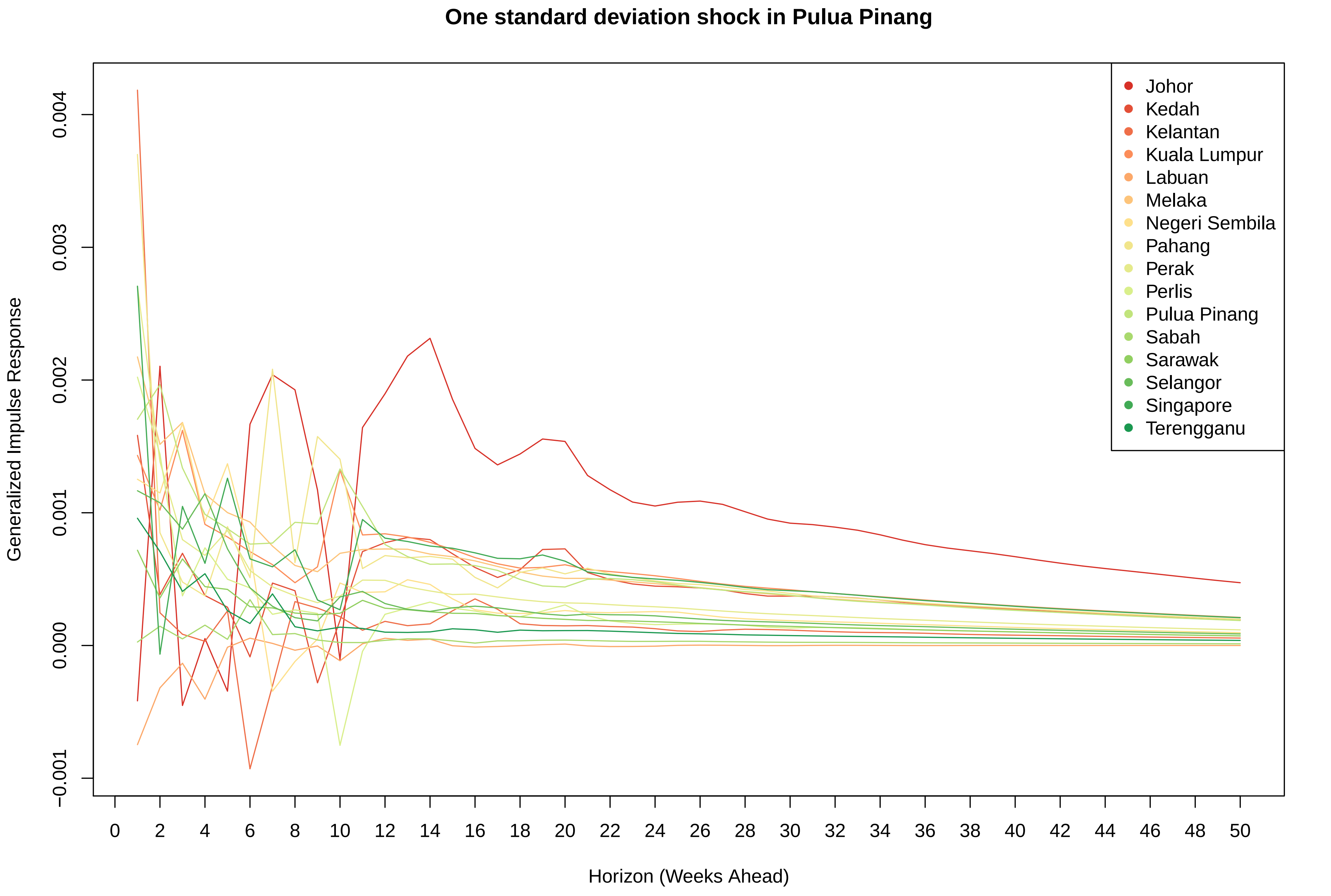
One standard deviation shock in Pulua Pinang
Generalized Impulse Response
Horizon (Weeks Ahead)
Johor
Kedah
Kelantan
Kuala Lumpur
Labuan
Melaka
Negeri Sembila
Pahang
Perak
Perlis
Pulua Pinang
Sabah
Sarawak
Selangor
Singapore
Terengganu

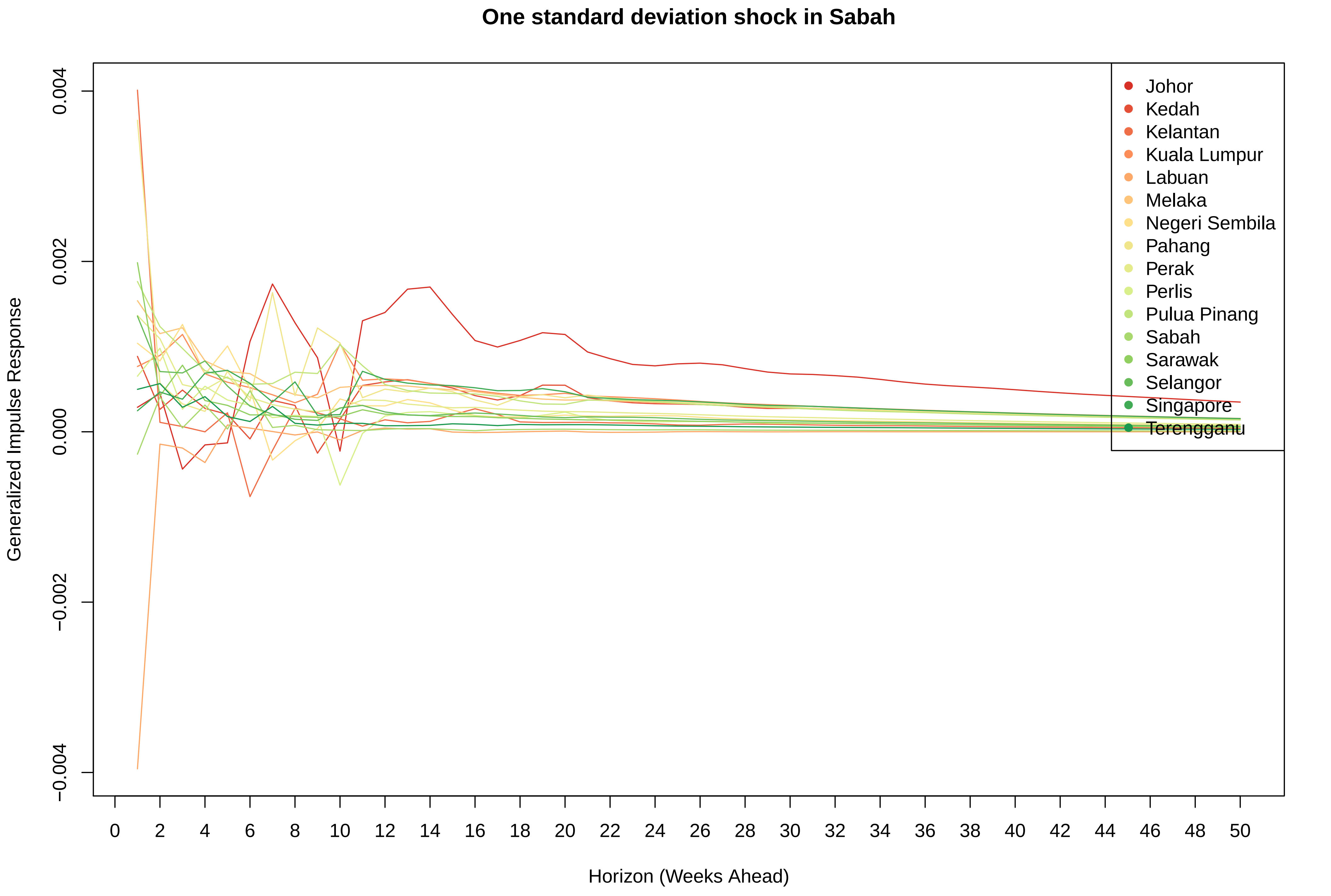
One standard deviation shock in Sabah
Generalized Impulse Response
0.004
0.002
0.000
−0.002
−0.004
Horizon (Weeks Ahead)
0
2
4
6
8
10
12
14
16
18
20
22
24
26
28
30
32
34
36
38
40
42
44
46
48
50
Johor
Kedah
Kelantan
Kuala Lumpur
Labuan
Melaka
Negeri Sembila
Pahang
Perak
Perlis
Pulua Pinang
Sabah
Sarawak
Selangor
Singapore
Terengganu

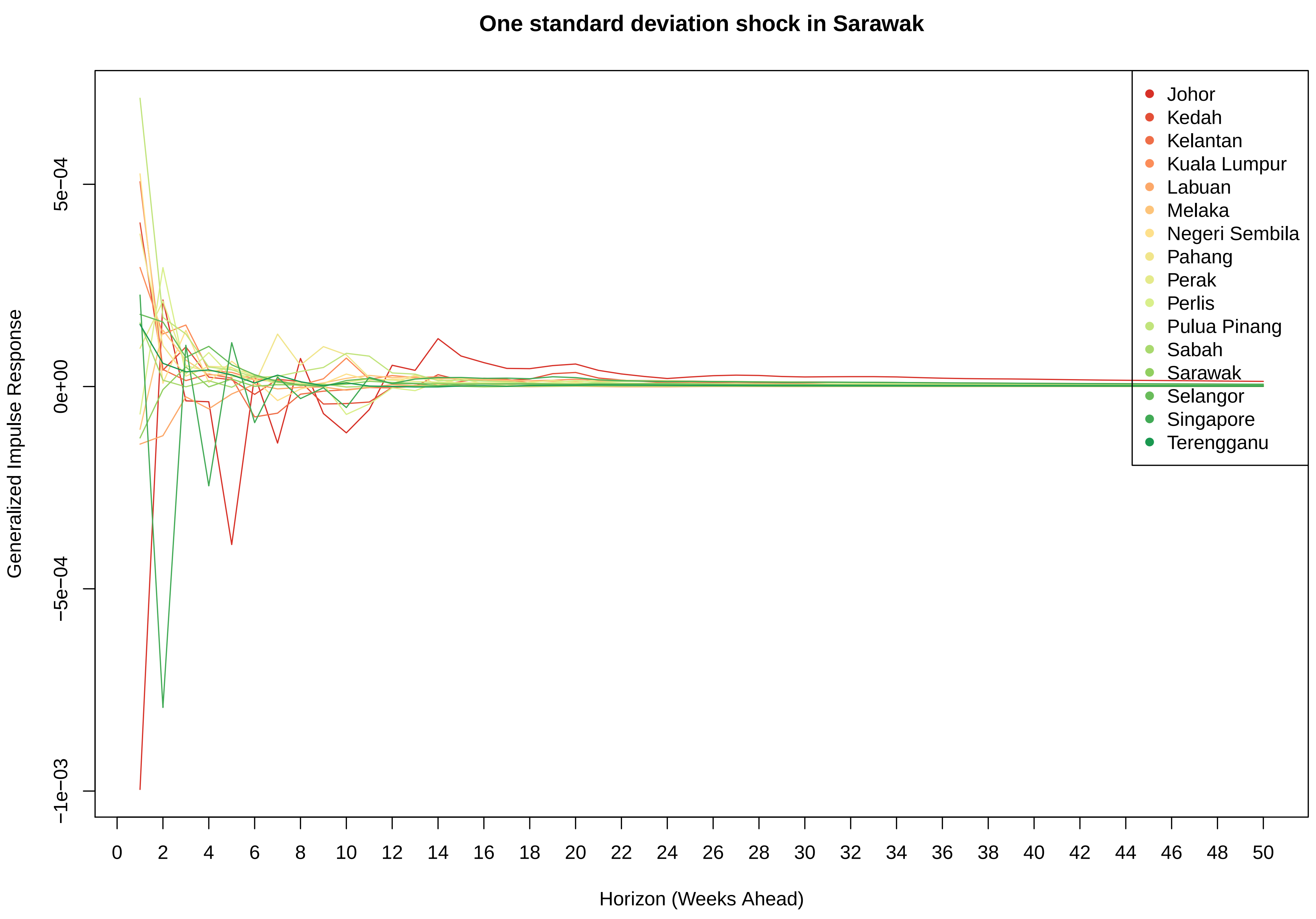

One standard deviation shock in Sarawak
Generalized Impulse Response
Horizon (Weeks Ahead)
5e−04
0e+00
−5e−04
−1e−03
0
2
4
6
8
10
12
14
16
18
20
22
24
26
28
30
32
34
36
38
40
42
44
46
48
50
Johor
Kedah
Kelantan
Kuala Lumpur
Labuan
Melaka
Negeri Sembila
Pahang
Perak
Perlis
Pulua Pinang
Sabah
Sarawak
Selangor
Singapore
Terengganu

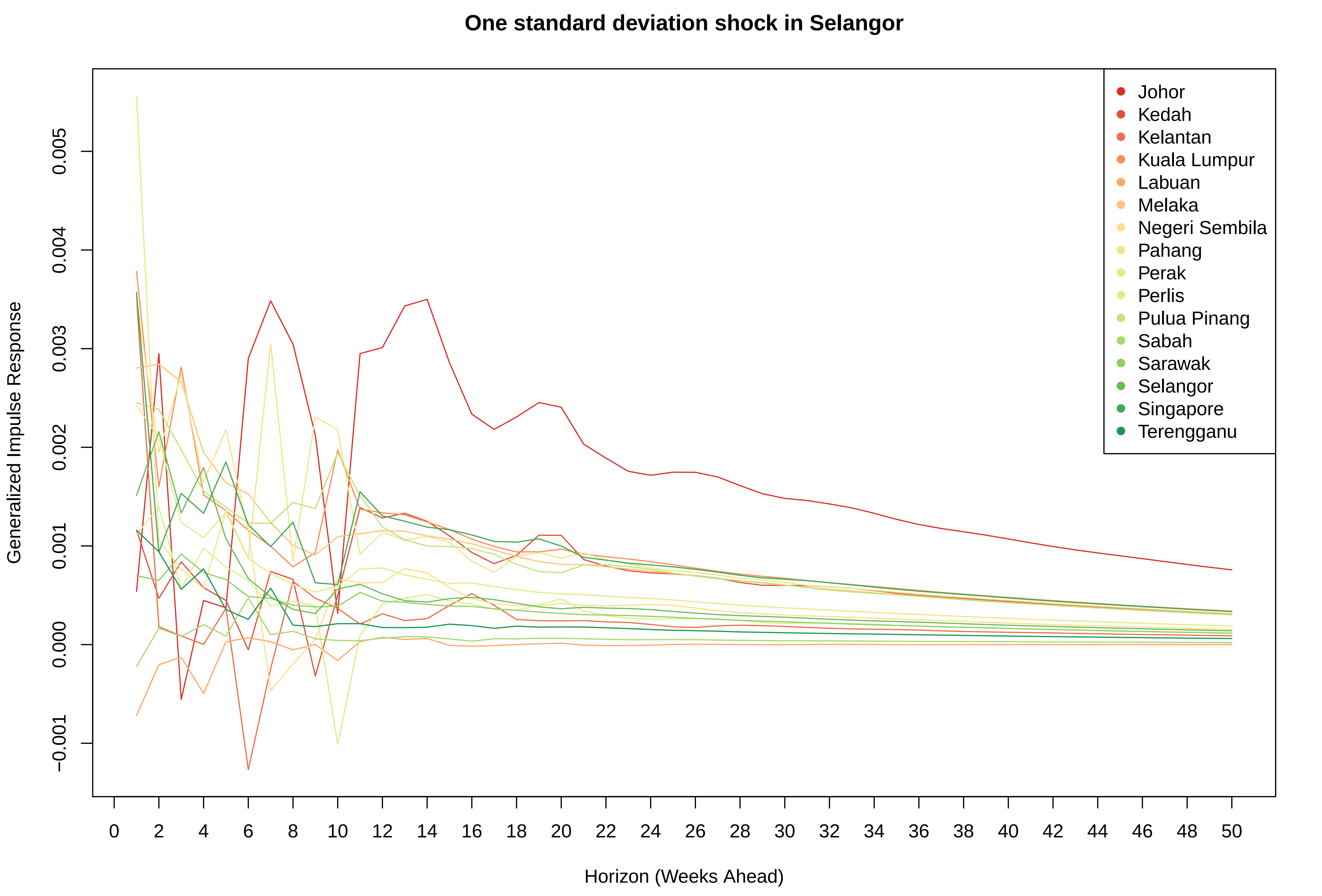

One standard deviation shock in Selangor
Generalized Impulse Response
Horizon (Weeks Ahead)
−0.001
0.000
0.001
0.002
0.003
0.004
0.005
0
2
4
6
8
10
12
14
16
18
20
22
24
26
28
30
32
34
36
38
40
42
44
46
48
50
Johor
Kedah
Kelantan
Kuala Lumpur
Labuan
Melaka
Negeri Sembila
Pahang
Perak
Perlis
Pulua Pinang
Sabah
Sarawak
Selangor
Singapore
Terengganu

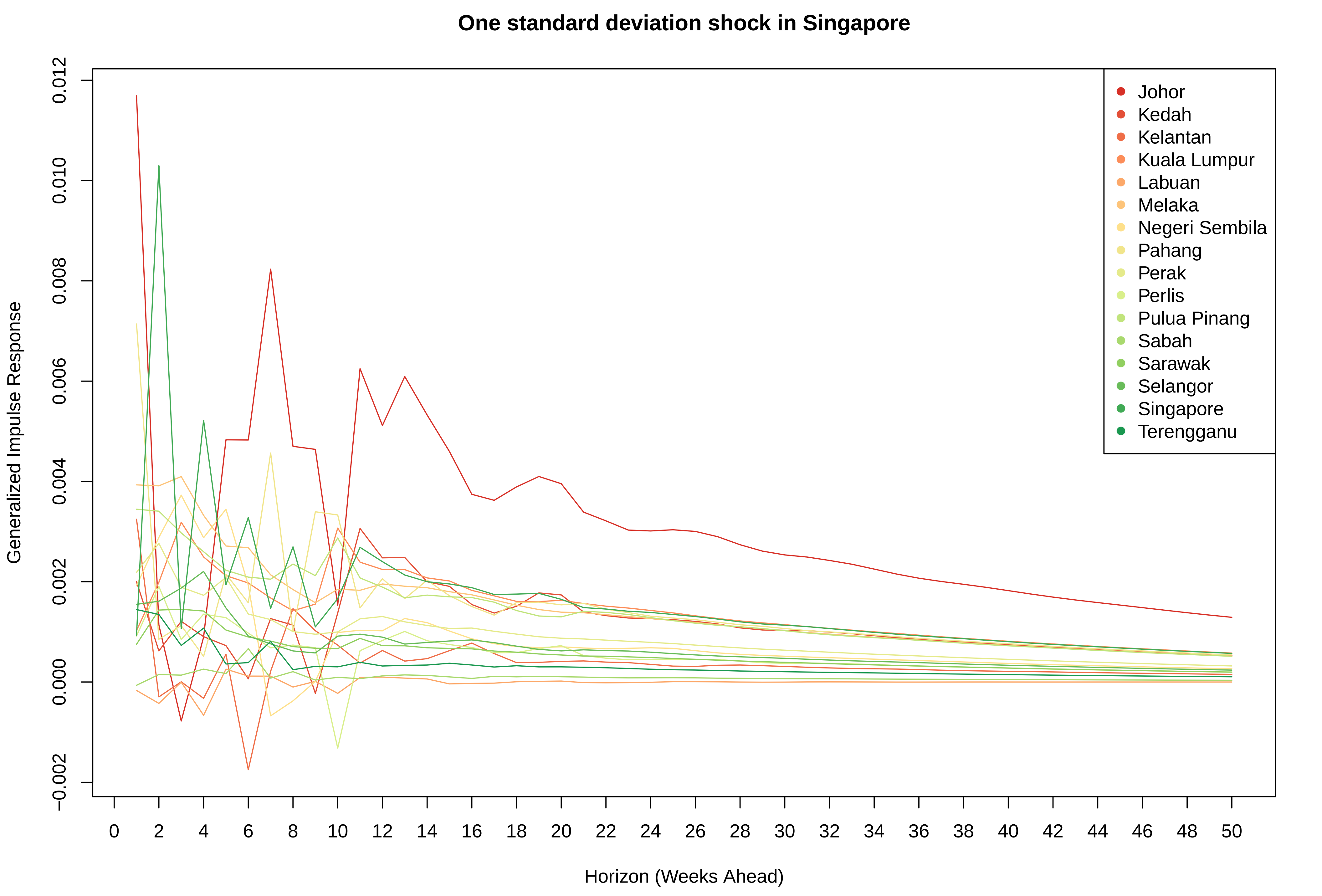

One standard deviation shock in Singapore
Generalized Impulse Response
Horizon (Weeks Ahead)
−0.002
0.000
0.002
0.004
0.006
0.008
0.010
0.012
0
2
4
6
8
10
12
14
16
18
20
22
24
26
28
30
32
34
36
38
40
42
44
46
48
50
Johor
Kedah
Kelantan
Kuala Lumpur
Labuan
Melaka
Negeri Sembila
Pahang
Perak
Perlis
Pulua Pinang
Sabah
Sarawak
Selangor
Singapore
Terengganu

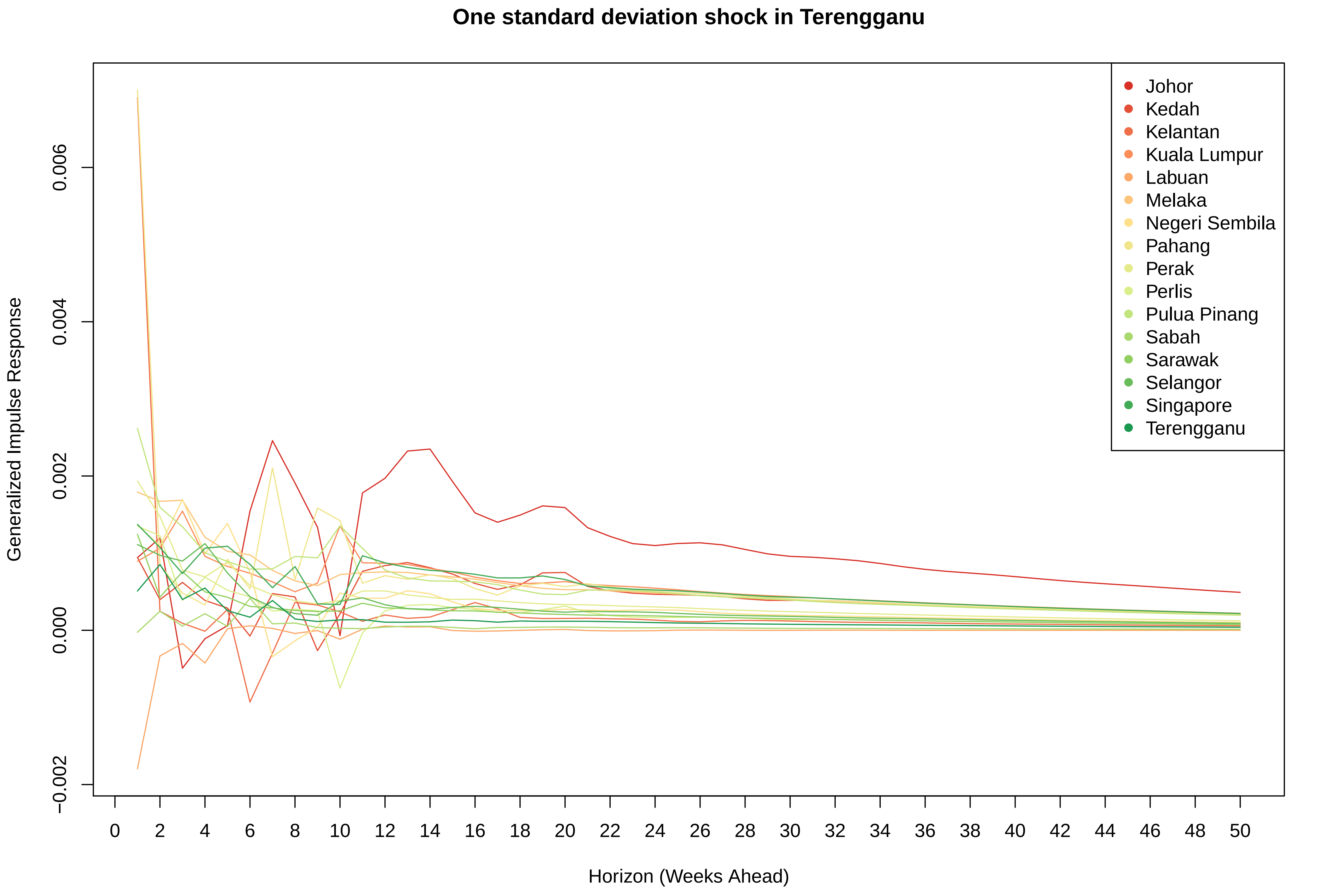
One standard deviation shock in Terengganu
Generalized Impulse Response
Horizon (Weeks Ahead)
−0.002
0.000
0.002
0.004
0.006
0 2 4 6 8 10 12 14 16 18 20 22 24 26 28 30 32 34 36 38 40 42 44 46 48 50
Johor
Kedah
Kelantan
Kuala Lumpur
Labuan
Melaka
Negeri Sembila
Pahang
Perak
Perlis
Pulua Pinang
Sabah
Sarawak
Selangor
Singapore
Terengganu
